# Dietary Flavonoid Intake and Cancer Mortality: A Population-Based Cohort Study

**DOI:** 10.3390/nu15040976

**Published:** 2023-02-15

**Authors:** Yanjun Zhou, Ke Gu, Fengying Zhou

**Affiliations:** 1 Department of Radiotherapy and Oncology, The Affiliated Hospital of Jiangnan University, Wuxi 214062, China; 2 Department of Breast Diseases, Wuxi Maternal and Child Health Hospital, Women’s Hospital of Jiangnan University, Jiangnan University, Wuxi 214000, China

**Keywords:** flavonoid, flavonol, cancer mortality

## Abstract

Cancer is a leading cause of death worldwide, posing a huge burden upon society and individuals. The adequate intake of fruit and vegetables is reported to be an effective strategy for primary cancer prevention. Fruits and vegetables are rich in nutrients, such as vitamins and flavonoids, which may reduce the occurrence and progression of cancers. However, the importance of each flavonoid and the sub-classes remains controversial regarding cancer mortality. The population benefiting from increased flavonoid intake has not been determined. An estimation of cancer mortality by flavonoid intake is not established. We explored the association between the intake of flavonoids and cancer mortality amongst 14,029 participants in the National Health and Nutrition Examination Survey. During a median follow-up of 117 months, 405 cancer deaths were confirmed. Being in the second, third, and fourth quartiles of flavonol intake, the cancer mortality was inversely associated with the intake of flavonols (multivariate analysis HR (95% CI] 0.58 [0.36, 0.91], *p* = 0.02, Q1 vs. Q2; 0.55 [0.31, 0.96], *p* = 0.04, Q1 vs. Q3; 0.54 [0.30, 0.99], *p* = 0.05, Q1 vs. Q4, respectively). Potential effects of dietary flavonol intake against cancer death was observed especially in participants aged 50 or above, males, whites, former smokers, people who used to drink or drink alcohol mildly, people without hyperlipidemia, and people with hypertension. Moreover, the dietary intakes of peonidin, naringenin, and catechin were inversely associated with cancer mortality (multivariate HR [95% CI] 0.93 [0.88,0.98], *p* = 0.01; 0.97 (0.95,1.00), *p* = 0.03; 0.98 (0.96,1.00), *p* = 0.05, respectively). Furthermore, a nomogram based on flavonol intake is feasible for assessing cancer mortality for each participant. Taken together, our results could improve personalized nutrition amongst cancer patients.

## 1. Introduction

Cancer is a disease resulting from the uncontrolled growth and division of abnormal cells in the body and is the leading cause of death and an important obstacle to extending life expectancy worldwide [1]. It was estimated that cancer ranked as the first or second leading cause of death in the population aged below 70 years in more than 60% of countries and ranked third or fourth in the remaining countries in 2019 [1]. There were an estimated 19.3 million new cases and 10 million cancer deaths in 2020 [1]. Primary prevention is a pivotal strategy with which to decrease the growing burden of cancer. However, establishing high-quality cancer prevention remains a challenge [1]. Nearly one-third of cancer-related deaths could be prevented using balanced dietary improvements in the US [2]. The adequate consumption of fruit and vegetables presented the considerable potential to reduce the occurrence and progression of cancer [3,4]. Dietary flavonoids are a group of natural polyphenols, present in fruit, cereal, vegetables, tea, and red wine [2]. Two benzene rings connected by a heterocyclic pyran ring consist of the basic—structure of flavonoids [5]. Flavonoids are subcategorized into six subclasses based on the linking arrangement and the saturation of the double bond on the pyran structure, including anthocyanins, flavan-3-ols, flavanones, flavones, flavonols, and isoflavones [5]. Studies in vitro demonstrated that flavonoids could induce apoptosis and inhibit cell proliferation and metastasis by targeting the key molecules and signaling pathways in various tumor cells [6,7,8,9]. However, the results of epidemiological studies on the association between dietary flavonoid intake and cancer mortality remain inconsistent. Some previous studies on total flavonoid intake and all-cause cancer mortality did not reveal the inverse association [10,11]. More recent research demonstrated a beneficial association between total dietary flavonoid intake and cancer mortality [12,13]. Moreover, whether people with an unhealthy lifestyle could benefit from increased dietary flavonoid intake needs to be elucidated.

To explore the association between dietary flavonoid intake and cancer-related mortality, we downloaded all the publicly available data in the Database of Flavonoid Values for USDA Food Codes and Flavonoid Intake Data Files from What We Eat in America (WWEIA), National Health, and Nutrition Examination Survey (NHANES) [14]. Until 16 December 2022, only the data in 2007-2010 and 2017-2018 were released by the USDA [14]. Therefore, our study explored the association between dietary flavonoid intake and cancer-related mortality in 2007-2010 and 2017-2018, which included all the publicly accessible data [14]. We revealed that in comparison to being in the first quartile of dietary flavonol intake, being in the second, third, and fourth quartile was inversely associated with cancer-related mortality. Furthermore, potential protective effects of dietary flavonol intake against cancer death was observed, especially in former smokers, mild drinkers, and people who used to drink, which suggests that changing an unhealthy lifestyle is also important. A nomogram based on dietary flavonol intake is clinically adaptable to assessing cancer mortality among individuals.

## 2. Materials and Methods

NHANES is a program of studies designed to evaluate the health and nutritional status of adults and children in the US. NHANES is a program of the National Center for Health Statistics, which is part of the Centers for Disease Control and Prevention [15]. The NHANES directors can be accessed at [16]. The protocols for NHANES surveys were approved by the NCHS Ethics Review Board. The approval number for the survey year cycles 2007-2008 and 2009-2010 was the Continuation of Protocol #2005-06. The approval numbers for the survey year cycle 2017–2018 were the Continuation of Protocol #2011-17 (Effective through 26 October 2017) and Protocol #2018-01 (Effective beginning 26 October 2017) [17]. All participants signed a written informed consent form.

All data in our study are publicly available and without personally identifiable information. All analyses were conducted following the relevant guidelines and regulations [18]. The survey process consisted of two steps. Demographic and health-related information was collected in the homes of participants during the first interview. After that, a standardized physical examination was carried out in a mobile examination center two weeks later, as well as a blood draw,24-h dietary recall, and other investigations, such as laboratory analysis of urine, microbiome sampling of the oral cavity, and so on. We obtained 18,538 participants aged 18 years or above in the continuous NHANES cycles of 2007–2010 and 2017–2018 with complete mortality information: among them were 14,490 participants with complete data from the dietary flavonoid intake assessment.

### 2.1. Dietary Flavonoid Intake Assessment

The dietary flavonoid values in our study were obtained from the database of flavonoid values for USDA Survey Foods and Beverages (flavonoid database for short), which was established in 2003–2004 [19]. The flavonoid database provides the flavonoid values in foods and beverages in the USDA Food and Nutrient Database for Dietary Studies (FNDDS) [20] and corresponding dietary data from WWEIA [21] and NHANES. The amounts of 29 flavonoids (mg/100 g) in each food/beverage were determined by the USDA Nutrient Data Laboratory [22]. The dietary intake of flavonoids was calculated on days 1 and 2, including the six main flavonoid subclasses commonly consumed in the US diet, namely total anthocyanins (cyanidin, delphinidin, malvidin, pelargonidin, peonidin, and petunidin), total flavan-3-ols ((-)-epicatechin, (-)-epicatechin 3-gallate, (-)-epigallocatechin, (-)-epigallocatechin 3-gallate, (+)-catechin, (+)-gallocatechin, theaflavin, theaflavin-3,3′-digallate, theaflavin-3′-gallate, theaflavin-3-gallate, and thearubigins), total flavanones (eriodictyol, hesperetin, and naringenin), total flavones (apigenin and luteolin), total flavonols (isohamnetin, kaempferol, myricetin, and quercetin), total isoflavones (diadzein, genistein, and glycitein), and subtotal catechins ((-)-epicatechin, (-)-epicatechin 3-gallate, (-)-epigallocatechin, (-)-epigallocatechin 3-gallate, (+)-catechin, and (+)-gallocatechin). The retention factors for cooked foods were introduced in the estimation of the flavonoid amounts. For moist-heat cooking, a loss of 50% was applied to anthocyanidins, as well as one reduction of 15% to flavonols, flavan-3-ols, flavanones, and flavones. No retention factors were implemented for isoflavones and dry heat cooking [20]. The association between individual flavonoid intake grouped by flavonoid subclasses was analyzed using the Pearson correlation method.

Based on the stratified and multistage probability sampling designed in the NHANES, we used the mean of the two-day intake of each flavonoid, as well as the weights “wtdr2d” constructed for participants who completed two days of dietary recall in making estimates representative of the US non-hospitalized population [18].

### 2.2. Mortality Ascertainment

The updated follow-up date was 31st December 2019. A total of 14,092 participants with an available survival status were included in our study. The time in months from the household interview to mortality was assessed as the follow-up time. A death caused by cancer was ascertained by the National Death Index and International Classification of Diseases-10 C00-C97 as a malignant neoplasm. A potential nonlinear association between total flavonol intake and cancer mortality was evaluated using restricted cubic splines. The association between flavonoid intake and cancer mortality was assessed using Cox proportional hazards analysis.

### 2.3. Covariate Assessment

Covariates, i.e., age, ethnicity, education, marital status, poverty income ratio (PIR), smoking status, alcohol use, and physical activity (PA), were collected via questionnaires. Educational status was classified according to the number of years of education as <9 years, 9–12 years, and >12 years. Marital status was divided into partnered and unpartnered. The body mass index (BMI) was calculated as weight (kg)/height squared (m^2^). The term “never” regarding the smoking status was defined as fewer than 100 cigarettes during life; “former” as more than 100 cigarettes during life and not at all currently smoking; and “now” as more than 100 cigarettes during life and smoking some days or every day. The classification of alcohol usage was as described in [23]. The healthy eating index (HEI) was calculated based on the 2015 version using the sum of food intake on days 1 and 2 for each participant [24]. The dietary inflammatory index (DII) was calculated as described in [25]. We used the total time and the total metabolic equivalent (MET) of PA for one week.

Regarding the perspectives of disease diagnosis, hyperlipidemia was defined as meeting one of the following conditions: triglyceride ≧ 150 mg/dL, low-density lipoprotein ≧ 130 mg/dL, high-density lipoprotein < 140 ng/dL, or usage of lipid-lowering drugs. Cardiovascular disease was defined as having ever had a heart attack or stroke. Participants were diagnosed as having the chronic obstructive pulmonary disease (COPD) when meeting one of the following conditions: the value of forced expiratory volume at first second/forced vital capacity (FEV1/FVC) < 0.7 after the application of a bronchodilator; having been told you had emphysema; using COPD drugs selective phos-phodiesterase-4 inhibitors, mast cell stabilizers leukotriene modifiers, and inhaled corticosteroids. Participants were diagnosed as having asthma when meeting one of the following conditions: having been told you had asthma; had an asthma attack; the application of selective phos-phodiesterase-4 inhibitors, or mast cell stabilizers leukotriene modifiers and inhaled corticosteroids. Participants with both COPD and asthma were coded as ACO. Stroke history was defined as having ever had a stroke. Cancer history was defined as having ever had cancer. Average blood pressure was calculated as [26], and participants were diagnosed as having hypertension when meeting one of the following conditions: systolic pressure≧ 140 mmHg or diastolic pressure ≧ 90 mmHg on three occasions. Participants were diagnosed as having type 2 diabetes mellitus (DM) when meeting one of the following conditions: having been told you had diabetes; HbA1c ≧ 6.5%, fasting glucose ≧ 7.0 mmol/L, glucose ≧ 11.1 mmol/L, oral glucose tolerance test ≧ 11.1 mmol/L; or usage of antidiabetic drugs. Missing covariates were imputed by the R package “mice”.

### 2.4. Flavonoid Supplement Identification

All dietary flavonoid supplement names and ingredients from the NHANES database were text-mined for key phrases to identify the products. Search terms used to identify flavonoid supplements and relevant results are shown in Supplementary Table S1.

### 2.5. Urinary Isoflavone Metabolite Assessment

The levels of isoflavone metabolites, including daidzein (ng/mL), equol (ng/mL), genistein (ng/mL), and O-desmethylangolensin (O-DMA, ng/mL) in the urine, were obtained from the NHANES, which were measured by high-performance liquid chromatography-atmospheric pressure photoionization-tandem mass spectrometry. Only the data in the year 2007–2010 were publicly accessible in the survey years of our study. The association between urinary isoflavone metabolite levels and cancer-related mortality was analyzed using Cox proportional hazards analysis. Meanwhile, the special weights “wtsb2yr” or “wtsa2yr” for special urine examination were employed in the Cox analysis.

### 2.6. Statistical Analysis

The R packages “NHANESR” and “survey” were employed for data preparation and statistical analysis. Continuous variables are demonstrated as the mean ± standard deviation, median, and percentile range, and categorical variables are presented as percentages. The potential nonlinear association was evaluated using restricted cubic splines with the R package “rms”. Cox proportional hazards models were used to calculate hazard ratios (HRs) and 95% confidence intervals (95% CIs). A *p* value < 0.05 was used as a cut-off for statistical significance. A weighted survey nomogram with the intake of flavonols was established and validated via a calibration curve for predicting the 10-year survival probability amongst participants using the R packages “rms” and “SvyNom” [27]. All analyses were conducted using R software (version 4.1.3, the R Foundation for Statistical Computing).

## 3. Results

### 3.1. Characteristics of Flavonoid Intake

A total of 14,029 participants with complete survival information, intake of flavonoids, and relevant survey weights were included in our study. The minimum, 25th percentile, median, mean, 75th percentile, and maximum of each flavonoid intake were listed in Table 1.

**Figure 1:**
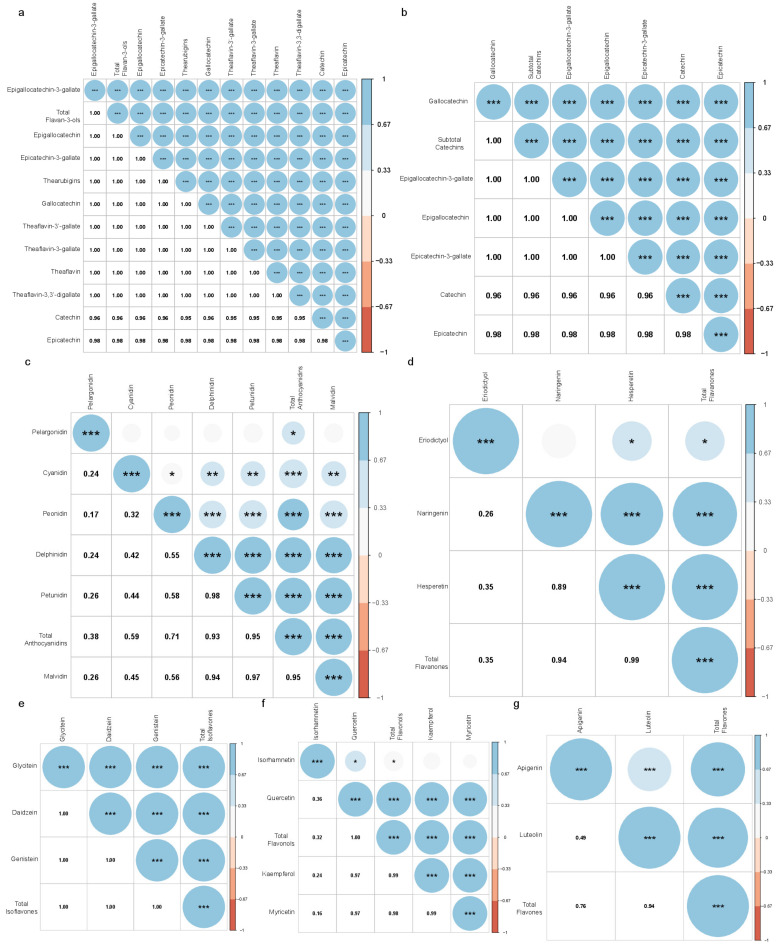
The association between individual flavonoids by the main subclasses. The association between individual flavan-3-ols (**a**), catechins (**b**), anthocyanidins (**c**), flavanones (**d**), isoflavones (**e**), flavonols (**f**), and flavones (**g**) are shown in heatmaps. Correlation was examined by Pearson’s correlation. The Pearson’s correlation coefficients are represented by the color bar. The *p* values of the correlation are represented by the size of the circle and the asterisks. *** *p* < 0.001, ** 0.001 ≦ *p* < 0.01, * 0.01 ≦ *p* < 0.05.

In addition, the associations between individual flavonoids were assessed based on the main subclasses of flavonoid. The intakes of individual flavan-3-ol, catechins, isoflavones, and flavones were strongly associated with each other (*p* < 0.001, Figure 1a,b,e,g). Except for the intake of pelargonidin, the individual anthocyanidin was significantly associated with each other (*p* < 0.05, Figure 1c). The intakes of hesperetin and naringenin were strongly associated with each other (*p* < 0.001, Figure 1d). The intake of eriodictyol showed a significant association with the intake of hesperetin and showed no correlation to naringenin (Figure 1d). Except for the intake of isorhamnetin, the intake of the rest of the flavonols was strongly associated with each other (Figure 1f). The results above might suggest that the intake of pelargonidin, eriodictyol, and isorhamnetin may have a different dietary origin from the subclasses to which they belong. For example, fruits are divided into groups based on the types of anthocyanin aglycones, i.e., pelargonidin group, cyanidin/peonidin group, and multiple anthocyanins group. The pelargonidin group mainly contains strawberries [28].

### 3.2. Baseline Characteristics of the Cohort

A total of 405 (2.97%) cancer-related deaths were ascertained over the follow-up period by 31st December 2019. The characteristics of death cases caused by cancer are summarized in Table 1. Compared to those who were alive, participants who died of cancer were older (65.93 ± 0.89, *p* < 0.0001); more frequently male (57.30%, *p* < 0.001); more frequently white (77.71%, *p* = 0.002); with less than 12 years of education (*p* < 0.0001); poorer (with lower PIR, 2.65 ± 0.15, *p* < 0.0001); with less daily energy intake (3828.29 ± 112.64 kcal, *p* = 0.01); with a higher DII (1.77 ± 0.12, *p* = 0.02); less so non-smokers (*p* < 0.0001); and more so former alcoholics (*p* < 0.0001) (Table 1). In addition, deceased participants had a higher prevalence of DM (38.79%, *p* < 0.0001), hypertension (73.80%, *p* < 0.0001), CVD (34.16%, *p* < 0.0001), cancer (19.47%, *p* < 0.001), and respiratory diseases (32.40%, *p* < 0.0001). Most of those who died of cancer depicted a low dietary intake of daidzein, genistein, glycitein, petunidin, delphinidin, malvidin, peonidin, epicatechin, naringenin, luteolin, kaempferol, myricetin, total isoflavones, total anthocyanidins, total flavones, and total flavonols (Table 2).

Due to the seven-year gap in the data available, the baseline characteristics of the study population are compared according to the survey year cycles in Table 3. There was an increase in the dietary intake of genistein, glycitein, petunidin, peonidin, and total isoflavones, and total anthocyanidins during the 2007–2008, 2009–2010, and 2017–2018 year cycles. Dietary flavonoid intake first increased in 2009–2010 and then decreased in 2017–2018 compared to 2007–2008, e.g., cyanidin, delphinidin, eriodictyol, hesperetin, apigenin, quercetin, and total flavanones. Dietary intake of the remaining flavonoids stayed the same across the different year cycles (Table 3). Moreover, there was an increase in the frequency of participants with more than 12 years of education, the frequency of non-smokers, the frequency of participants without respiratory diseases, and BMI during the different year cycles (Table 3). The highest total score regarding the HEI occurred in 2009–2010, while the lowest DII, total time of PA, and total MET of PA were present in 2009–2010 (Table 3). The incidence of hyperlipemia and the frequency of non-alcoholics decreased during the three-year cycles (Table 3).

### 3.3. Dietary Flavonoid Intake

In addition, we compared the age-adjusted and gender-adjusted mean dietary intake of flavonoid subclasses across the ethnic groups using linear regression. Relative to white participants, black participants had a lower mean catechin intake (mean difference 30.28 mg/day, *p* < 0.0001), lower mean isoflavone intake (0.72 mg/day, *p* = 0.01), lower mean anthocyanidin intake (7.14 mg/day, *p* < 0.0001), lower mean flavan-3-ol intake (72.57 mg/day, *p* < 0.0001), higher mean flavanone intake (3.40 mg/day, *p* < 0.0001), lower mean flavone intake (0.37 mg/day, *p* < 0.0001), lower mean flavonol intake (4.62 mg/day, *p* < 0.0001), and lower mean flavonoid intake (82.02 mg/day, *p* < 0.0001) (Table 4). Similarly, Mexican-American participants had a lower mean catechin intake (35.20 mg/day; *p* < 0.0001), lower mean anthocyanidin intake (6.09 mg/day, *p* < 0.001), lower mean flavan-3-ol intake (98.11 mg/day, *p* < 0.0001), higher mean flavanone intake (5.55 mg/day, *p* < 0.0001), and lower mean flavonol intake (3.70 mg/day, *p* < 0.0001), and lower mean flavonoid intake (102.34-mg/day, *p* < 0.0001), in comparison to white participants (Table 4). Other ethnic participants had a lower mean flavan-3-ol intake (34.54 mg/day, *p* = 0.02), higher mean flavanone intake (4.00 mg/day, *p* < 0.001), and lower mean flavonoid intake (32.55 mg/day, *p* = 0.03), in comparison to white participants (Table 4). In addition, the intake of flavonoid subclasses was stratified by age, gender, and ethnicity (Table 4).

### 3.4. Associations between Dietary Flavonoid Intake and Cancer-Related Mortality

**Figure 2:**
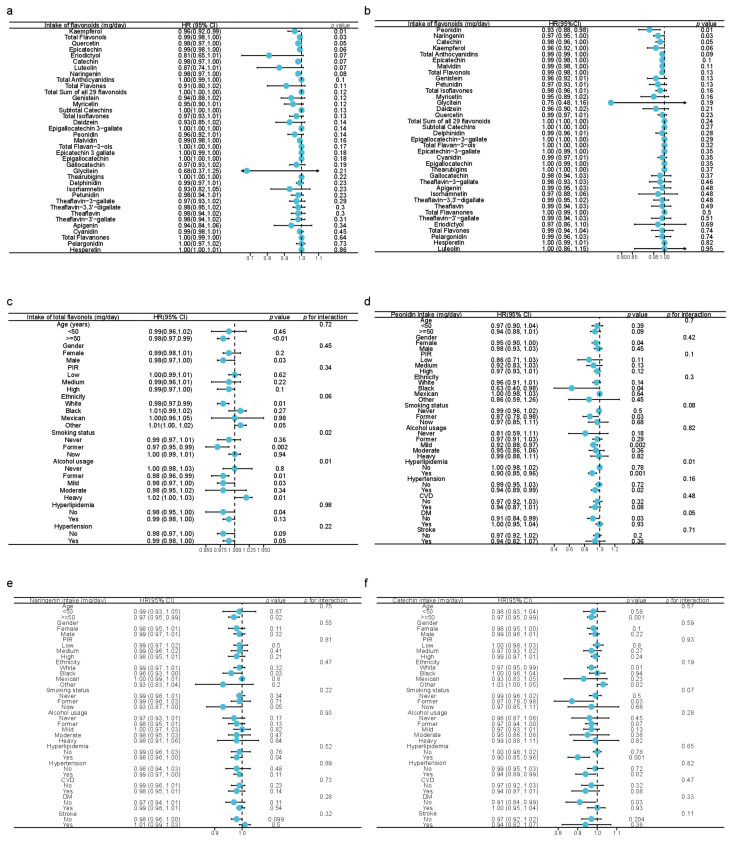
Forest plots in the upper panel display the association between dietary flavonoid intake and cancer-related mortality using univariate (**a**) and multivariate (**b**) analyses adjusted for age, ethnicity, gender, PIR, educational status, marital status, daily energy intake, alcohol consumption, smoking status, cancer history, a total score of HEI, DII, and a total time of PA. (**c**) Forest plot showing the association between cancer-related mortality and total dietary flavonol intake using unadjusted Cox analysis stratified by age, gender, PIR, ethnicity, smoking status, alcohol usage, and disease history. Forest plot showing the association between cancer-related mortality and the dietary intake of peonidin (**d**), naringenin (**e**), and catechin (**f**) using unadjusted Cox analysis stratified by age, gender, smoking status, alcohol usage, and disease history. HR: hazard ratio; PIR: poverty income ratio; HEI: healthy eating index, 2015 version; DII: dietary inflammatory index; PA: physical activity.

To identify whether dietary flavonoid intake was independently associated with cancer-related mortality, univariate and multivariate Cox analyses were employed in Figure 1a,b. As a result, the univariate Cox model demonstrated that the dietary intake of kaempferol, quercetin, and total flavonols was inversely associated with cancer mortality (Figure 2a). The dietary intakes of peonidin, naringenin, and catechin were inversely associated with cancer mortality after adjustment for age, ethnicity, gender, PIR, educational status, marital status, daily energy intake, alcohol consumption, smoking status, cancer history, total score of HEI, DII, and a total time of PA (Figure 2b). The analysis using restricted cubic splines revealed a monotonically decreasing association between dietary intakes of peonidin, naringenin, and catechin and cancer mortality (Supplementary Figure S1b–d). Because of the heterogeneity of the population, we further stratified the population by different characteristics and calculated the interactions, including age, gender, PIR, ethnicity, smoking status, alcohol usage, hyperlipidemia, and hypertension. In the stratified analysis, the inverse association of flavonol intake against cancer death was observed, especially in participants aged 50 or above, males, whites, former smokers, ex-drinkers, mild drinkers, people without hyperlipidemia, and people with hypertension, while the positive correlation was observed in heavy drinkers and other races (Figure 2c). Notably, flavonols might have the best benefits for former smokers (*p* for interaction = 0.02), as well as mild-alcoholics and former alcoholics (*p* for interaction = 0.01) against cancer death. Besides, the intake of flavonols tended to influence cancer mortality differently amongst different ethnicities (*p* for interaction = 0.06), which might explain the observation that the intake of flavonols turned out to be a risk factor for cancer death in other minority races [1.01(1.00, 1.02), *p* = 0.05] (Figure 2c). Moreover, the benefits of the increased intake of peonidin against cancer mortality might occur amongst females, blacks, past-smokers, mild-alcoholics, people with hyperlipidemia, people with hypertension, and people without DM (Figure 2d). The effect of peonidin on cancer mortality differs in people with and without hyperlipidemia (*p* = 0.01, Figure 2d). Similarly, the potential protective effects of naringenin against cancer mortality were observed in people aged 50 or above, blacks, and participants with hyperlipidemia (Figure 2e). The inverse association between the intake of catechin and cancer mortality was shown in people aged 50 or above, ex-smokers, people with hyperlipidemia or hypertension, and people without DM (Figure 2f).

**Figure 3:**
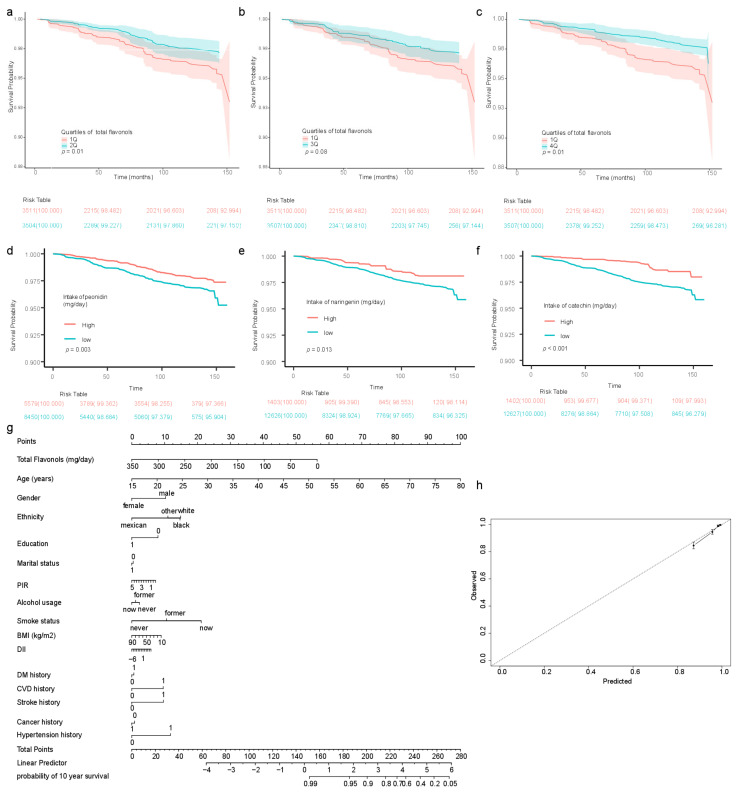
The survival curves of participants and the related nomogram. Comparison of survival probabilities for participants with dietary flavonol intake in the first quartile and second quartile (**a**); in the first quartile and third quartile (**b**); in the first quartile and fourth quartile (**c**). Kaplan–Meier analyses of survival probabilities based on the 60th percentage value of dietary peonidin intake (**d**). Kaplan–Meier analyses of survival probabilities based on the 90th percentage value of dietary naringenin intake (**e**). Kaplan–Meier analyses of survival probabilities based on the 90th percentage value of dietary catechin intake (**f**). Nomogram established with a total dietary intake of flavonols (**g**). Calibration curve to assess the consistency of the predicted survival possibility at ten years via the nomogram with total dietary flavonol intake (**h**). BMI: body mass index; COPD: chronic obstructive pulmonary disease; DM: type 2 diabetes mellitus. Regarding the disease prevalence, 0 indicates “no” and 1 indicates “yes”. Participants with less than 12 years of education were coded as 0; those with 12 years of education or above were coded as 1. As for marital status, participants with a partner were coded as 1; participants without a partner were coded as 0. PIR: poverty income ratio.

The dietary intake of flavonoid subclasses was divided by quartiles to further analyze the relationship between cancer mortality and the dietary intake of flavonoid subclasses (Table 5). Notably, the increased dietary intake of flavonols tended to be inversely associated with cancer-related mortality (multivariate analysis HR (95% CI] 0.82 (0.67, 1.02), *p* for trend = 0.08). Being in the second, third, and fourth quartiles of flavonol intake, the cancer mortality was inversely reduced compared with that in the first quartile (multivariate analysis HR (95% CI] 0.58 [0.36, 0.91], *p* = 0.02, Q1 vs. Q2; 0.55 [0.31, 0.96], *p* = 0.04, Q1 vs. Q3; 0.54 [0.30, 0.99], *p* = 0.05, Q1 vs. Q4, respectively) (Table 5). Participants in the second and fourth quartiles of dietary flavonol intake had a higher survival probability than those in the lowest quartile (Q1 vs. Q2 *p* = 0.01, Figure 3a; Q1 vs. Q4 *p* = 0.01, Figure 3c). Participants in the third quartile tended to have a higher survival probability than those in the first quartile (*p* = 0.08, Figure 3b). The analysis using restricted cubic splines revealed a monotonically decreasing association between dietary flavonol intake and cancer mortality (Supplementary Figure S1a). Moreover, the survival probability was significantly higher for participants with a peonidin intake greater than the 60th percentile than for those with an intake below the 60th percentile (Figure 3d). Similarly, the survival probability was significantly higher for participants with a naringenin intake greater than the 90th percentile than for those with intake below the 90th percentile (Figure 3e). The survival probability was significantly higher for participants with a catechin intake greater than the 90th percentile than for those with intake below the 90th percentile (Figure 3f). In addition, being in the second quartile of dietary flavone intake was inversely associated with cancer-related mortality in comparison to being in the first quartile (0.48 [0.26, 0.87], *p* = 0.02) (Table 5). As 37.92% of participants had no dietary isoflavone intake, the cohort was divided into two groups based on the median dietary intake of isoflavones (Table 5).

### 3.5. Establishment of Nomogram with Total Dietary Flavonol Intake

We then built an easy-to-use and clinically adaptable risk nomogram for predicting the survival probability at ten years (Figure 3g). A higher total score was associated with a lower 10-year survival rate. The predictions made by the nomogram model were close to the observed outcomes of 10-year survival (Figure 3h).

### 3.6. Associations between Isoflavone Metabolites in Urine and Cancer-Related Mortality

Since the bioavailability of flavonoids is low, the flavonoid intake might not accurately reflect the effect of flavonoids utilized by the body on cancer mortality. We considered using urinary flavonoid and their metabolite levels to explore the correlation with cancer mortality. Due to data access privileges, we were only able to obtain urinary levels of isoflavones and relevant metabolites in 2007–2010 during our study period. As shown in Table 6, there was no association between the levels of daidzein, ODMA, equol, and genistein and cancer mortality.

## 4. Discussion

The global cancer burden is expected to increase by 47% to 28.4 million cases by 2040 in comparison with 2020 [1]. Primary prevention prioritizes reducing the personal, clinical, and socioeconomic burden of cancer. It is estimated that approximately one-third of cancers can be attributed to diet, nutrition, and physical activity in developed countries [29]. The adequate intake of fruit and vegetables, enriched in flavonoids, has been recommended to prevent cancers [29]. Recent epidemiological studies have indicated that the intake of flavonoids is inversely associated with cancer mortality [12,13]. Flavonoids can be categorized into six main subclasses, namely anthocyanidins, flavan-3-ols, flavanones, flavones, flavonols, and isoflavones. Among them, flavonols, mainly including quercetin, kaempferol, myricetin, and isorhamnetin, are present in tea, onions, and berries.

Our results demonstrated that the inverse association between flavonol intake and cancer mortality is coincidental with Danish results [13]. Interestingly, differing from the result that the intake of flavonols was inversely associated with the risk of lung cancer amongst male smokers [30], the potential protective effects of flavonol were observed amongst former smokers, former drinkers, and mild-drinkers in our study, which may suggest that quitting smoking and alcohol consumption might have priority in reducing cancer death. It is well-documented that flavonols play an important role in the carcinogenesis and progression of cancer in vitro. Kaempferol can inhibit the epithelial-mesenchymal transition and induce apoptosis and cell cycle arrest in the G2/M phase by targeting phosphoinositide 3-kinase/protein kinase B signaling pathways [31]. Quercetin is accountable for suppressing proliferation by causing cell cycle arrest in the G1 phase through targeting cyclins [32,33]. Moreover, quercetin at a high concentration can inhibit cell cycle progression from G0/G1 to G2/M [25,26]. Besides influencing the cell cycle, quercetin can induce apoptosis through pro-apoptotic PI3K/Akt and mitogen-activated protein kinase signaling pathways [34,35]. Similar to other flavonols, myricetin can induce apoptosis, interfere with the cell cycle, and inhibit cell proliferation in cancer cells [36]. As an antioxidant, myricetin can scavenge the elevated free radicals and reactive oxygen species in cancer cells [36,37,38]. Myricetin can restrain cancer progression by downregulating the levels of various inflammatory markers [39]. However, what should be paid attention to is that flavonoids could exert pro-oxidant properties at high doses [5,40,41]. Flavonoids containing phenolic rings, when oxidized by oxidases, produce cytotoxic phenoxy radicals; co-oxidize unsaturated lipids, nucleic acids, ascorbic acid, nicotinamide adenine dinucleotide, and glutathione; and lead to the formation of reactive oxygen species and mitochondrial toxicity. Quercetin and other flavonoids were reported to induce significant frequencies of sister chromatid exchange and micronuclei, as well as inhibit cell proliferation due to the pro-oxidant effects in special conditions [42,43]. In our study, we found numerous supplements were taken by people, which may not be regulated by the Food and Drug Administration and therefore the potential toxicity has not been fully assessed (Supplementary Table S1). A study on the effect of flavonoid dietary supplements on cancer survival is warranted.

A comprehensive understanding of the mechanisms of flavonoids in cancer could help define new strategies for cancer management and prevention. The bioavailability of flavonoids is affected by dietary patterns, interaction with the food matrix, host genetics, intestinal microbiota, and the phase I and II metabolism in the liver [44,45,46], which may explain the inconsistent epidemiological findings. Our observation that the intake of flavonols tended to influence cancer mortality differently amongst different ethnicities may be caused by host genetics and dietary patterns. In our study, we found a distinct flavonoid intake pattern among ethnicities. Except for the increased intake of flavanones, the intake of the remaining flavonoids was lower amongst black people than white people. The low intake of flavonoids in our study may reflect a high consumption of the Southern dietary pattern, constituted by a high consumption of added fats, fried food, organ meats, processed meats, and sugar-sweetened beverages in the US black population [47]. The focus of primary prevention should be on those groups.

Moreover, although the flavonoids have low solubility and low bioavailability [48,49], certain intestinal bacteria can degrade flavonoids or their glycosides into smaller and more bioavailable metabolites which, when absorbed, can exert their physiological effects *in vivo* [45]. For example, physiological effects of isoflavones are mainly due to their metabolites, such as equol and O-DMA, produced by intestinal microbiota [50]. In comparison to daidzein, equol has a stronger estrogenic effect, antioxidant property, and anti-androgenic effect [51,52]. However, neither the intake of isoflavone nor the urinary levels of equol and O-DMA were associated with cancer mortality in our study. It would be interesting to study their association in estrogen-related diseases.

Notably, we found that the intakes of peonidin, naringenin, and catechin are inversely associated with cancer mortality. Peonidin, present in red wine and berries, can inhibit lung cancer metastasis [53]. Naringenin, present in citrus, decreases proliferation and induces apoptosis in various cancer types [54]. Catechin, abundant in green tea, can inhibit cancer growth and progression through its anti-oxidant property, cell cycle modulation, receptor tyrosine kinase pathway downregulation, immune response regulation, and epigenetic modification control [55].

The estimated amounts of individual flavonoids in plants and foods were reported to be influenced by nonrepresentative sampling, different cultivars, different growing, and processing conditions, and analytical bias [22]. To generate the qualified data for the flavonoid database, five criteria were implemented in the flavonoid database of USDA, including the sampling plan, the number of samples, sample handling, analytical method, and analytical quality control [22]. For instance, multiple samples and multiple detections were employed in the flavonoid database. Moreover, the flavonoid amounts by cultivars of each food/beverage were well documented in the flavonoid database [22]. Furthermore, the retention factors were introduced to assess the loss of flavonoids during cooking, as described in the materials and methods section.

In addition, the effects of seasonal variation on amounts of flavonoids in plants or on total flavonoid intake may be not as significant as other factors [56,57]. It is reported that only blueberries demonstrated a seasonality [22]. Since most foods/beverages are available the whole year for consumers, the seasonal variation might be due to the different cultivars or storage methods [22].

Considering that an individual’s dietary structure may change over a lifetime, we compared changes in flavonoid intake across a study population spanning ten years. We found that the flavonoid consumption remained relatively stable, although there were significant differences in the intake of some flavonoids. We employed the mean of the two-day intake of each flavonoid as well as the weights “wtdr2d” constructed for participants who completed two days of dietary recall in making estimates representative of the US non-hospitalized population. Our results could reflect the change in flavonoid consumptions in population. Our study had several limitations. The observational analysis only revealed an association (rather than causality). Notably, dietary flavonoid intake did not include the intake of flavonoid supplements, contributing to the limitations of our results. As discussed above, there are possible health risks associated with excess flavonoid intake. Future studies should include dietary and supplemental flavonoid intakes.

## 5. Conclusions

In summary, in comparison to being in the first quartile of dietary flavonol intake, being in the second, third, and fourth quartile was inversely associated with cancer-related mortality. Potential protective effects of dietary flavonol intake against cancer death was observed, especially in participants aged 50 or above, males, whites, former smokers, people who used to drink, mild drinkers, people without hyperlipidemia, and people with hypertension. Total dietary intakes of peonidin, naringenin, and catechin were inversely associated with the mortality of cancer. The nomogram based on the dietary intake of flavonols was clinically applicable to estimating the possibility of cancer-related death. Regarding the perspective of public health, our results may provide new insight into assessing cancer mortality risk based on dietary flavonol intake and establishing future personalized dietary recommendations amongst people with unhealthy lifestyles. However, further evidence from randomized controlled trials is still needed to assess the health benefits of flavonoids.

## Figures and Tables

**Table 1:** Statistical description of flavonoid intakes.

Intake of Flavonoids (mg/day)	Minimum	25th Percentile	Median	Mean	75th Percentile	Maximum
Daidzein	0	0	0	0.6919	0.025	151
Genistein	0	0	0.01	0.9426	0.045	204.32
Glycitein	0	0	0	0.1341	0	35.28
Cyanidin	0	0.045	0.515	2.447	1.885	639.96
Petunidin	0	0	0	0.9845	0.37	144.4
Delphinidin	0	0	0.01	1.4	0.66	187.14
Malvidin	0	0	0	4.258	1.81	309.485
Pelargonidin	0	0	0.01	1.427	0.35	91.135
Peonidin	0	0	0.095	1.705	0.66	636.765
Catechin	0	1.825	5.025	7.224	9.8	166.845
Epigallocatechin	0	0.105	0.485	14.408	13.825	1083.675
Epicatechin	0	1.375	5.68	9.232	12.71	316.94
Epicatechin 3-gallate	0	0	0.02	9.294	8.895	666.03
Epigallocatechin 3-gallate	0	0	0.14	24.98	21.93	2606.33
Theaflavin	0	0	0	1.354	0.845	75.87
Thearubigins	0	0	0	78.11	73.62	3891
Eriodictyol	0	0	0	0.196	0.095	47
Hesperetin	0	0	0.135	9.818	12.695	509.42
Naringenin	0	0.035	0.305	3.708	3.49	224.35
Apigenin	0	0.015	0.065	0.1953	0.21	70.01
Luteolin	0	0.115	0.355	0.6633	0.85	43.305
Isorhamnetin	0	0.13	0.46	0.8339	1.045	75.155
Kaempferol	0	0.895	2.325	4.207	5.485	152.885
Myricetin	0	0.245	0.6	1.373	1.625	39.645
Quercetin	0	4.32	8.09	10.65	13.9	202.75
Theaflavin-3,3’-digallate	0	0	0	1.493	0.94	83.66
Theaflavin-3’-gallate	0	0	0	1.264	0.605	72.18
Theaflavin-3-gallate	0	0	0	1.074	0.74	59.67
Gallocatechin	0	0	0.02	1.466	1.42	73.66
Subtotal Catechins	0	4.78	14.49	66.6	63.83	4897.1
Total Isoflavones	0	0	0.01	1.768	0.08	390.6
Total Anthocyanidins	0	0.11	2.02	12.22	10.78	756.1
Total Flavan-3-ols	0	4.915	15.425	149.891	154.295	6724.88
Total Flavanones	0	0.055	0.61	13.722	18.925	590.625
Total Flavones	0	0.18	0.505	0.8586	1.085	87.245
Total Flavonols	0	6.815	12.555	17.064	22.105	332.035
Total Sum of all 29 flavonoids	0	24.31	64.05	195.53	217.38	6974.47

**Table 2:** Characteristics of participant by living status, NHANES 2007-2010, 2017-2018 until 31 December 2019.

Variable	Alive (*n* = 13,624)	Death Caused by Cancer (*n* = 405)	*p* Value
Baseline sociodemographic, lifestyle, and health-related variables			
Age, years	46.67 ± 0.32	65.93 ± 0.89	<0.0001
Gender, %			0.01
Female	52.86% (51.76%, 53.96%)	42.70% (36.11%, 49.29%)	
Male	47.14% (46.04%, 48.24%)	57.30% (50.71%, 63.89%)	
Ethnicity, %			0.002
Black	11.41% (9.61%, 13.21%)	12.15% (8.61%, 15.69%)	
Mexican	8.74% (6.73%, 10.74%)	3.32% (1.03%, 5.60%)	
Other	13.14% (11.21%, 15.08%)	6.82% (2.37%, 11.27%)	
White	66.71% (63.01%, 70.40%)	77.71% (70.94%, 84.48%)	
Education, %			<0.0001
<9 years	4.89% (4.16%, 5.61%)	10.18% (6.45%, 13.92%)	
9−12 years	35.37% (33.30%, 37.44%)	45.38% (39.66%, 51.10%)	
>12 years	59.74% (57.48%, 62.00%)	44.44% (37.76%, 51.11%)	
Marital status, %			0.67
Without partner	37.08% (35.23%, 38.93%)	38.69% (31.22%, 46.15%)	
With partner	62.92% (61.07%, 64.77%)	61.31% (53.85%, 68.78%)	
PIR	3.02 ± 0.04	2.65 ± 0.15	0.02
BMI (kg/m^2^)	29.14 ± 0.13	29.04 ± 0.52	0.84
Daily energy intake (kcal)	4161.93 ± 27.93	3828.29 ± 112.64	0.01
Total score of HEI	53.25 ± 0.36	54.40 ± 0.97	0.23
DII	1.47 ± 0.05	1.77 ± 0.12	0.02
Total time of PA (mins/week)	1288.52 ± 29.66	1244.04 ± 166.92	0.79
Total MET of PA (/week)	5124.83 ± 137.52	4375.36 ± 681.03	0.28
Smoking status, %			<0.0001
Never	56.97% (54.88%, 59.05%)	35.95% (27.82%, 44.09%)	
Former	24.20% (22.87%, 25.53%)	36.55% (28.37%, 44.73%)	
Now	18.8%3 (17.43%, 20.23%)	27.50% (19.33%, 35.66%)	
Alcohol consumption, %			<0.0001
Never	10.72% (9.76%, 11.68%)	9.02% (4.87%, 13.17%)	
Former	10.65% (9.35%, 11.94%)	36.13% (28.06%, 44.20%)	
Mild	38.38% (36.43%, 40.33%)	31.86% (23.67%, 40.06%)	
Moderate	18.17% (17.08%, 19.26%)	12.68% (8.19%, 17.17%)	
Heavy	22.08% (20.72%, 23.44%)	10.31% (4.79%, 15.84%)	
Dietary intake of flavonoids (mg/day)			
Daidzein	0.80 ± 0.06	0.37 ± 0.12	<0.001
Genistein	1.13 ± 0.08	0.46 ± 0.15	<0.001
Glycitein	0.16 ± 0.01	0.06 ± 0.02	<0.001
Cyanidin	2.69 ± 0.19	2.17 ± 0.32	0.2
Petunidin	1.20 ± 0.09	0.83 ± 0.16	0.03
Delphinidin	1.72 ± 0.14	1.22 ± 0.21	0.03
Malvidin	4.92 ± 0.31	3.59 ± 0.60	0.04
Pelargonidin	1.64 ± 0.12	1.53 ± 0.36	0.72
Peonidin	2.12 ± 0.16	0.87 ± 0.21	<0.0001
Catechin	7.83 ± 0.18	6.95 ± 0.50	0.07
Epigallocatechin	16.76 ± 0.81	13.72 ± 1.89	0.13
Epicatechin	10.13 ± 0.24	8.73 ± 0.66	0.04
Epicatechin 3-gallate	10.81 ± 0.53	8.88 ± 1.28	0.15
Epigallocatechin 3-gallate	28.90 ± 1.65	23.03 ± 3.19	0.09
Theaflavin	1.59 ± 0.08	1.39 ± 0.22	0.39
Thearubigins	90.86 ± 4.36	79.31 ± 11.27	0.32
Eriodictyol	0.17 ± 0.01	0.14 ± 0.02	0.12
Hesperetin	8.86 ± 0.26	9.77 ± 1.08	0.42
Naringenin	3.40 ± 0.16	2.62 ± 0.36	0.04
Apigenin	0.24 ± 0.03	0.21 ± 0.02	0.34
Luteolin	0.71 ± 0.02	0.59 ± 0.05	0.02
Isorhamnetin	0.86 ± 0.02	0.77 ± 0.07	0.17
Kaempferol	4.67 ± 0.10	3.59 ± 0.33	0.002
Myricetin	1.54 ± 0.04	1.31 ± 0.11	0.04
Quercetin	11.38 ± 0.19	10.31 ± 0.65	0.09
Theaflavin-3,3′-digallate	1.75 ± 0.09	1.54 ± 0.24	0.39
Theaflavin-3′-gallate	1.48 ± 0.08	1.30 ± 0.21	0.4
Theaflavin-3-gallate	1.26 ± 0.06	1.11 ± 0.17	0.41
Gallocatechin	1.71 ± 0.07	1.43 ± 0.22	0.21
Subtotal Catechins	76.14 ± 3.36	62.75 ± 7.34	0.09
Total Isoflavones	2.09 ± 0.15	0.89 ± 0.29	<0.001
Total Anthocyanidins	14.30 ± 0.74	10.21 ± 1.40	0.01
Total Flavan-3-ols	173.08 ± 7.19	147.41 ± 19.38	0.2
Total Flavanones	12.43 ± 0.39	12.53 ± 1.34	0.95
Total Flavones	0.95 ± 0.04	0.80 ± 0.06	0.03
Total Flavonols	18.45 ± 0.32	15.97 ± 1.07	0.02
Total Sum of all 29 flavonoids	221.30 ± 7.48	187.80 ± 20.34	0.11
Disease history at interview			
DM, %			<0.0001
No	79.30% (77.90%, 80.70%)	61.21% (54.12%, 68.31%)	
Yes	20.70% (19.30%, 22.10%)	38.79% (31.69%, 45.88%)	
Hyperlipidemia, %			0.12
No	31.43% (29.70%, 33.17%)	25.08% (17.66%, 32.50%)	
Yes	68.57% (66.83%, 70.30%)	74.92% (67.50%, 82.34%)	
CVD, %			<0.0001
No	91.48% (90.62%, 92.34%)	65.84% (59.01%, 72.66%)	
Yes	8.52% (7.66%, 9.38%)	34.16% (27.34%, 40.99%)	
Respiratory system disease, %			<0.0001
ACO	2.06% (1.70%, 2.42%)	8.06% (1.88%, 14.23%)	
Asthma	11.70% (10.75%, 12.64%)	7.62% (4.20%, 11.03%)	
COPD	2.83% (2.33%, 3.32%)	15.46% (9.30%, 21.63%)	
No	83.42% (82.16%, 84.68%)	68.86% (61.16%, 76.56%)	
Stroke, %			<0.0001
No	96.86% (96.50%, 97.21%)	84.35% (78.74%, 89.97%)	
Yes	3.14% (2.79%, 3.50%)	15.65% (10.03%, 21.26%)	
Cancer, %			<0.001
No	90.33% (89.67%, 90.98%)	80.53% (74.37%, 86.68%)	
Yes	9.67% (9.02%, 10.33%)	19.47% (13.32%, 25.63%)	
Hypertension, %			<0.0001
No	63.80% (62.09%, 65.51%)	26.20% (19.40%, 32.99%)	
Yes	36.20% (34.49%, 37.91%)	73.80% (67.01%, 80.60%)	

Continuous variables are presented as mean ± standard deviation and categorical variables are presented as percentage (95% CIs).

**Table 3:** Characteristics of participant by survey year cycle 2007–2008, 2009–2010, and 2017–2018.

	2007–2008	2009–2010	2017–2018	*p*-Value
Dietary intake of flavonoids (mg/day)				
Daidzein	0.62 ± 0.08	0.79 ± 0.05	0.93 ± 0.13	0.09
Genistein	0.84 ± 0.10	1.12 ± 0.07	1.36 ± 0.19	0.02
Glycitein	0.11 ± 0.01	0.16 ± 0.01	0.21 ± 0.03	0.01
Cyanidin	2.22 ± 0.19	2.99 ± 0.25	2.82 ± 0.42	0.04
Petunidin	0.77 ± 0.10	1.38 ± 0.18	1.40 ± 0.17	0.001
Delphinidin	1.04 ± 0.13	2.10 ± 0.29	1.96 ± 0.24	<0.001
Malvidin	4.05 ± 0.46	5.23 ± 0.47	5.36 ± 0.56	0.11
Pelargonidin	1.44 ± 0.21	1.88 ± 0.25	1.60 ± 0.16	0.36
Peonidin	1.23 ± 0.12	2.11 ± 0.30	2.85 ± 0.32	<0.0001
Catechin	7.71 ± 0.32	8.18 ± 0.24	7.58 ± 0.33	0.26
Epigallocatechin	17.41 ± 1.11	15.93 ± 1.30	16.76 ± 1.57	0.66
Epicatechin	10.24 ± 0.41	10.13 ± 0.37	9.97 ± 0.41	0.9
Epicatechin 3-gallate	11.50 ± 0.74	10.44 ± 0.88	10.41 ± 0.98	0.53
Epigallocatechin 3-gallate	30.12 ± 1.90	27.50 ± 2.25	28.75 ± 3.60	0.64
Theaflavin	1.77 ± 0.12	1.59 ± 0.14	1.41 ± 0.15	0.22
Thearubigins	101.89 ± 6.79	91.63 ± 8.00	79.87 ± 7.67	0.14
Eriodictyol	0.19 ± 0.01	0.20 ± 0.01	0.14 ± 0.01	0.004
Hesperetin	9.55 ± 0.61	10.05 ± 0.35	7.24 ± 0.35	<0.0001
Naringenin	3.52 ± 0.29	3.40 ± 0.20	3.26 ± 0.30	0.82
Apigenin	0.23 ± 0.03	0.31 ± 0.07	0.17 ± 0.01	0.03
Luteolin	0.64 ± 0.04	0.74 ± 0.03	0.74 ± 0.04	0.11
Isorhamnetin	0.80 ± 0.04	0.90 ± 0.03	0.87 ± 0.03	0.08
Kaempferol	4.55 ± 0.19	4.64 ± 0.16	4.74 ± 0.12	0.7
Myricetin	1.51 ± 0.07	1.46 ± 0.08	1.64 ± 0.07	0.23
Quercetin	11.70 ± 0.41	11.91 ± 0.30	10.58 ± 0.24	0.004
Theaflavin-3,3′-digallate	1.96 ± 0.14	1.76 ± 0.16	1.55 ± 0.17	0.2
Theaflavin-3′-gallate	1.66 ± 0.12	1.49 ± 0.13	1.32 ± 0.14	0.23
Theaflavin-3-gallate	1.42 ± 0.10	1.27 ± 0.12	1.10 ± 0.12	0.17
Gallocatechin	1.87 ± 0.12	1.71 ± 0.14	1.55 ± 0.12	0.23
Subtotal Catechins	78.85 ± 4.38	73.90 ± 5.11	75.03 ± 6.76	0.72
Total Isoflavones	1.58 ± 0.19	2.06 ± 0.13	2.50 ± 0.34	0.04
Total Anthocyanidins	10.74 ± 0.91	15.68 ± 1.09	15.99 ± 1.48	<0.001
Total Flavan-3-ols	187.55 ± 11.60	171.63 ± 13.66	160.28 ± 11.43	0.27
Total Flavanones	13.26 ± 0.87	13.65 ± 0.52	10.64 ± 0.58	0.001
Total Flavones	0.87 ± 0.05	1.05 ± 0.08	0.91 ± 0.04	0.18
Total Flavonols	18.56 ± 0.68	18.92 ± 0.52	17.82 ± 0.40	0.26
Total Sum of all 29 flavonoids	232.55 ± 12.43	222.98 ± 14.09	208.13 ± 11.66	0.39
Baseline sociodemographic, lifestyle, and health-related variables				
Age, years	46.85 ± 0.47	46.96 ± 0.51	47.29 ± 0.55	0.82
Gender, %				0.41
Female	53.69% (51.99%, 55.40%)	52.22% (51.03%, 53.40%)	52.15% (49.83%, 54.47%)	
Male	46.31% (44.60%, 48.01%)	47.78% (46.60%, 48.97%)	47.85% (45.53%, 50.17%)	
Ethnicity, %				0.16
Black	11.15% (7.55%, 14.75%)	11.49% (9.76%, 13.23%)	11.61% (8.35%, 14.87%)	
Mexican	8.39% (5.63%, 11.16%)	8.32% (4.51%, 12.13%)	9.12% (5.80%, 12.44%)	
Other	9.91% (6.71%, 13.10%)	11.56% (8.05%, 15.08%)	17.06% (14.12%, 19.99%)	
White	70.55% (64.29%, 76.80%)	68.62% (62.27%, 74.97%)	62.22% (56.62%, 67.82%)	
Education, %				0.002
<9 years	6.29% (4.87%, 7.71%)	5.84% (4.53%, 7.15%)	3.03% (2.14%, 3.92%)	
9–12 years	38.37% (34.35%, 42.38%)	34.87% (32.08%, 37.67%)	33.65% (30.25%, 37.04%)	
>12 years	55.34% (50.57%, 60.11%)	59.29% (56.52%, 62.05%)	63.32% (59.82%, 66.83%)	
Marital status, %				0.81
Without partner	37.71% (33.75%, 41.68%)	36.42% (34.30%, 38.54%)	37.20% (34.42%, 39.98%)	
With partner	62.29% (58.32%, 66.25%)	63.58% (61.46%, 65.70%)	62.80% (60.02%, 65.58%)	
PIR	3.01 ± 0.09	2.96 ± 0.05	3.06 ± 0.06	0.47
BMI (kg/m2)	28.74 ± 0.20	28.83 ± 0.13	29.77 ± 0.28	0.01
Daily energy intake (kcal)	4120.26 ± 52.88	4207.67 ± 44.41	4140.31 ± 43.02	0.36
Total Score of HEI	53.09 ± 0.65	54.30 ± 0.36	52.52 ± 0.71	0.04
DII	1.65 ± 0.10	1.33 ± 0.04	1.45 ± 0.09	0.004
Total time of PA (mins/week)	1274.92 ± 50.84	1102.61 ± 33.04	1463.02 ± 60.66	<0.0001
Total MET of PA (/week)	5094.71 ± 230.20	4243.83 ± 171.63	5870.06 ± 274.40	<0.0001
Smoking status, %				0.01
Never	53.62% (50.24%, 57.00%)	55.61% (51.57%, 59.66%)	59.99% (56.97%, 63.01%)	
Former	24.11% (22.33%, 25.89%)	25.11% (22.18%, 28.04%)	24.13% (22.21%, 26.05%)	
Now	22.27% (19.75%, 24.79%)	19.27% (17.31%, 21.24%)	15.88% (13.51%, 18.25%)	
Alcohol usage, %				<0.0001
Never	11.56% (9.94%, 13.17%)	10.87% (9.09%, 12.65%)	9.62% (8.28%, 10.96%)	
Former	17.53% (14.56, 20.50)	15.21% (13.27%, 17.15%)	0.65% (0.33%, 0.98%)	
Mild	34.23% (30.60, 37.85)	35.69% (32.90%, 38.47%)	44.89% (41.47%, 48.31%)	
Moderate	15.60% (13.66, 17.54)	16.65% (14.58%, 18.72%)	21.97% (20.55%, 23.39%)	
Heavy	21.09% (19.46, 22.71)	21.59% (19.12%, 24.05%)	22.87% (20.11%, 25.63%)	
Disease history at interview				
DM, %				0.45
No	78.14% (75.37%, 80.90%)	80.15% (78.17%, 82.13%)	78.61% (76.44%, 80.79%)	
Yes	21.86% (19.10%, 24.63%)	19.85% (17.87%, 21.83%)	21.39% (19.21%, 23.56%)	
Hyperlipidemia, %				0.002
No	28.37% (25.98%, 30.76%)	29.88% (28.12%, 31.64%)	35.17% (31.50%, 38.83%)	
Yes	71.63% (69.24%, 74.02%)	70.12% (68.36%, 71.88%)	64.83% (61.17%, 68.50%)	
CVD, %				0.96
No	91.06% (90.00%, 92.12%)	91.06% (89.71%, 92.41%)	90.84% (89.16%, 92.53%)	
Yes	8.94% (7.88%, 10.00%)	8.94% (7.59%, 10.29%)	9.16% (7.47%, 10.84%)	
Respiratory system disease, %				<0.0001
ACO	2.58% (2.05%, 3.11%)	2.11% (1.57%, 2.65%)	1.87% (1.07%, 2.66%)	
Asthma	11.83% (9.90%, 13.75%)	10.14% (9.25%, 11.04%)	12.78% (11.05%, 14.51%)	
COPD	4.39% (3.46%, 5.33%)	3.81% (2.65%, 4.98%)	1.19% (0.80%, 1.58%)	
No	81.20% (78.58%, 83.82%)	83.94% (82.45%, 85.42%)	84.16% (81.87%, 86.44%)	
Stroke, %				0.49
No	96.30% (95.59%, 97.00%)	96.83% (96.33%, 97.34%)	96.70% (95.99%, 97.40%)	
Yes	3.70% (3.00%, 4.41%)	3.17% (2.66%, 3.67%)	3.30% (2.60%, 4.01%)	
Cancer, %				0.46
No	90.69% (89.63%, 91.75%)	90.06% (88.86%, 91.26%)	89.70% (88.56%, 90.83%)	
Yes	9.31% (8.25%, 10.37%)	9.94% (8.74%, 11.14%)	10.30% (9.17%, 11.44%)	
Hypertension, %				0.18
No	63.63% (61.50%, 65.76%)	64.67% (61.79%, 67.55%)	61.19% (58.00%, 64.38%)	
Yes	36.37% (34.24%, 38.50%)	35.33% (32.45%, 38.21%)	38.81% (35.62%, 42.00%)	

Continuous variables are presented as mean ± standard deviation and categorical variables are presented as percentage (95% CIs).

**Table 4:** Mean dietary intake of flavonoid subclass by age, gender, and ethnic group.

Age and Gender Group	Case, n	Subtotal Catechins (mg/day)	Total Isoflavones(mg/day)	Total Anthocyanidins (mg/day)	Total Flavan-3-ols (mg/day)	Total Flavanones (mg/day)	Total Flavones (mg/day)	Total Flavonols (mg/day)	Total Sum of All 29 Flavonoids (mg/day)
White									
All	6361	84.12 (74.74, 93.50)	2.05 (1.66, 2.45)	16.12 (13.98, 18.25)	195.74 (176.20, 215.28)	11.21 (10.38, 12.04)	0.99 (0.89, 1.09)	19.45 (18.51, 20.39)	245.56 (225.09, 266.04)
male < 50	1299	82.42 (66.25, 98.60)	2.56 (1.94, 3.19)	11.28 (8.22, 14.34)	186.79 (154.01, 219.57)	11.03 (9.01, 13.06)	1.02 (0.78, 1.26)	20.55 (18.87, 22.23)	233.23 (199.48, 266.98)
female < 50	1458	76.74 (63.04, 90.44)	1.91 (1.13, 2.68)	14.93 (12.46, 17.40)	179.59 (151.12, 208.05)	7.68 (6.53, 8.83)	0.82 (0.72, 0.91)	17.06 (15.66, 18.45)	221.98 (191.88, 252.07)
male ≧ 50	1825	82.98 (71.91, 94.04)	1.77 (0.97, 2.56)	18.09 (14.71, 21.47)	200.97 (171.61, 230.34)	14.05 (12.33, 15.77)	1.10 (0.95, 1.25)	20.89 (19.63, 22.15)	256.88 (226.01, 287.74)
female ≧ 50	1779	94.01 (71.62, 116.40)	1.98 (1.42, 2.53)	20.05 (17.08, 23.01)	215.48 (184.48, 246.49)	12.41 (10.83, 13.99)	1.05 (0.82, 1.27)	19.54 (18.15, 20.94)	270.51 (238.49, 302.52)
Black									
All	2904	51.92 (46.67, 57.17)	1.43 (1.00, 1.86)	8.27 (7.07, 9.48)	118.85 (105.57, 132.13)	14.17 (12.79, 15.54)	0.60 (0.56, 0.64)	14.53 (13.83, 15.23)	157.85 (144.43, 171.26)
male < 50	618	52.30 (44.65, 59.94) ***	2.24 (0.79, 3.69)	8.16 (6.15, 10.17) ***	118.94 (100.24, 137.64) ***	15.47 (12.65, 18.30) ***	0.54 (0.49, 0.60) ***	15.78 (14.40, 17.17) ***	161.14 (140.57, 181.70) ***
female < 50	767	46.39 (38.01, 54.77) ***	1.14 (0.52, 1.76) *	7.40 (6.21, 8.58) ***	102.25 (80.69, 123.82) ***	14.12 (11.87, 16.37) ***	0.61 (0.52, 0.69) ***	12.78 (11.78, 13.78) ***	138.30 (115.47, 161.12) ***
male ≧ 50	745	56.95 (44.95, 68.96) ***	0.98 (0.51, 1.44)	8.19 (6.37, 10.00) ***	140.29 (107.30, 173.28) ***	14.44 (12.08, 16.80)	0.63 (0.54, 0.71) ***	16.81 (15.31, 18.32) ***	181.34 (147.20, 215.48) ***
female ≧ 50	774	56.46 (47.39, 65.54) ***	1.19 (0.53, 1.85) ***	9.92 (6.79, 13.05) ***	128.77 (112.63, 144.91) ***	12.30 (10.07, 14.53) *	0.64 (0.57, 0.72) ***	13.91 (12.84, 14.99) ***	166.74 (148.28, 185.20) ***
Mexican									
All	2222	45.48 (38.26, 52.70)	2.23 (0.83, 3.63)	8.60 (6.74, 10.47)	89.90 (72.66, 107.13)	16.16 (14.43, 17.89)	0.97 (0.88, 1.06)	15.41 (14.55, 16.27)	133.27 (115.85, 150.69)
male < 50	596	48.62 (40.34, 56.90) ***	1.97 (0.30, 3.64)	7.18 (4.50, 9.87) ***	85.23 (67.11, 103.35) ***	17.85 (15.02, 20.68) ***	0.98 (0.86, 1.11)	16.94 (15.57, 18.32) ***	130.16 (110.49, 149.84) ***
female < 50	681	41.91 (31.21, 52.61) ***	3.47 (−0.12, 7.06)	8.01 (6.01, 10.02) ***	89.48 (65.06, 113.89) ***	14.05 (11.88, 16.23) ***	0.90 (0.76, 1.05)	13.93 (12.63, 15.24) ***	129.85 (104.53, 155.16) **
male ≧ 50	442	48.55 (30.97, 66.12) ***	0.96 (0.37, 1.54)	10.73 (5.95, 15.52) ***	102.11 (57.99, 146.24) ***	18.10 (14.21, 22.00) ***	1.18 (0.62, 1.74)	16.92 (14.44, 19.41) ***	150.01 (104.08, 195.94) ***
female ≧ 50	503	43.60 (35.53, 51.67) ***	0.83 (0.45, 1.22)	12.13 (5.69, 18.56) ***	92.77 (71.66, 113.88) ***	15.39 (12.96, 17.81) ***	0.92 (0.81, 1.03)	13.74 (12.32, 15.16) ***	135.78 (114.28, 157.28) ***
Other									
All	2542	74.72 (61.57, 87.88)	2.58 (2.02, 3.13)	13.42 (10.99, 15.84)	155.55 (133.35, 177.75)	14.73 (12.86, 16.60)	0.97 (0.90, 1.05)	18.45 (17.40, 19.50)	205.69 (182.44, 228.95)
male < 50	617	65.64 (35.89, 95.39)	2.64 (1.80, 3.48)	11.23 (7.18, 15.27)	129.79 (90.52, 169.05) **	16.52 (13.11, 19.93) ***	0.96 (0.84, 1.08)	19.01 (17.12, 20.89)	180.14 (138.41, 221.88) **
female < 50	744	55.10 (45.61, 64.59)	2.53 (1.55, 3.51)	13.05 (10.26, 15.84)	121.23 (97.05, 145.41) **	13.90 (11.47, 16.33) ***	0.90 (0.77, 1.03)	16.11 (14.66, 17.56)	167.73 (141.56, 193.89) **
male ≧ 50	555	99.23 (73.52, 124.94)	2.48 (1.43, 3.52)	16.35 (10.81, 21.89)	213.04 (160.48, 265.60)	14.26 (11.18, 17.33) *	1.15 (0.98, 1.32)	21.66 (19.33, 23.99)	268.93 (215.27, 322.59)
female ≧ 50	626	104.70 (76.67, 132.72)	2.62 (1.68, 3.56)	15.43 (11.42, 19.45)	213.06 (167.17, 258.95) *	13.51 (11.05, 15.97) *	0.99 (0.82, 1.15)	18.98 (16.79, 21.18)	264.59 (216.78, 312.40) *

Means (95% CIs) are given from a weighted analysis. *** *p* < 0.001 compared with white participants. ** 0.001 ≦ *p* < 0.01 compared with white participants. * 0.01 ≦ *p* < 0.05 compared with white participants.

**Table 5:** Hazard ratios of cancer mortality by quartiles of dietary flavonoid intake.

	Flavonoid Intake Quartiles								
	1Q	2Q	*p* for 1Q vs. 2Q	3Q	*p* for 1Q vs. 3Q	4Q	*p* for 1Q vs. 4Q	HR (95%CI)	*p* for Trend
Total flavonoid (mg/day)	≦24.31	24.31–64.05		64.05–217.38		>217.38			
Model 1 (unadjusted)	1	1.15 (0.71, 1.88)	0.57	0.76 (0.47, 1.22)	0.26	0.74 (0.49, 1.11)	0.14	0.88 (0.78, 0.99)	0.03
Model 2 (adjusted for age, ethnicity, and gender)	1	0.97 (0.61, 1.55)	0.91	0.54 (0.33, 0.89)	0.02	0.60 (0.40, 0.91)	0.03	0.81 (0.71, 0.92)	<0.01
Model 3 (multivariate)	1	1.16 (0.74, 1.81)	0.52	0.73 (0.45, 1.17)	0.19	0.76 (0.48, 1.20)	0.24	0.88 (0.76, 1.02)	0.10
Total flavones (mg/day)	≦0.18	0.18–0.51		0.51–1.09		>1.09			
Model 1 (unadjusted)	1	0.52 (0.33, 0.82)	0.01	0.67 (0.45, 0.97)	0.04	0.79 (0.52, 1.21)	0.28	0.95 (0.81, 1.11)	0.52
Model 2 (adjusted for age, ethnicity, and gender)	1	0.46 (0.28, 0.74)	0.001	0.58 (0.39, 0.86)	0.01	0.69 (0.46, 1.05)	0.08	0.91 (0.78, 1.07)	0.26
Model 3 (multivariate)	1	0.48 (0.26, 0.87)	0.02	0.72 (0.47, 1.10)	0.12	1.02 (0.62, 1.67)	0.94	1.04 (0.88, 1.24)	0.63
Total anthocyanidins (mg/day)	≦0.11	0.11–2.02		2.02–10.78		>10.78			
Model 1 (unadjusted)	1	0.89 (0.57, 1.39)	0.60	0.91 (0.60, 1.37)	0.64	0.72 (0.44, 1.19)	0.20	0.91 (0.78, 1.06)	0.22
Model 2 (adjusted for age, ethnicity, and gender)	1	0.74 (0.47, 1.18)	0.21	0.65 (0.42, 1.02)	0.06	0.46 (0.27, 0.79)	<0.01	0.78 (0.66, 0.92)	<0.01
Model 3 (multivariate)	1	0.86 (0.49, 1.50)	0.60	0.82 (0.48, 1.39)	0.46	0.63 (0.32, 1.23)	0.18	0.87 (0.71, 1.06)	0.17
Total flavanones (mg/day)	≦0.06	0.06–0.61		0.61–18.93		>18.93			
Model 1 (unadjusted)	1	0.90 (0.59, 1.36)	0.61	0.79 (0.50, 1.25)	0.319	1.01 (0.68, 1.50)	0.97	0.99 (0.86, 1.14)	0.90
Model 2 (adjusted for age, ethnicity, and gender)	1	0.89 (0.58, 1.36)	0.58	0.72 (0.46, 1.14)	0.16	0.74 (0.48, 1.12)	0.16	0.89 (0.78, 1.03)	0.13
Model 3 (multivariate)	1	1.23 (0.73, 2.07)	0.44	1.06 (0.66, 1.73)	0.80	1.02 (0.65, 1.59)	0.93	0.99 (0.86, 1.13)	0.88
Total flavonol (mg/day)	≦6.82	6.82–12.56		12.56–22.11		>22.11			
Model 1 (unadjusted)	1	0.60 (0.41, 0.88)	0.01	0.63 (0.39, 1.05)	0.08	0.53 (0.34, 0.83)	0.01	0.83 (0.70, 0.97)	0.02
Model 2 (adjusted for age, ethnicity, and gender)	1	0.56 (0.39, 0.79)	0.001	0.59 (0.37, 0.93)	0.03	0.51 (0.33, 0.81)	<0.01	0.82 (0.69, 0.96)	0.02
Model 3 (multivariate)	1	0.58 (0.36, 0.91)	0.02	0.55 (0.31, 0.96)	0.04	0.54 (0.30, 0.99)	0.05	0.82 (0.67, 1.02)	0.08
Total Flavan–3–ols (mg/day)	≦4.92	4.92–15.43		15.43–154.30		>154.30			
Model 1 (unadjusted)	1	0.88 (0.60, 1.30)	0.53	0.90 (0.56, 1.46)	0.68	0.71 (0.47, 1.08)	0.11	0.90 (0.79, 1.03)	0.13
Model 2 (adjusted for age, ethnicity, and gender)	1	0.71 (0.48, 1.05)	0.09	0.66 (0.42, 1.03)	0.07	0.57 (0.38, 0.86)	0.01	0.84 (0.73, 0.96)	0.01
Model 3 (multivariate)	1	0.82 (0.53, 1.27)	0.38	0.79 (0.48, 1.31)	0.36	0.68 (0.42, 1.12)	0.13	0.89 (0.76, 1.04)	0.15
Subtotal Catechins (mg/day)	≦4.78	4.78–14.49		14.49–63.83		>63.83			
Model 1 (unadjusted)	1	0.89 (0.60, 1.32)	0.55	0.83 (0.51, 1.36)	0.46	0.74 (0.49, 1.11)	0.14	0.91 (0.80, 1.03)	0.14
Model 2 (adjusted for age, ethnicity, and gender)	1	0.71 (0.47, 1.07)	0.10	0.61 (0.39, 0.97)	0.04	0.59 (0.39, 0.88)	0.011	0.84 (0.74, 0.96)	0.01
Model 3 (multivariate)	1	0.81 (0.51, 1.28)	0.36	0.72 (0.43, 1.21)	0.21	0.71 (0.44, 1.16)	0.18	0.90 (0.76, 1.06)	0.19
Total isoflavones (mg/day)	≦0.01	>0.01							
Model 1 (unadjusted)	1	0.74 (0.57, 0.97)							0.03
Model 2 (adjusted for age, ethnicity, and gender)	1	0.81 (0.61, 1.09)							0.16
Model 3 (multivariate)	1	0.93 (0.64, 1.35)							0.70

**Table 6:** Hazard ratios of cancer mortality by quartiles of urinary isoflavones and their metabolites.

	Quartile of Isoflavone Metabolites								
HR (95%CI)	1Q	2Q		3Q		4Q		HR (95%CI)	*p* for Trend
Daidzein (ng/mL)	<16.6	16.6–48.8		48.8–195.0		≧195.0			
model 1 (unadjusted)	1	1.31 (0.72, 2.37)	0.38	0.99 (0.53, 1.85)	0.97	1.22 (0.58, 2.55)	0.60	1.03 (0.82, 1.30)	0.56
model 2 (adjusted for age, race, and sex)	1	1.27 (0.69, 2.31)	0.44	0.88 (0.48, 1.61)	0.68	1.22 (0.60, 2.49)	0.59	1.02 (0.81, 1.29)	0.84
model 3 (multivariate)	1	1.41 (0.91, 2.19)	0.12	0.96 (0.53, 1.72)	0.89	1.38 (0.73, 2.62)	0.32	1.06 (0.87, 1.29)	0.78
ODMA (ng/mL)	<0.60	0.60–2.70		2.70–19.15		≧19.15			
model 1 (unadjusted)	1	1.21 (0.68, 2.14)	0.51	0.90 (0.53, 1.52)	0.70	0.78 (0.46, 1.33)	0.36	0.90 (0.77, 1.06)	0.20
model 2 (adjusted for age, race, and sex)	1	0.93 (0.55, 1.58)	0.79	0.62 (0.38, 1.02)	0.06	0.63 (0.38, 1.05)	0.08	0.83 (0.71, 0.98)	0.03
model 3 (multivariate)	1	0.97 (0.49, 1.94)	0.94	0.63 (0.32, 1.22)	0.17	0.66 (0.31, 1.40)	0.27	0.84 (0.66, 1.06)	0.14
Equol (ng/mL)	<2.70	2.70–6.18		6.18–13.70		≧13.70			
model 1 (unadjusted)	1	0.87 (0.47, 1.62)	0.67	1.04 (0.62, 1.76)	0.88	0.95 (0.54, 1.68)	0.87	1.00 (0.83, 1.22)	0.97
model 2 (adjusted for age, race, and sex)	1	0.85 (0.47, 1.54)	0.59	0.81 (0.50, 1.32)	0.40	0.83 (0.49, 1.40)	0.48	0.94 (0.79, 1.13)	0.53
model 3 (multivariate)	1	0.99 (0.54, 1.82)	0.97	0.88 (0.56, 1.38)	0.57	0.95 (0.60, 1.50)	0.82	0.97 (0.84, 1.13)	0.71
Genistein (ng/mL)	<8.35	8.35–24.90		24.90–88.60		≧88.60			
model 1 (unadjusted)	1	1.12 (0.66, 1.90)	0.69	1.23 (0.75, 2.03)	0.41	1.14 (0.57, 2.29)	0.72	1.05 (0.86, 1.28)	0.65
model 2 (adjusted for age, race, and sex)	1	1.15 (0.68, 1.96)	0.60	1.13 (0.69, 1.86)	0.62	1.09 (0.53, 2.24)	0.82	1.02 (0.83, 1.26)	0.84
model 3 (multivariate)	1	1.20 (0.59, 2.40)	0.62	1.20 (0.65, 2.23)	0.55	1.18 (0.61, 2.30)	0.63	1.05 (0.87, 1.27)	0.63

## Data Availability

Data described in the manuscript, code book, and analytic code will be made publicly and freely available without restriction at [https://www.cdc.gov/nchs/nhanes (accessed on 4 July 2022)].

## Supplementary Materials

The following supporting information can be downloaded at: https://www.mdpi.com/article/10.3390/nu15040976/s1, Figure S1: The association between the intakes of flavonol, peonidin, naringenin, and catechin and log_10_ (hazard ratio of cancer mortality) by restricted cubic splines. Supplementary Table T1: The flavonoid supplements consumed by participants in NHANES [58].
