# Supplementary material for: Dietary Flavonoid Intake and Cancer Mortality: A Population-Based Cohort Study
Source: Nutrients. 2023 Feb 15;15(4):976. doi: 10.3390/nu15040976 (PMC9967058; doi:10.3390/nu15040976)
Supplement: Supplementary file 1 [file nutrients-15-00976-s001.zip › nutrients-2135824-supplementary.pdf]

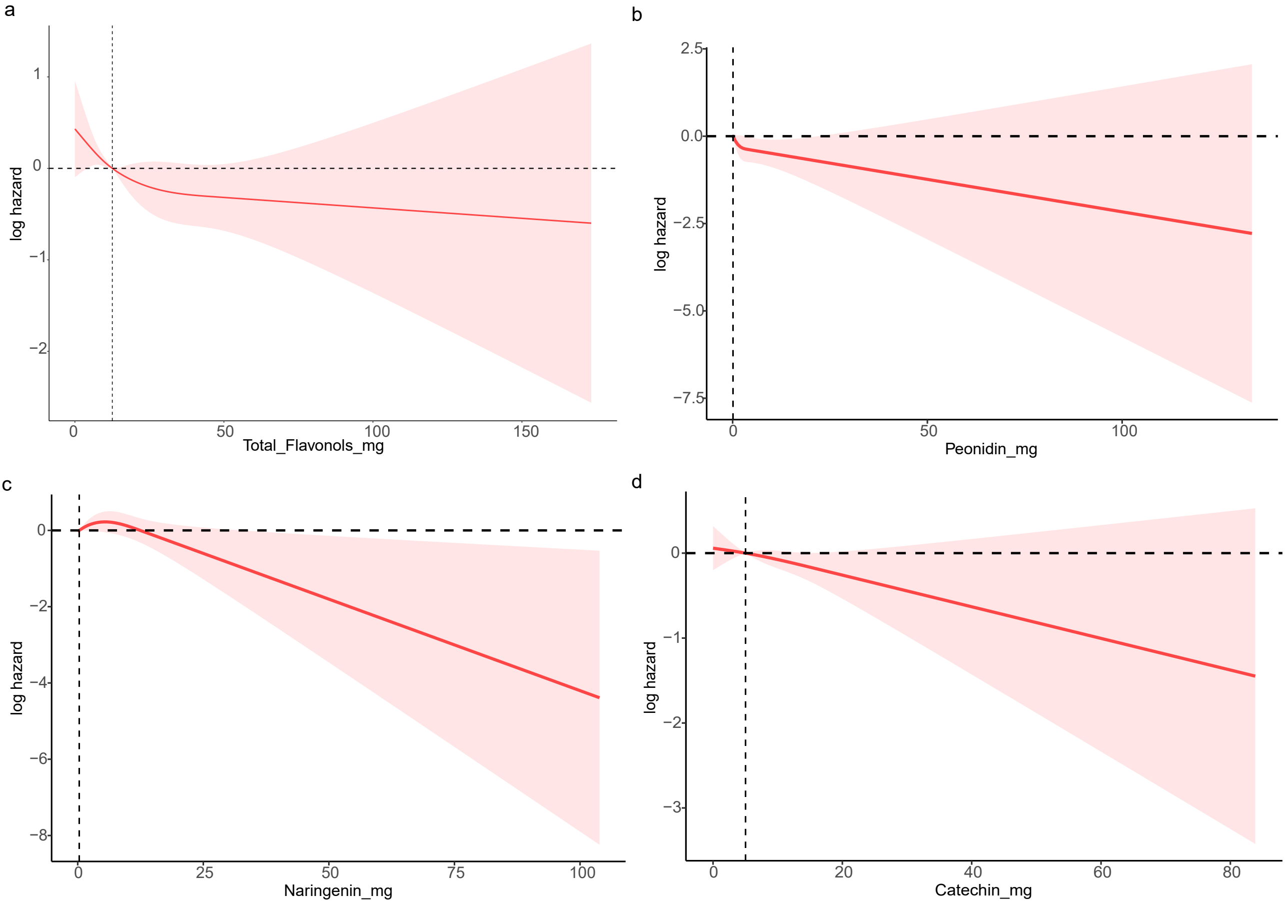

Supplementary Figure S1. The association between intake of flavonol (a), peonidin (b), naringenin (c), and catechin (d) and log $_{10}$ (hazard ratio of cancer mortality) by restricted cubic splines.

Supplementary Table S1 The flavonoid supplements consumed by participants in NHANSE.

supplement_name	supplement_type	ingredient_name	ingredient_category	blend_component_name	blend_component_category
Search for "cyanidin"					
great earth nsp daily nutritional supplement program regular strength	Standard	great earth broad spectrum phyto-antioxidant actives	NA	proanthocyanidins	Other
great earth super oxy e 400 extra strength	Standard	great earth broad spectrum phyto-antioxidant actives	NA	proanthocyanidins	Other
great earth nsp daily nutritional supplement program regular strength	Standard	great earth broad spectrum phyto-antioxidant actives	NA	leucoanthocyanidins	Other
great earth super oxy e 400 extra strength	Standard	great earth broad spectrum phyto-antioxidant actives	NA	leucoanthocyanidins	Other
solgar advanced antioxidant formula	Standard	solgar proanthocyanidin complex blend	NA	pycnogenol	Botanical
solgar advanced antioxidant formula	Standard	solgar proanthocyanidin complex blend	NA	green tea extract	Botanical
solgar advanced antioxidant formula	Standard	solgar proanthocyanidin complex blend	NA	red wine extract	Other
infinity2 nutritionals essentials for life	Standard	essentials for life proprietary vitamin & antioxidant blend	NA	proanthocyanidins	Other
ecoquest / infinity essentials for life 1 all-natural vitamin, mineral and antioxidant complex	Standard	essentials for life 1 proprietary vitamin & antioxidant blend	NA	proanthocyanidins	Other
ol grape seed extract contains oligomeric procyanidins	Standard	olympian labs powerful antioxidant proprietary blend	NA	grape skin extract	Botanical
ol grape seed extract contains oligomeric procyanidins	Standard	olympian labs powerful antioxidant proprietary blend	NA	grape seed extract (95%)	Botanical
immuno stat super nutrient liquid tonic 100% plant sourced pbs	Standard	immuno stat botanical antioxidants	NA	proanthocyanidins	Other
g.f.s.-2000 capsules green food	Standard	douglas laboratories g.f.s.-2000 proanthocyanidins blend	NA	grape seed extract	Botanical
g.f.s.-2000 capsules green food	Standard	douglas laboratories g.f.s.-2000 proanthocyanidins blend	NA	pine bark extract	Botanical
health direct nutrition nature's optimal nutrition a complete, balanced and high-potency multivitamin, antioxidant	Standard	health direct nature's optimal nutrition proprietary botanical antioxidant blend	NA	proanthocyanidins	Other
energique nutrition	Standar	energique	NA	proanthocyanidin	Other

enervimin stress focus	d	nutrition enervimin stress focus proprietary blend		s	
nature's plus hema-plex with 85 mg of elemental iron sustained release vegetarian	Standar d	nature's plus hema-plex proanthoplex proprietary proanthocyanidin / bioflavonoid complex	NA	bioflavonoids	Other
nature's plus hema-plex with 85 mg of elemental iron sustained release vegetarian	Standar d	nature's plus hema-plex proanthoplex proprietary proanthocyanidin / bioflavonoid complex	NA	green tea leaves	Botanical
nature's plus hema-plex with 85 mg of elemental iron sustained release vegetarian	Standar d	nature's plus hema-plex proanthoplex proprietary proanthocyanidin / bioflavonoid complex	NA	bilberry fruit	Botanical
nature's plus hema-plex with 85 mg of elemental iron sustained release vegetarian	Standar d	nature's plus hema-plex proanthoplex proprietary proanthocyanidin / bioflavonoid complex	NA	red raspberry (fruit)	Botanical
nature's plus hema-plex with 85 mg of elemental iron sustained release vegetarian	Standar d	nature's plus hema-plex proanthoplex proprietary proanthocyanidin / bioflavonoid complex	NA	black raspberry (fruit)	Botanical
nature's plus hema-plex with 85 mg of elemental iron sustained release vegetarian	Standar d	nature's plus hema-plex proanthoplex proprietary proanthocyanidin / bioflavonoid complex	NA	blackberry (fruit)	Botanical
dr. mercola whole-food multivitamin plus vital minerals	Standar d	dr. mercola whole-food multivitamin proanthocyanidin blend	NA	grape seed extract	Botanical
dr. mercola whole-food multivitamin plus vital minerals	Standar d	dr. mercola whole-food multivitamin proanthocyanidin blend	NA	pine bark extract	Botanical
joint support	Standar d	oligomeric proanthocyanidins	4	NA	NA
body wise optimeyes	Standar d	bilberry fruit extract 100:1 (25% anthocyanidins)	3	NA	NA
isotonix opc-3 oligomeric proanthocyanidins	Standar d	calories	4	NA	NA
isotonix opc-3 oligomeric proanthocyanidins	Standar d	citrus bioflavonoids	3	NA	NA
isotonix opc-3 oligomeric	Standar	grape seed extract	3	NA	NA

proanthocyanidins	d				
isotonix opc-3 oligomeric proanthocyanidins	Standard	potassium	4	NA	NA
isotonix opc-3 oligomeric proanthocyanidins	Standard	sugars	4	NA	NA
isotonix opc-3 oligomeric proanthocyanidins	Standard	total carbohydrate	4	NA	NA
isotonix opc-3 oligomeric proanthocyanidins	Standard	pine bark extract	3	NA	NA
isotonix opc-3 oligomeric proanthocyanidins	Standard	bilberry extract	3	NA	NA
isotonix opc-3 oligomeric proanthocyanidins	Standard	red wine extract	4	NA	NA
nature's sunshine high potency grapine proanthocyanidins 60 mg	Standard	calcium	2	NA	NA
nature's sunshine high potency grapine proanthocyanidins 60 mg	Standard	phosphorus	2	NA	NA
nature's sunshine high potency grapine proanthocyanidins 60 mg	Standard	grapine (from grape seed and pine bark)	3	NA	NA
roex procyanidin 95 (pc-95) the super antioxidant 30 mg grape seed extract	Standard	grape seed extract	3	NA	NA
hhf-healthy heart formula	Standard	red wine proanthocyanidins	4	NA	NA
oxyspectro	Standard	red wine proanthocyanidins	4	NA	NA
natural factors eye factors with 2 mg lutein	Standard	multi-anthocyanidins powdered extract (fruits)	3	NA	NA
supervision eye formula webber naturals	Standard	multi-anthocyanidins	4	NA	NA
flora bilberry extract 25% standardized anthocyanidins with freeze-dried blueberry powder	Standard	blueberry powder	3	NA	NA
flora bilberry extract 25% standardized anthocyanidins with freeze-dried blueberry powder	Standard	bilberry (25% standardized extract)	3	NA	NA
oxyplus	Standard	red wine proanthocyanidins	4	NA	NA
generic8 super vision eye formula	Standard	multi-anthocyanidins powdered extract (fruits)	3	NA	NA
purity products omega berry super formula	Standard	anthocyanidins	4	NA	NA
basic's grape seed extract 100 mg (95% proanthocyanidins with bioflavonoids) basic vitamins	Standard	citrus bioflavonoids	3	NA	NA
basic's grape seed extract 100 mg (95% proanthocyanidins with bioflavonoids) basic vitamins	Standard	grape seed extract (95%)	3	NA	NA

life extension mix capsules state-of-the-art multi-nutrient formula	Standard	cyanidin-3-glucoside (c3g)	4	NA	NA
nature's plus source of life green and red mini-tabs multi-vitamin & mineral with whole food concentrates 500 mg spirulina exotic red fruits vegetarian	Standard	proanthocyanidins	4	NA	NA
gaia herbs hawthorn supreme	Standard	oligomeric procyanidins	4	NA	NA
nature's plus source of life green and red multi-vitamin & mineral with whole food concentrates 500 mg spirulina exotic red fruits vegetarian orac value 1000	Standard	proanthocyanidins	4	NA	NA
isotonix opc-3 (oligomeric proanthocyanidins)	Standard	calories	4	NA	NA
isotonix opc-3 (oligomeric proanthocyanidins)	Standard	grape seed extract	3	NA	NA
isotonix opc-3 (oligomeric proanthocyanidins)	Standard	potassium	4	NA	NA
isotonix opc-3 (oligomeric proanthocyanidins)	Standard	sugars	4	NA	NA
isotonix opc-3 (oligomeric proanthocyanidins)	Standard	total carbohydrate	4	NA	NA
isotonix opc-3 (oligomeric proanthocyanidins)	Standard	pine bark extract	3	NA	NA
isotonix opc-3 (oligomeric proanthocyanidins)	Standard	bilberry extract	3	NA	NA
isotonix opc-3 (oligomeric proanthocyanidins)	Standard	red wine extract	4	NA	NA
isotonix opc-3 (oligomeric proanthocyanidins)	Standard	citrus bioflavonoids extract	4	NA	NA
life extension life extension mix tablets state-of-the-art multi-nutrient formula	Standard	cyanidin-3-glucoside (c3g)	4	NA	NA
life extension life extension mix tablets state-of-the-art multi-nutrient formula	Standard	cherrypure tart cherry proanthocyanidin extract (skin)	4	NA	NA
life extension life extension mix tablets state-of-the-art multi-nutrient formula	Standard	leucoselect grape seed proanthocyanidin extract	4	NA	NA
life extension life extension mix tablets state-of-the-art multi-nutrient formula	Standard	biovin grape proanthocyanidin extract (whole grapes)	4	NA	NA
trader joe's super vision	Standar	multi-	3	NA	NA

eye formula	d	anthocyanidins powdered extract (fruits)			
natrol pycnogenol 50 mg standardized pine bark extract	Standard	oligomeric proanthocyanidins	4	NA	NA
cell nutritionals grape seed extract 50 mg rich in oligomeric proanthocyanidins	Standard	grape seed extract (95%)	3	NA	NA
Search for “delphinidin”					
life extension life extension mix tablets state-of-the-art multi-nutrient formula	Standard	delphinidins	4	NA	NA
Search for “delphinidin”					
limbrel 500 flavocoxid and citrated zinc bisglycinate 500 mg/50 mg	Standard	limbrel flavocoxid proprietary blend	NA	epicatechin	Other
Search for “epigallocatechin”					
nutrilite concentrated fruits and vegetables phytonutrient	Standard	egcg (epigallocatechin gallate)	4	NA	NA
natrol acaiberry diet acai & green tea super foods	Standard	egcg (epigallocatechin gallate)	4	NA	NA
natrol carb intercept 3 1 - 2 - 3 lean phase 2 clinically validated white kidney bean extract	Standard	egcg (epigallocatechin gallate)	4	NA	NA
breast health phytonutrient anti-oxidants indole-3-carbinol sulforaphane procaps laboratories	Standard	egcg (epigallocatechin gallate)	4	NA	NA
women's wellness procaps laboratories	Standard	egcg (epigallocatechin gallate)	4	NA	NA
ultimate anti-oxidant extracts world health, spice of life, fruit, berry & cruciferous extracts procaps laboratories	Standard	egcg (epigallocatechin gallate)	4	NA	NA
rexall sundown naturalist one daily energy multivitamin with green tea extract (egcg)	Standard	egcg (epigallocatechin gallate)	4	NA	NA
swanson ultra egcg super-strength green tea 275 mg	Standard	egcg (epigallocatechin gallate)	4	NA	NA
walgreens green tea with egcg (green tea extract)	Standard	egcg (epigallocatechin gallate)	4	NA	NA
Search for “catechin”					
great earth nsp daily nutritional supplement program regular strength	Standard	great earth broad spectrum phyto-antioxidant actives	NA	catechins	Other
great earth super oxy e 400 extra strength	Standard	great earth broad spectrum phyto-antioxidant actives	NA	catechins	Other
heba spring green tea	Standar	heba spring green	NA	green tea	Botanical

enriched catechins and theanine tea concentrate 3,000 orac+	d	tea herbal blend			
heba spring green tea enriched catechins and theanine tea concentrate 3,000 orac+	Standard	heba spring green tea herbal blend	NA	fruit of paradise (luo han kuo)	Botanical
heba spring green tea enriched catechins and theanine tea concentrate 3,000 orac+	Standard	heba spring green tea herbal blend	NA	blackberry extract	Botanical
heba green tea orac concentrated catechins tea concentrate 3,000 orac+	Standard	heba green tea orac herbal blend	NA	green tea leaves	Botanical
heba green tea orac concentrated catechins tea concentrate 3,000 orac+	Standard	heba green tea orac herbal blend	NA	blackberry extract	Botanical
heba green tea orac concentrated catechins tea concentrate 3,000 orac+	Standard	heba green tea orac herbal blend	NA	luo han (cucurbitacea)	Botanical
juice plus+ orchard blend chewables	Standard	juice plus+ orchard proprietary blend	NA	polyphenol catechins	Other
juice plus+ garden blend chewables	Standard	juice plus+ garden proprietary blend	NA	polyphenol catechins	Other
ritestart women all-in-one nutrition for women with 4life transfer factor plus exclusive anti-aging formula 4life packet	Standard	4life proprietary antioxidant blend	NA	catechins	Other
ritestart men all-in -one nutrition for men with 4life transfer factor plus advanced formula exclusive anti-aging formul	Standard	4life proprietary antioxidant blend	NA	catechins	Other
valerie saxion's silver creek labs, ltd. smart focus	Standard	valerie saxion's silver creek labs, ltd. smart focus proprietary blend	NA	catechins	Other
limbrel 500 flavocoxid and citrated zinc bisglycinate 500 mg/50 mg	Standard	limbrel flavocoxid proprietary blend	NA	catechin	Other
limbrel 500 flavocoxid and citrated zinc bisglycinate 500 mg/50 mg	Standard	limbrel flavocoxid proprietary blend	NA	epicatechin	Other
bpi sports 'the experience' pump hd advanced formula! 1 g cyclocreatine pcr 1 g glycerol 2x-rs	Standard	bpi sports 'the experience' pump hd adaptogenic and erythropoietin (epo) matrix (proprietary)	NA	green tea leaf catechin 98% extract	Other
oxyspectro	Standard	tea catechin powdered extract	3	NA	NA
pharmanex lifepak new anti-aging formula	Standard	catechins (camellia leaf extract 20:1)	3	NA	NA
new vitality royal greens	Standard	green tea catechins	3	NA	NA

jarrow formulas green tea 500 mg 5:1 water extract 40% polyphenols (30% catechins) 500 mg	Standard	green tea extract	3	NA	NA
metagenics metaglycemx	Standard	catechins	4	NA	NA
all day energy greens hi-octane energy drink all natural institute for vibrant living ivl	Standard	green tea catechins	3	NA	NA
frs antioxidant energy chews	Standard	catechins	4	NA	NA
pharmanex lifepak nano	Standard	catechins (camellia leaf extract 20:1)	3	NA	NA
pharmanex lifepak prime anti-aging packets	Standard	catechins (camellia leaf extract 20:1)	3	NA	NA
bluebonnet herbals egcg green tea leaf extract providing 200 mg egcg	Standard	catechins	4	NA	NA
frs healthy energy with quercetin & 7 vitamins soft chews	Standard	catechins	4	NA	NA
nutrilite concentrated fruits and vegetables phytonutrient	Standard	egcg (epigallocatechin gallate)	4	NA	NA
natrol acaiberry diet acai & green tea super foods	Standard	egcg (epigallocatechin gallate)	4	NA	NA
natrol carb intercept 3 1 - 2 - 3 lean phase 2 clinically validated white kidney bean extract	Standard	egcg (epigallocatechin gallate)	4	NA	NA
breast health phytonutrient anti-oxidants indole-3-carbinol sulforaphane procaps laboratories	Standard	egcg (epigallocatechin gallate)	4	NA	NA
women's wellness procaps laboratories	Standard	egcg (epigallocatechin gallate)	4	NA	NA
ultimate anti-oxidant extracts world health, spice of life, fruit, berry & cruciferous extracts procaps laboratories	Standard	egcg (epigallocatechin gallate)	4	NA	NA
pharmanex lifepak nano	Standard	catechins	4	NA	NA
rexall sundown naturalist one daily energy multivitamin with green tea extract (egcg)	Standard	egcg (epigallocatechin gallate)	4	NA	NA
apex energetics neuroflam (k-46)	Standard	catechins	4	NA	NA
default barley grass powder packs	Standard	catechins	4	NA	NA
swanson ultra egcg super-strength green tea 275 mg	Standard	egcg (epigallocatechin gallate)	4	NA	NA
walgreens green tea with egcg (green tea extract)	Standard	egcg (epigallocatechin gallate)	4	NA	NA
Search for “gallocatechin”					
nutrilite concentrated	Standar	egcg	4	NA	NA

fruits and vegetables phytonutrient	d	(epigallocatechin gallate)			
natrol acaiberry diet acai & green tea super foods	Standard	egcg (epigallocatechin gallate)	4	NA	NA
natrol carb intercept 3 1 - 2 - 3 lean phase 2 clinically validated white kidney bean extract	Standard	egcg (epigallocatechin gallate)	4	NA	NA
breast health phytonutrient anti-oxidants indole-3-carbinol sulforaphane procaps laboratories	Standard	egcg (epigallocatechin gallate)	4	NA	NA
women's wellness procaps laboratories	Standard	egcg (epigallocatechin gallate)	4	NA	NA
ultimate anti-oxidant extracts world health, spice of life, fruit, berry & cruciferous extracts procaps laboratories	Standard	egcg (epigallocatechin gallate)	4	NA	NA
rexall sundown naturalist one daily energy multivitamin with green tea extract (egcg)	Standard	egcg (epigallocatechin gallate)	4	NA	NA
swanson ultra egcg super-strength green tea 275 mg	Standard	egcg (epigallocatechin gallate)	4	NA	NA
walgreens green tea with egcg (green tea extract)	Standard	egcg (epigallocatechin gallate)	4	NA	NA
Search for “theaflavin”					
life extension arthromax with theaflavins & apresflex	Standard	boron	2	NA	NA
life extension arthromax with theaflavins & apresflex	Standard	glucosamine sulfate . 2 kcl	4	NA	NA
life extension arthromax with theaflavins & apresflex	Standard	msm (methylsulfonylmethane)	4	NA	NA
life extension arthromax with theaflavins & apresflex	Standard	apresflex (boswellia serrata) extract (gum resin)	3	NA	NA
life extension arthromax with theaflavins & apresflex	Standard	black tea theaflavins decaffeinated extract (leaf)	4	NA	NA
Search for “eriodictyol”					
lipo-flavonoid plus extra strength	Standard	lipo-flavonoid plus bioflavonoids	NA	eriodictyol glycoside	Other
lipo-flavonoid plus extra strength proprietary formula unique inner ear health formula	Standard	lipo-flavonoid plus extra strength proprietary blend	NA	eriodictyol glycoside	Other
Search for “naringenin”					
garden of life immune balance daily whole food	Standard	garden of life immune balance daily citrus c blend	NA	naringenin	Other
twinlab citrus bioflavonoid caps with rutin	Standard	twinlab citrus bioflavonoid caps citrus	NA	naringenin	Other

		bioflavonoids complex			
bluebonnet c-1000 plus bioflavonoids vitamin c plus citrus bioflavonoids	Standard	naringen & naringenin	4	NA	NA
default citrus bioflavonoids	Standard	naringen & naringenin	4	NA	NA
botanic choice bilberry plus mirtoselect	Standard	naringin, naringenin, 7-b-rutinoside and others	4	NA	NA
Search for "apigenin"					
nature's way chamomile standardized 1.2% apigenin	Standard	chamomile (flowers)	3	NA	NA
nature's way chamomile standardized 1.2% apigenin	Standard	chamomile, dried extract	3	NA	NA
nature's plus herbal actives oliceutic-20 standardized olive leaf 250 mg 20-25% oleuropein	Standard	apigenin-7-o-glucoside	4	NA	NA
apex energetics neuroflam (k-46)	Standard	apigenin	4	NA	NA
Search for "luteolin"					
life extension mix caps state-of-the-art multi-nutrient formula	Standard	luteolin	4	NA	NA
nature's plus herbal actives oliceutic-20 standardized olive leaf 250 mg 20-25% oleuropein	Standard	luteolin	4	NA	NA
nature's plus herbal actives oliceutic-20 standardized olive leaf 250 mg 20-25% oleuropein	Standard	luteolin-7-o-glucoside	4	NA	NA
life extension mix capsules state-of-the-art multi-nutrient formula	Standard	luteolin	4	NA	NA
life extension life extension mix tablets state-of-the-art multi-nutrient formula	Standard	luteolin	4	NA	NA
apex energetics neuroflam (k-46)	Standard	luteolin	4	NA	NA
pure encapsulations lvr formula	Standard	luteolin	4	NA	NA
Search for "kaempferol"					
valerie saxion's silver creek labs, ltd. smart focus	Standard	valerie saxion's silver creek labs, ltd. smart focus proprietary blend	NA	kaempferol	Other
Search for "myricetin"					
cellucor p6 extreme advanced anabolic stack	Standard	cellucor p6 extreme blend	NA	myricetin	Other
Search for "quercetin"					
nutrilite double x multivitamin multimineral	Standard	double x concentrate	NA	quercetin	Other
herbalife 21 day herbal cleansing program	Standard	herbalife am exclusive blend	NA	quercetin	Other

shaklee cartomax	Standard	cartomax proprietary blend	NA	quercetin	Other
provex cv	Standard	provex flavonoid complex	NA	quercetin	Other
usana essentials mega antioxidant	Standard	usana bioflavonoid complex	NA	quercetin	Other
usana essentials mega antioxidant with patented olivol	Standard	usana bioflavonoid complex	NA	quercetin	Other
mini's vitamin mineral & herbal formula essentials by megafood full color spectrum foodbased nutrition 72% whole food	Standard	essentials mini's food base blend	NA	quercetin	Other
rbc microhydrin plus with 8 additional antioxidants	Standard	rbc proprietary antioxidant blend	NA	quercetin	Other
ultra body toddy with cell shield	Standard	ultra body toddy cell shield proprietary blend	NA	quercetin	Other
freelife feeling young premium multi-vitamin & mineral with 100 anti-aging nutrients	Standard	feeling young fruit and vegetable extract complex	NA	quercetin	Other
nature's sunshine perfect eyes	Standard	nature's sunshine perfect eyes proprietary blend	NA	quercetin	Other
dr. julian whitaker's forward plus daily regimen	Standard	forward multi-nutrient daily bioflavonoids proprietary complex	NA	quercetin	Other
bioset gastro calm hypoallergenic enzyme	Standard	bioset gastro calm proprietary herbal blend	NA	quercetin	Other
twinlab ripped fuel ephedra free metabolic enhancer	Standard	twinlab ripped fuel metabolic fat loss blend	NA	quercetin	Other
twinlab ripped fuel ephedra free metabolic enhancer definition	Standard	twinlab ripped fuel metabolic fat loss blend	NA	quercetin	Other
allergex all natural	Standard	allergex proprietary blend	NA	quercetin	Other
bronson msm complex	Standard	bronson msm complex proprietary blend	NA	quercetin	Other
mannatech optimal health system ambrotose ao antioxidant cell protection formula with immune support glyco-antioxidant	Standard	mannatech mtech ao blend	NA	quercetin dihydrate	Other
your vitamins andrew lessman circulation & vein support bioflavonoid complex diosmin hesperidin quercetin rutin	Standard	your vitamins flavonol complex	NA	flavonols	Other
your vitamins andrew lessman circulation & vein support bioflavonoid complex diosmin hesperidin quercetin rutin	Standard	your vitamins flavonol complex	NA	flavones	Other
your vitamins andrew lessman circulation & vein	Standard	your vitamins flavonol complex	NA	phenols	Other

support bioflavonoid complex diosmin hesperidin quercetin rutin					
super nutrition simply one men one-per-day high energy multi-vitamin multi-mineral multi-vitamin/mineral with herbs	Standard	supernutrition phyto-antioxidant blend	NA	quercetin	Other
super nutrition simply one men one-per-day	Standard	supernutrition phyto-antioxidant blend	NA	quercetin	Other
super nutrition simply one women	Standard	supernutrition phyto-antioxidant blend	NA	quercetin	Other
super nutrition simply one women one-per-day multi-vitamin/mineral	Standard	supernutrition phyto-antioxidant blend	NA	quercetin	Other
ritestart women all-in-one nutrition for women with 4life transfer factor plus exclusive anti-aging formula 4life packet	Standard	4life women's health blend	NA	quercetin	Other
ritestart men all-in -one nutrition for men with 4life transfer factor plus advanced formula exclusive anti-aging formul	Standard	4life men's health blend	NA	quercetin	Other
usana healthpak 100	Standard	usana bioflavonoid complex #2	NA	quercetin	Other
usana essentials mega antioxidant with patented olivol	Standard	usana bioflavonoid complex #2	NA	quercetin	Other
goldshield elite ultimate antioxidant	Standard	goldshield elite bioflavonoid complex	NA	quercetin	Other
cytogenix laboratories hardcore strength xenadrine rfa-x extreme biochemical fat-burning agent ultra potent fat-burning	Standard	cytogenix laboratories hardcore strength xenadrine rfa-x proprietary blend	NA	quercetin dihydrate	Other
usana essentials body rox vitamin & mineral	Standard	usana essentials body rox antioxidant phytonutrient complex	NA	quercetin	Other
valerie saxion's silver creek labs, ltd. smart focus	Standard	valerie saxion's silver creek labs, ltd. smart focus proprietary blend	NA	quercetin	Other
daily for life for women am/pm high potency packets	Standard	daily for life for women proprietary blend #2	NA	quercetin	Other
daily power packs for women arbonne smart nutritional hybrids	Standard	arbonne high orac complex	NA	quercetin	Other
hydroxycut lose weight fast rapid release caplets	Standard	hydroxycut lose weight fast hydroxytea blend	NA	quercetin dihydrate	Other
seniorlife health prostate health essentials with saw	Standard	seniorlife health prostate health	NA	quercetin dihydrate	Other

palmetto, beta-sitosterol, & selenium		essentials proprietary blend			
trace minerals research greens pak liquimins green foods vegetables fruits super fruits fiber antioxidants enzymes probi	Standard	trace minerals research greens pak antioxidant blend	NA	quercetin	Other
resvinatrol complete advanced resveratrol coq10 & omega 3 formula with plant sterols 4000 orac per serving alcohol free	Standard	resvinatrol complete proprietary healthy heart blend	NA	quercetin	Other
healthy habits maximum wellness mixed berry aloe tonic	Standard	healthy habits maximum wellness proprietary fruit, vegetable and bioflavonoid blend	NA	quercetin	Other
maxgxl 'the glutathione accelerator'	Standard	maxgxl proprietary gsh absorption & recycling blend	NA	quercetin	Other
mannatech optimal health system optimal support packets phytomatrix, plus and ambrotose ao	Standard	mannatech mtech ao blend #2	NA	quercetin dihydrate	Other
mannatech ambrotose ao glyco-antioxidant antioxidant cell protection formula with immune support	Standard	mannatech mtech ao blend #2	NA	quercetin dihydrate	Other
kenzen mega daily 4 for men nikken	Standard	kenzen mega daily 4 for men prostate blend	NA	quercetin	Other
melaleuca vitality total essentials + heart + body 9 patented or proprietary formulas am & pm packets for men 50+	Mature	melaleuca provex cv proprietary blend	NA	quercetin	Other
animal ripped & peeled cuts animal training packs	Standard	animal ripped & peeled cuts bioavailability complex	NA	quercetin	Other
delicious greens 8000 the original!	Standard	delicious greens 8000 the original! xtraimmunaid blend	NA	quercetin	Other
c-macs mineral ascorbates mcdonagh medical center, inc. for adults	Standard	c-macs mcdonagh medical center, inc. proprietary complex	NA	quercetin dihydrate	Other
animal flex	Standard	animal flex joint support complex	NA	quercetin	Other
melaleuca provexcv patented blend of grape seed and skin, resveratrol, green tea, quercetin, and enzymes	Standard	melaleuca provexcv grape proprietary blend	NA	grape seed extract	Botanical
melaleuca provexcv patented blend of grape seed and skin, resveratrol, green tea, quercetin, and	Standard	melaleuca provexcv grape proprietary blend	NA	grape skin extract	Botanical

enzymes					
melaleuca provexcv patented blend of grape seed and skin, resveratrol, green tea, quercetin, and enzymes	Standard	melaleuca provexcv proprietary blend	NA	ginkgo biloba leaf extract	Botanical
melaleuca provexcv patented blend of grape seed and skin, resveratrol, green tea, quercetin, and enzymes	Standard	melaleuca provexcv proprietary blend	NA	fungal protease	Other
melaleuca provexcv patented blend of grape seed and skin, resveratrol, green tea, quercetin, and enzymes	Standard	melaleuca provexcv proprietary blend	NA	bromelain protease	Other
melaleuca provexcv patented blend of grape seed and skin, resveratrol, green tea, quercetin, and enzymes	Standard	melaleuca provexcv proprietary blend	NA	bilberry extract (berry)	Botanical
melaleuca provexcv patented blend of grape seed and skin, resveratrol, green tea, quercetin, and enzymes	Standard	melaleuca provexcv proprietary blend	NA	quercetin powder	Other
melaleuca vitality total essentials + heart + body 9 patented or proprietary formulas am & pm packets for men	Standard	melaleuca provexcv proprietary blend	NA	quercetin powder	Other
melaleuca vitality 6 essentials + heart 6 patented or proprietary formulas am & pm packets for women	Standard	melaleuca provexcv proprietary blend	NA	quercetin powder	Other
melaleuca men's peak performance nutrition pack 6 patented or proprietary formulas am & pm packets	Standard	melaleuca provexcv proprietary blend	NA	quercetin powder	Other
melaleuca women's peak performance nutrition pack 6 patented or proprietary formulas am & pm packets	Standard	melaleuca provexcv proprietary blend	NA	quercetin powder	Other
berry essentials ivl institute for vibrant living	Standard	berry essentials fruit & vegetable blend	NA	quercetin	Other
country life buffer-c ph controlled 500 mg vitamin c	Standard	country life buffer-c proprietary mineral blend	NA	quercetin	Other
doterra alpha crs+ cellular vitality complex	Standard	doterra alpha crs+ cellular energy blend	NA	quercetin	Other
ultra liquid zeolite micronized zeolite liquid concentrate vegetarian formula	Standard	ultra liquid zeolite proprietary blend	NA	dhq (dihydroquercetin)	Other
controlled labs orange triad multi-vitamin, joint, digestion & immune formula	Standard	controlled labs orange triad digestion and immune complex	NA	quercetin	Other

purity products krillberry krill & omega-3 fish oil formula	Standard	purity products krillberry proprietary super fruit extract blend	NA	quercetin	Other
purity products perfect multi super greens	Standard	purity products perfect multi super greens purity's dark greens and super food phyto-complex	NA	quercetin	Other
usana mega antioxidant	Standard	usana bioflavonoid complex #3	NA	quercetin	Other
usana healthpak	Standard	usana bioflavonoid complex #3	NA	quercetin	Other
animal flex animal training packs	Standard	animal flex joint support complex #2	NA	quercetin	Other
cellgevity	Standard	cellgevity proprietary blend	NA	quercetin	Other
purity products dr. cannell's advanced d vitamin d super formula	Standard	purity products dr. cannell's advanced d vitamin d co-factor proprietary blend	NA	quercetin	Other
gnc beyond raw re-feed nighttime, super-anabolic recovery protein	Standard	gnc beyond raw re-feed antioxidant system	NA	quercetin	Other
ceraplex (neuro-protector) brain detox formula!	Standard	ceraplex proprietary blend	NA	quercetin	Other
isagenix ageless essentials with product b women's formula antioxidants plus telomere a.m. & p.m. packs	Standard	isagenix product b proprietary blend	NA	quercetin	Other
velocity 1 vizn	Standard	velocity 1 vizn proprietary blend	NA	quercetin	Other
super nutrition simplyone women triple power! non-gmo	Standard	super nutrition simplyone women antioxidant blend	NA	quercetin	Other
amazing grass green superfood 40,000 orac units per serving	Standard	amazing grass green superfood amazing grass high antioxidant blend	NA	quercetin	Other
activit vitamins for active people 22 vitamins and minerals plus 6 antioxidant phytonutrients beachbody	Standard	activit vitamins for active people phytonutrient blend	NA	quercetin	Other
animal cuts ripped & peeled animal training packs	Standard	animal cuts ripped & peeled bioavailability complex	NA	quercetin	Other
doterra alpha crs+ cellular vitality complex	Standard	doterra alpha crs+ cellular energy blend #2	NA	quercetin	Other
gnc superfoods ultra mega green men's whole food enhanced multivitamin clinically studied	Standard	gnc ultra mega green fruit & vegetable antioxidant blend (spectra total orac	NA	quercetin	Other

		blend)			
gnc superfoods ultra mega green women's whole food enhanced multivitamin clinically studied	Standard	gnc ultra mega green fruit & vegetable antioxidant blend (spectra total orac blend)	NA	quercetin	Other
p90x daily nutritional advantage 22 vitamins and minerals plus 6 antioxidant phytonutrients beachbody	Standard	p90x phytonutrient blend	NA	quercetin	Other
lifevantage protandim nrf1 synergizer new breakthrough	Standard	lifevantage protandim nrf1 proprietary blend	NA	quercetin	Other
lifevantage axio	Standard	lifevantage axio (regular) proprietary blend	NA	quercetin dihydrate	Other
reserveage nutrition ultra collagen booster with biocell collagen and dermaval	Standard	reserveage nutrition ultra collagen booster dermaval	NA	quercetin	Other
mp musclepharm armor-v multi-nutrient complex the athletes daily vitamin with minerals, vitamins & antioxidants balanced combination of omega fatty acids	Standard	mp musclepharm armor-v armor blend	NA	quercetin	Other
bpi sports 'the experience' pump hd advanced formula! 1 g cyclocreatine pcr 1 g glycerol 2x-rs	Standard	bpi sports 'the experience' pump hd adaptogenic and erythropoietin (epo) matrix (proprietary)	NA	quercetin dihydrate	Other
country life core daily-1 multivitamins 1 daily women 50+ one tablet includes: 200+ mg of women's health blend over 30 raw whole foods 'coenzymated' b vitamins and probiotics, digestive enzymes	Mature	country life core daily-1 multivitamins 1 daily women 50+ proprietary blend	NA	quercetin	Other
forever daily vitamins and minerals with aos complex forever	Standard	forever daily aos complex advanced cellular support blend	NA	quercetin	Other
dynamic fruits & greens with superfruitox proprietary blend of organic super fruits, acai, goji, mangosteen, noni, & pomegranate	Standard	dynamic fruits & greens superfruitox antioxidant blend	NA	quercetin dihydrate	Other
gat muscle martini the ultimate amino superdrink muscle building eaas and bcaas 30 fruit, vegetable & herb extracts clinically based formula carbs 2 calories 10 sugars 0	Standard	gat muscle martini 5-dimensional antioxidant & recovery blend	NA	quercetin	Other

trader joe's super red drink powder	Standard	trader joe's super red drink powder proprietary antioxidant blend #3	NA	quercetin	Other
pure essence labs one 'n' only women uniquely feminine formula multivitamin & mineral 23,700 mg whole food value	Standard	pure essence labs one 'n' only bioflavonoids	NA	quercetin	Other
lifepak	Standard	quercetin	4	NA	NA
lifepak prime	Standard	quercetin	4	NA	NA
lifepak women	Standard	quercetin	4	NA	NA
lifepak trim	Standard	quercetin	4	NA	NA
cartilage formula pharmanex	Standard	quercetin	4	NA	NA
nutrilite double x multivitamin multimineral	Standard	quercetin	4	NA	NA
jenny craig protect + antioxidant formula with phytonutrients	Standard	quercetin	4	NA	NA
vitamin world green source multivitamins & minerals with whole food concentrates vegetarian formula	Standard	quercetin	4	NA	NA
puritan's pride green source multivitamins & minerals with whole food concentrates vegetarian formula	Standard	quercetin	4	NA	NA
atkins basic # 3 vita-nutrient formula	Standard	quercetin	4	NA	NA
amni bioflavonoid complex	Standard	quercetin	4	NA	NA
gnc multi ultra mega gold timed release	Standard	quercetin dihydrate	4	NA	NA
gnc multi ultra mega gold without iron timed release	Standard	quercetin dihydrate	4	NA	NA
oxyfresh the ultimate multivitamin mineral complex	Standard	quercetin	4	NA	NA
solgar earth source multi-nutrient	Standard	quercetin	4	NA	NA
bio-recovery inc. bio-ester c: esterified calcium ascorbate	Standard	quercetin	4	NA	NA
dr. julian whitaker's vision essentials	Standard	quercetin	4	NA	NA
body wise optimeyes	Standard	quercetin dihydrate	4	NA	NA
body design overdrive pharmanex	Standard	quercetin	4	NA	NA
metagenics fem essentials	Standard	quercetin	4	NA	NA
nature's plus source of life multivitamin and mineral	Standard	quercetin	4	NA	NA

with whole food concentrates					
twinlab joint fuel	Standard	quercetin	4	NA	NA
oxitrol	Standard	quercetin	4	NA	NA
ortho molecular products mucosagen	Standard	quercetin	4	NA	NA
atkins basic # 3 targeted multi-vitamin formula	Standard	quercetin	4	NA	NA
mountain home daily advantage multi-nutrient	Standard	quercetin	4	NA	NA
watkins superfood multiple	Standard	quercetin	4	NA	NA
maxivision whole body formula complete multivitamin with lutein elemental antioxidant formula	Standard	quercetin	4	NA	NA
twinlab ocuguard plus with lutein	Standard	quercetin dihydrate	4	NA	NA
metagenics multigenics without iron	Standard	quercetin	4	NA	NA
metagenics multigenics chewable	Infant/pediatric	quercetin	4	NA	NA
purity's perfect multi advanced formula multi-vitamin, mineral, herbal phyto-nutrient super formula without iron	Standard	quercetin	4	NA	NA
purity's ultimate h.a. formula synergistic h.a. blend with hyaluronic acid, purity's advanced formula	Standard	quercetin dihydrate	4	NA	NA
natural factors quercetin bioflavonoid complex	Standard	total carbohydrate	4	NA	NA
natural factors quercetin bioflavonoid complex	Standard	citrus bioflavonoids powdered extract	3	NA	NA
natural factors quercetin bioflavonoid complex	Standard	quercetin powdered extract	3	NA	NA
natural factors quercetin bioflavonoid complex	Standard	bromelain powdered extract (pineapple stem)	3	NA	NA
natural factors quercetin bioflavonoid complex	Standard	rutin powdered extract	3	NA	NA
natural factors eye factors with 2 mg lutein	Standard	quercetin powdered extract	3	NA	NA
supervision eye formula webber naturals	Standard	quercetin	4	NA	NA
atkins basic # 1 targeted multi-vitamin formula	Standard	quercetin	4	NA	NA
pharmanex lifepak new anti-aging formula	Standard	quercetin	4	NA	NA
pharmanex lifepak new anti-aging formula	Standard	quercetin	4	NA	NA
great earth nsp ii super c complex 500 regular strength timed release	Standard	hesperidin/quercetin complex	4	NA	NA
tropical oasis all in one plus liquid multi	Standard	quercetin	4	NA	NA
freelife soy miracle	Standar	quercetin	4	NA	NA

ultimate antioxidant full spectrum	d				
new image total	Standard	quercetin	4	NA	NA
nutrilite concentrated fruits and vegetables phytonutrient	Standard	quercetin	4	NA	NA
nutrilite chewable concentrated fruits and vegetables sweetened with sucrose, fructose, mannitol and xylitol	Standard	quercetin/quercetine	4	NA	NA
da vinci laboratories of vermont healthy eyes including lutein and lycopene	Standard	quercetin	4	NA	NA
herbalife thermojetics total control ephedra free	Standard	quercetin	4	NA	NA
glacial milk complete nutrition plant-source ionic minerals, vitamins, herbs, amino acids, lutein, coenzyme q10, una de	Standard	quercetin	4	NA	NA
maxivision ocular formula complete eye nutrients with lutein total eye support elemental antioxidant formula	Standard	quercetin	4	NA	NA
thorne research mediclear	Standard	quercetin chalcone	4	NA	NA
swanson condition specific formulas mobility essentials	Standard	quercetin	4	NA	NA
puritan's pride maximum antioxidant formula	Standard	quercetin	4	NA	NA
nature's blend super antioxidant with lutein and lycopene contains novasoy 20 mg standardized concentrated extract	Standard	quercetin	4	NA	NA
pathway to health reginald b. cherry, m.d. prostate support	Standard	quercetin	4	NA	NA
atkins seasonal care targeted nutritional formula	Standard	quercetin	4	NA	NA
oregon health multi-guard w/ coq10 a vitamin mineral anti-oxidant program	Standard	quercetin	4	NA	NA
nsp vi super c complex 1000 mg timed release extra strength	Standard	hesperidin/quercetin complex	4	NA	NA
perfectly balanced whole food concentrate multi vitamin & mineral all natural natural nutrition center	Standard	quercetin	4	NA	NA
kal enhanced energy full spectrum multiple with lutein	Standard	quercetin	4	NA	NA
daily nutritional support	Standar	quercetin	4	NA	NA

ultra multi-vitamin mineral superfood	d				
life enhancement energy cycle ii drink mix	Standard	quercetin	4	NA	NA
life enhancement super radical shield ii	Standard	quercetin	4	NA	NA
metagenics multigenics optimum multiple vitamin / mineral plan fast release tablet	Standard	quercetin	4	NA	NA
nature's plus source of life multivitamin & mineral with whole food concentrates no iron	Standard	quercetin	4	NA	NA
nature's plus source of life multivitamin & mineral with whole food concentrates	Standard	quercetin	4	NA	NA
gnc multi liquid ultra mega	Standard	quercetin	4	NA	NA
science based health ocular protect whole body formula	Standard	quercetin	4	NA	NA
isotonix vision formula with lutein	Standard	quercetin	4	NA	NA
julian whitaker, m.d. vision essentials advanced vision support system with bilberry and lutein	Standard	quercetin	4	NA	NA
puritan's pride eye-guard plus with lutein	Standard	quercetin (fruit)	3	NA	NA
gnc multi ultra mega green comprehensive whole food based multiple	Standard	quercetin dihydrate	4	NA	NA
life priority one-per-meal lifeguard	Standard	quercetin	4	NA	NA
maxivision liquid formulation whole body formula complete multivitamin with lutein total eye & body support	Standard	quercetin	4	NA	NA
nutura nutritional foundation	Standard	quercetin	4	NA	NA
radiance green source multi vitamins & minerals with whole food concentrates vegetarian formula	Standard	quercetin	4	NA	NA
schiff daily wellness multi-nutrient pack whole food based complete multivitamin vitamin mineral packets	Standard	quercetin	4	NA	NA
purity products perfect multi super greens optimally balanced, dark greens, phyto-nutrient, multivitamin and mineral sup	Standard	quercetin dihydrate	4	NA	NA
youngevity anti-aging daily premium pak	Standard	quercetin	4	NA	NA
advocare wellness system	Standar	quercetin	4	NA	NA

multinutrient	d				
procaps laboratories ultimate eye support 4 mg zeaxanthin 8 mg lutein bilberry, blueberry, blackberry and elderberry ext	Standard	quercetin	4	NA	NA
optim 3 premier formula for ocular nutrition with lutein complex	Standard	quercetin	4	NA	NA
ultra plan ultimate plus dieter's multi-vitamin	Standard	quercetin dihydrate	4	NA	NA
your vitamins andrew lessman circulation & vein support bioflavonoid complex diosmin hesperidin quercetin rutin	Standard	hesperidin	4	NA	NA
your vitamins andrew lessman circulation & vein support bioflavonoid complex diosmin hesperidin quercetin rutin	Standard	magnesium	2	NA	NA
your vitamins andrew lessman circulation & vein support bioflavonoid complex diosmin hesperidin quercetin rutin	Standard	quercetin	4	NA	NA
your vitamins andrew lessman circulation & vein support bioflavonoid complex diosmin hesperidin quercetin rutin	Standard	silicon	2	NA	NA
your vitamins andrew lessman circulation & vein support bioflavonoid complex diosmin hesperidin quercetin rutin	Standard	grape seed extract (seed)	3	NA	NA
your vitamins andrew lessman circulation & vein support bioflavonoid complex diosmin hesperidin quercetin rutin	Standard	vitamin c	1	NA	NA
your vitamins andrew lessman circulation & vein support bioflavonoid complex diosmin hesperidin quercetin rutin	Standard	eriocitrin	4	NA	NA
your vitamins andrew lessman circulation & vein support bioflavonoid complex diosmin hesperidin quercetin rutin	Standard	rutin	4	NA	NA
your vitamins andrew lessman circulation & vein support bioflavonoid complex diosmin hesperidin quercetin rutin	Standard	flavones	4	NA	NA
your vitamins andrew lessman circulation & vein support bioflavonoid complex diosmin hesperidin quercetin rutin	Standard	diosmin complex	4	NA	NA
generic8 super vision eye	Standar	quercetin	3	NA	NA

formula	d	powdered extract			
purity products h.a. joint formula	Standard	quercetin dihydrate	4	NA	NA
nature's way antioxidant formula 25,000 iu beta carotene plus coq10 & green tea	Standard	quercetin	4	NA	NA
vitamin world ocuplex with lutein and bilberry	Standard	quercetin	4	NA	NA
nature's bounty green source multi vitamins & minerals with whole food concentrates vegetarian formula	Standard	quercetin	4	NA	NA
irwin naturals prosta-strong with saw palmetto & 9 prostate support nutrients	Standard	quercetin	4	NA	NA
gnc preventive nutrition ocular formula	Standard	quercetin	4	NA	NA
solaray qbc plex quercetin, bromelain, vitamin c complex	Standard	quercetin	4	NA	NA
solaray qbc plex quercetin, bromelain, vitamin c complex	Standard	vitamin c	1	NA	NA
solaray qbc plex quercetin, bromelain, vitamin c complex	Standard	bromelain (1800 mcg)	4	NA	NA
stephen sinatra, m.d. prostate solutions advanced biosolutions	Standard	quercetin	4	NA	NA
default quercetin	Standard	quercetin	4	NA	NA
julian whitaker, m.d. forward plus daily regimen multi-nutrient	Standard	quercetin	4	NA	NA
puritan's pride green source multi vitamins & minerals with whole food concentrates	Standard	quercetin	4	NA	NA
pathway to healing reginald b. cherry, m.d. basic nutrient support packets	Standard	quercetin	4	NA	NA
metagenics sinuplex	Standard	quercetin	4	NA	NA
life extension resveratrol caps 100 mg	Standard	quercetin	4	NA	NA
swanson health products premium brand bilberry eyebright vision complex	Standard	quercetin dihydrate	4	NA	NA
procaps laboratories fruit full anti-oxidants bilberry cranberry blueberry grape strawberry mulberry elderberry cherry	Standard	quercetin	4	NA	NA
emergen-c joint health formula 500 mg glucosamine 400 mg chondroitin 1,000 mg vitamin c	Standard	quercetin	4	NA	NA

emergen-c 1,000 mg vitamin c as 7 mineral ascorbates 32 mineral complexes b vitamins fizzy drink mix lemon-lime	Standard	quercetin	4	NA	NA
gnc multivitamin ultra mega gold timed release caplets	Standard	quercetin dihydrate	4	NA	NA
genesis today 4 total nutrition liquid multiple vitamin, mineral & herbal	Standard	quercetin	4	NA	NA
swanson ultra whole food multi-vitamin & mineral without iron	Standard	quercetin dihydrate	4	NA	NA
frs antioxidant energy chews	Standard	quercetin	4	NA	NA
biotics research corporation bio-fcts	Standard	quercetin	4	NA	NA
purity products h.a. joint & skin super formula	Standard	quercetin dihydrate	4	NA	NA
source naturals life force multiple bio align	Standard	quercetin	4	NA	NA
metagenics multigenics without iron optimum multiple vitamin / mineral formula fast release tablet	Standard	quercetin	4	NA	NA
emergen-c heart health plant sterols & lycopene 1,000 mg vitamin c	Standard	quercetin	4	NA	NA
pharmanex lifepak nano	Standard	quercetin	4	NA	NA
life fitness ocu well with lutein & zinc	Standard	quercetin dihydrate	4	NA	NA
l.a. farmacia natural quercetin 500 plus 500 mg of quercetin per tablet	Standard	bromelain	4	NA	NA
l.a. farmacia natural quercetin 500 plus 500 mg of quercetin per tablet	Standard	citrus bioflavonoids	3	NA	NA
l.a. farmacia natural quercetin 500 plus 500 mg of quercetin per tablet	Standard	manganese	2	NA	NA
l.a. farmacia natural quercetin 500 plus 500 mg of quercetin per tablet	Standard	quercetin	4	NA	NA
l.a. farmacia natural quercetin 500 plus 500 mg of quercetin per tablet	Standard	vitamin c	1	NA	NA
l.a. farmacia natural quercetin 500 plus 500 mg of quercetin per tablet	Standard	tumeric extract (rhizome)	3	NA	NA
great earth vitamins ocular plus lutein	Standard	quercetin	4	NA	NA
procaps laboratories circulation & vein support diosmin hesperidin rutin	Standard	hesperidin	4	NA	NA

quercetin grape seed					
procaps laboratories circulation & vein support diosmin hesperidin rutin quercetin grape seed	Standard	quercetin	4	NA	NA
procaps laboratories circulation & vein support diosmin hesperidin rutin quercetin grape seed	Standard	silicon	2	NA	NA
procaps laboratories circulation & vein support diosmin hesperidin rutin quercetin grape seed	Standard	grape seed extract (seed)	3	NA	NA
procaps laboratories circulation & vein support diosmin hesperidin rutin quercetin grape seed	Standard	vitamin c	1	NA	NA
procaps laboratories circulation & vein support diosmin hesperidin rutin quercetin grape seed	Standard	rutin	4	NA	NA
procaps laboratories circulation & vein support diosmin hesperidin rutin quercetin grape seed	Standard	diosmin	4	NA	NA
procaps laboratories circulation & vein support diosmin hesperidin rutin quercetin grape seed	Standard	total flavonoids	4	NA	NA
arbonne bio-nutria joint formula	Standard	quercetin	4	NA	NA
nature's plus source of life vitamin, mineral & protein energy shake with whole food concentrates	Standard	quercetin	4	NA	NA
twinlab quercetin + c	Standard	vitamin c	1	NA	NA
twinlab quercetin + c	Standard	quercetin dihydrate	4	NA	NA
mms pro preventamins iron-free multivitamin/mineral high potency complex	Standard	quercetin	4	NA	NA
nutrilite kids chewable concentrated fruits and vegetables	Infant/pediatric	quercetin	4	NA	NA
vitol ener-g 1 per day multiple	Standard	quercetin	4	NA	NA
to your health liquid vitamin & mineral with amino acids, powerful antioxidants and healthy botanicals	Standard	quercetin	4	NA	NA
pharmanex lifepak prime anti-aging packets	Standard	quercetin	4	NA	NA
sbh science based health macular protect complete	Standard	quercetin	4	NA	NA
nutrilite antioxidant complex	Standard	quercetin	4	NA	NA
emergen-c immune defense formula 1,000 mg vitamin c enhanced zinc	Standard	quercetin	4	NA	NA

bluebonnet super earth multinutrient formula whole food based multivitamin & multimineral with pomegranate & sprout phyt	Standard	quercetin	4	NA	NA
the vitamin shoppe vitamins citrus free quercetin + c	Standard	quercetin	4	NA	NA
the vitamin shoppe vitamins citrus free quercetin + c	Standard	vitamin c	1	NA	NA
mannatech optimal health system glycentials antioxidant formula complete vitamin & mineral vitamin, ambroglycin mineral	Standard	quercetin	4	NA	NA
jarrow formulas quercetin 500 500 mg	Standard	quercetin	4	NA	NA
source naturals mega-kid chewable multi-vitamin for children ages 2-10	Infant/pediatric	quercetin	4	NA	NA
purity products the gold pack with coenzyme q10 elite multivitamin and mineral formula featuring a super greens blend	Standard	quercetin	4	NA	NA
emergen-c vitamin d & calcium bone health formula 500 mg calcium 1,000 iu vitamin d 500 mg vitamin c fizzy drink mix pac	Standard	quercetin	4	NA	NA
emergen-c 1,000 mg vitamin c 24 nutrients with antioxidants, electrolytes and 7 b vitamins lemon-lime fizzy drink mix pa	Standard	quercetin	4	NA	NA
source naturals advanced one multiple	Standard	quercetin	4	NA	NA
source naturals activated quercetin bioflavonoid complex	Standard	bromelain	4	NA	NA
source naturals activated quercetin bioflavonoid complex	Standard	magnesium	2	NA	NA
source naturals activated quercetin bioflavonoid complex	Standard	quercetin	4	NA	NA
source naturals activated quercetin bioflavonoid complex	Standard	total carbohydrate	4	NA	NA
source naturals activated quercetin bioflavonoid complex	Standard	vitamin c	1	NA	NA
synergistics symplex	Standard	quercetin	4	NA	NA
pure essence labs one 'n' only superior tonic multiple 6,000 mg whole	Standard	quercetin	4	NA	NA

food value					
vitalive	Standard	quercetin dihydrate	4	NA	NA
zahler allergease	Standard	quercetin	4	NA	NA
pure essence labs longevity anti-aging multiple women's formula	Standard	quercetin	4	NA	NA
nsi nutraceutical sciences institute synergy once daily multi-vitamin version 3	Standard	quercetin	4	NA	NA
emergen-c joint health formula 500 mg glucosamine hcl 400 mg chondroitin sulfate 1,000 mg vitamin c fizzy drink mix pack	Standard	quercetin	4	NA	NA
melaleuca provexcv patented blend of grape seed and skin, resveratrol, green tea, quercetin, and enzymes	Standard	green tea leaf extract	3	NA	NA
melaleuca provexcv patented blend of grape seed and skin, resveratrol, green tea, quercetin, and enzymes	Standard	resveratrol	4	NA	NA
genesis today 4 total nutrition liquid multiple vitamin, mineral & herbal	Standard	quercetin dihydrate	4	NA	NA
solaray circulegs horse chestnut special formula	Standard	quercetin	4	NA	NA
julian whitaker, m.d. vision essentials advanced vision support system with bilberry, lutein and zanthin astaxanthin	Standard	quercetin	4	NA	NA
gnc multivitamin ultra mega vegetarian multivitamin and mineral formula one caplet per day 75 mg b-complex 600 iu vitami	Standard	quercetin	4	NA	NA
shaklee vitalizer 80 bio-optimized nutrients vita-strips	Standard	quercetin	4	NA	NA
the vitamin shoppe antioxidants super antioxidant	Standard	quercetin	4	NA	NA
usana optimizers poly c	Standard	quercetin	4	NA	NA
bluebonnet liquid super earth multinutrient formula whole food based multivitamin & multimineral no iron added with vitamin d3 & pomegranate, mangosteen, goji & acai extracts	Standard	quercetin	4	NA	NA
projoba international projoba super daily food	Standard	quercetin	4	NA	NA

based vitamin / mineral complex					
invite aller clear hx	Standard	quercetin	4	NA	NA
coco berri ivl institute for vibrant living	Standard	quercetin	4	NA	NA
pathway to healing reginald b. cherry, m.d. basic nutrient support new & improved! packets	Standard	quercetin	4	NA	NA
swanson condition specific formulas vision essentials	Standard	quercetin	4	NA	NA
total inflam nutri-west	Standard	quercetin	4	NA	NA
emergen-c multi-vitamin + 500 mg vitamin c with b vitamins minerals and choline fizzy drink mix	Standard	quercetin	4	NA	NA
super nutrition super immune multivitamin	Standard	quercetin	4	NA	NA
frs healthy energy with quercetin & 7 vitamins soft chews	Standard	calories	4	NA	NA
frs healthy energy with quercetin & 7 vitamins soft chews	Standard	quercetin	4	NA	NA
frs healthy energy with quercetin & 7 vitamins soft chews	Standard	vitamin a	1	NA	NA
frs healthy energy with quercetin & 7 vitamins soft chews	Standard	vitamin e	1	NA	NA
frs healthy energy with quercetin & 7 vitamins soft chews	Standard	total fat	4	NA	NA
frs healthy energy with quercetin & 7 vitamins soft chews	Standard	sugars	4	NA	NA
frs healthy energy with quercetin & 7 vitamins soft chews	Standard	total carbohydrate	4	NA	NA
frs healthy energy with quercetin & 7 vitamins soft chews	Standard	calories from fat	4	NA	NA
frs healthy energy with quercetin & 7 vitamins soft chews	Standard	vitamin c	1	NA	NA
frs healthy energy with quercetin & 7 vitamins soft chews	Standard	niacin	1	NA	NA
frs healthy energy with quercetin & 7 vitamins soft chews	Standard	thiamin	1	NA	NA
frs healthy energy with quercetin & 7 vitamins soft chews	Standard	riboflavin	1	NA	NA
frs healthy energy with quercetin & 7 vitamins soft chews	Standard	vitamin b-6	1	NA	NA
frs healthy energy with quercetin & 7 vitamins soft chews	Standard	vitamin b-12	1	NA	NA

frs healthy energy with quercetin & 7 vitamins soft chews	Standard	catechins	4	NA	NA
designs for health ultimate antiox full spectrum	Standard	quercetin	4	NA	NA
emergen-c 1,000 mg vitamin c lemon-lime fizzy drink mix 24 nutrients with 7 b vitamins antioxidants and electrolytes	Standard	quercetin	4	NA	NA
maxivision ocular formula including lutein 20 mg, zeaxanthin 4 mg, coq10, acetyl-l-carnitine, green tea extract, resveratrol	Standard	quercetin	4	NA	NA
emergen-c immune +plus system support with vitamin d fizzy drink mix 1000 mg vitamin c and zinc 1000 iu vitamin d	Standard	quercetin	4	NA	NA
nature's plus source of life green and red mini-tabs multi-vitamin & mineral with whole food concentrates 500 mg spirulina exotic red fruits vegetarian	Standard	quercetin	4	NA	NA
nature's plus source of life multi-vitamin & mineral with whole food concentrates tablets vegetarian hypo-allergenic	Standard	quercetin	4	NA	NA
nature's plus source of life multi-vitamin & mineral with whole food concentrates no iron vegetarian hypo-allergenic	Standard	quercetin	4	NA	NA
purity products perfect multi super greens	Standard	quercetin	4	NA	NA
emergen-c 1,000 mg vitamin c super orange fizzy drink mix 24 nutrients with 7 b vitamins antioxidants and electrolytes	Standard	quercetin	4	NA	NA
pure essence labs one 'n' only prenatal multiple	Prenatal	quercetin	4	NA	NA
designs for health dfh complete multi with copper and iron	Standard	quercetin	4	NA	NA
jarrow formulas quercetin 500	Standard	quercetin	4	NA	NA
johnson up day down day diet eresveratrol-200	Standard	quercetin	4	NA	NA
nature's plus source of life green and red multi-vitamin & mineral with whole food concentrates	Standard	quercetin	4	NA	NA

500 mg spirulina exotic red fruits vegetarian orac value 1000					
maxivision whole body formula evidence based eye nutrients plus advanced daily multivitamin	Standard	quercetin	4	NA	NA
youthforia advanced red wine antioxidant anti-aging complex with resveratrol, vitamin d, acai, and coq10 rejuvenating formula	Standard	quercetin	4	NA	NA
vitacost synergy 3000 multi-vitamin	Standard	quercetin	4	NA	NA
life extension optimized resveratrol with synergistic grape-berry actives 250 mg	Standard	quercetin	4	NA	NA
botanic choice ultra kidney complex	Standard	quercetin	4	NA	NA
the vitamin shoppe enzymes quercetin with bromelain	Standard	bromelain	4	NA	NA
the vitamin shoppe enzymes quercetin with bromelain	Standard	quercetin	4	NA	NA
gnc beyond raw re-built mass super-anabolic mass gainer	Standard	quercetin	4	NA	NA
genesis pure daily build liquid multi-vitamin, mineral and herbal with superfruits	Standard	quercetin dihydrate	4	NA	NA
swanson premium brand high potency quercetin 650 mg	Standard	quercetin	4	NA	NA
d'adamo personalized nutrition genoma nutritionals all types glycoscia	Standard	quercetin	4	NA	NA
trader joe's super vision eye formula	Standard	quercetin powdered extract	3	NA	NA
metagenics multigenics chewable no sucrose added	Infant/pediatric	quercetin	4	NA	NA
total eyebright-m nutri-west	Standard	quercetin	4	NA	NA
purity products ultimate h.a. formula	Standard	quercetin dihydrate	4	NA	NA
reserveage organics the original red wine antioxidant the world's finest resveratrol 100 mg cellular age-defying formula from the heart of france contains trans-resveratrol	Standard	quercetin	4	NA	NA
cvs pharmacy vitamin c 1000 mg tangerine-flavored fizzy drink includes 24 nutrients with	Standard	quercetin	4	NA	NA

7 b vitamins, antioxidants & electrolytes single-serve powder packets					
greens first ceautamed worldwide, llc alkalize now- ph balance program whole food plant based 15+ servings of fruits & vegetables contains: certified organic fruits & vegetables	Standard	quercetin	4	NA	NA
genesis pure daily build liquid multi-vitamin, mineral and herbal with superfruits packets	Standard	quercetin dihydrate	4	NA	NA
true health dr. cutler's advanced artery solution	Standard	quercetin	4	NA	NA
new vitality ruby reds new & improved formula pomegranate, acai & maqui a delicious fruit and vegetable with potent vitamins, minerals, enzymes, herbs, nutrients and probiotics	Standard	quercetin dihydrate / quercetin	4	NA	NA
gnc multivitamin ultra mega gold nutrient-dense multivitamin, mineral and antioxidant formula timed release	Standard	quercetin	4	NA	NA
rejuvenation science advanced multivitamin maximum vitality multi with coq10, vitamin k2, ala & tocotrienols	Standard	quercetin	4	NA	NA
now quercetin with bromelain vegetarian/vegan	Standard	quercetin	4	NA	NA
now quercetin with bromelain vegetarian/vegan	Standard	bromelain (2400 gdu/g)	4	NA	NA
integrative therapeutics vitamin c with quercetin	Standard	total carbohydrate	4	NA	NA
integrative therapeutics vitamin c with quercetin	Standard	vitamin c	1	NA	NA
integrative therapeutics vitamin c with quercetin	Standard	quercetin dihydrate	4	NA	NA
integrative therapeutics vitamin c with quercetin	Standard	citrus bioflavonoids complex	4	NA	NA
metagenics fem essentials for women	Standard	quercetin	4	NA	NA
now clinical strength prostate health clinical strength saw palmetto, beta-sitosterol & lycopene	Standard	quercetin	4	NA	NA
life extension vitamin c with dihydroquercetin 1000 mg	Standard	vitamin c	1	NA	NA
life extension vitamin c with dihydroquercetin 1000 mg	Standard	dihydroquercetin-3-rhamnoside	4	NA	NA

health resources super coq10 plus	Standard	quercetin	4	NA	NA
oregon's wild harvest aller-aid with quercetin and nac	Standard	n-acetyl cysteine (nac)	5	NA	NA
oregon's wild harvest aller-aid with quercetin and nac	Standard	quercetin	4	NA	NA
oregon's wild harvest aller-aid with quercetin and nac	Standard	vitamin c	1	NA	NA
oregon's wild harvest aller-aid with quercetin and nac	Standard	nettle (tops)	3	NA	NA
northstar nutritionals healthy aging	Standard	quercetin	4	NA	NA
nutra biogenesis bioinflamax capsules	Standard	quercetin	4	NA	NA
irwin naturals inflamma-less omega-3 oils, turmeric extract and proteolytic enzymes	Standard	quercetin	4	NA	NA
vitacost synergy once daily multi-vitamin physician formulated	Standard	quercetin	4	NA	NA
doterra mito2max energy & stamina complex	Standard	quercetin	4	NA	NA
viva vitamins csp packs complete supplement program convenient, daily packets with all your essential vitamins and chelated minerals multi vitamin multi mineral complete e complete c regular strength	Standard	quercetin	4	NA	NA
xyngular axion	Standard	quercetin dihydrate	4	NA	NA
usana cellsentials vita-antioxidant	Standard	quercetin dihydrate	4	NA	NA
perricone md nutriceuticals skin & total body packets	Standard	quercetin dihydrate	4	NA	NA
purity products perfect multi super essentials with vision factors	Standard	quercetin	4	NA	NA
real health the prostate formula with saw palmetto advanced formulation now with vitamin d & ginger!	Standard	quercetin	4	NA	NA
emergen-c vitamin d & calcium fizzy drink mix 1000 iu vitamin d and 500 mg calcium 500 mg vitamin c	Standard	quercetin	4	NA	NA
Search for “genistein”					
freelife feeling young premium multi-vitamin & mineral with 100 anti-aging nutrients	Standard	feeling young fruit and vegetable extract complex	NA	genistein	Other
health direct nutrition nature's optimal nutrition	Standard	health direct nature's optimal	NA	soy (genistein)	Botanical

a complete, balanced and high-potency multivitamin, antioxidant		nutrition proprietary exclusive phytonutrient fruit and vegetable blend			
natrol for women soy isoflavones with genistein & daidzein	Standard	isoflavones	4	NA	NA
natrol for women soy isoflavones with genistein & daidzein	Standard	soy isoflavone extract	3	NA	NA
natrol for women soy isoflavones with genistein & daidzein	Standard	daidzin & daidzein	4	NA	NA
natrol for women soy isoflavones with genistein & daidzein	Standard	genistin & genistein	4	NA	NA
natrol for women soy isoflavones with genistein & daidzein	Standard	glycitin & glycitein	4	NA	NA
natrol for women menopause formula	Mature	genistein	4	NA	NA
nature's plus ultra isoflavone 100	Standard	genistein	4	NA	NA
mini's vitamin mineral & herbal formula essentials by megafood full color spectrum foodbased nutrition 72% whole food	Standard	soy 1% isoflavones / genistein	3	NA	NA
purity's perfect multi advanced formula multi-vitamin, mineral, herbal phyto-nutrient super formula without iron	Standard	soy (genistein)	3	NA	NA
nature's bounty non-gmo soy isoflavones with daidzein, genistein and other soy isoflavones	Standard	soy isoflavones	3	NA	NA
nature's bounty non-gmo soy isoflavones with daidzein, genistein and other soy isoflavones	Standard	daidzin & daidzein	4	NA	NA
nature's bounty non-gmo soy isoflavones with daidzein, genistein and other soy isoflavones	Standard	genistin & genistein	4	NA	NA
nature's bounty non-gmo soy isoflavones with daidzein, genistein and other soy isoflavones	Standard	glycitin & glycitein	4	NA	NA
nature's bounty non-gmo soy isoflavones with daidzein, genistein and other soy isoflavones	Standard	soy extract (soy life)	3	NA	NA
nature's bounty non-gmo soy isoflavones with daidzein, genistein and other soy isoflavones	Standard	soy saponins	3	NA	NA
pharmacist's ultimate health super soy 10-12% isoflavones	Standard	genistin & genistein	4	NA	NA
your vitamins from	Standar	total genistein	4	NA	NA

andrew lessman women's wellness with coenzyme q-10	d				
your vitamins andrew lessman bone & body factors calcium-magnesium-phosphorus women's wellness soy isoflavones	Standard	genistein	4	NA	NA
bausch & lomb ocuvite df eye vitamin	Standard	genistein	4	NA	NA
procaps laboratories bone & body factors calcium-magnesium-phosphorus intensive care women's wellness soy	Standard	genistein	4	NA	NA
fosteum capsules	Standard	genistein aglycone	4	NA	NA
citracal calcium + d plus bone density builder with genistein multimineral bayer	Standard	boron	2	NA	NA
citracal calcium + d plus bone density builder with genistein multimineral bayer	Standard	calcium	2	NA	NA
citracal calcium + d plus bone density builder with genistein multimineral bayer	Standard	copper	2	NA	NA
citracal calcium + d plus bone density builder with genistein multimineral bayer	Standard	magnesium	2	NA	NA
citracal calcium + d plus bone density builder with genistein multimineral bayer	Standard	manganese	2	NA	NA
citracal calcium + d plus bone density builder with genistein multimineral bayer	Standard	molybdenum	2	NA	NA
citracal calcium + d plus bone density builder with genistein multimineral bayer	Standard	vitamin d	1	NA	NA
citracal calcium + d plus bone density builder with genistein multimineral bayer	Standard	zinc	2	NA	NA
citracal calcium + d plus bone density builder with genistein multimineral bayer	Standard	genistein	4	NA	NA
pharmanex estera phase iii women's maintenance formula	Standard	genistein	4	NA	NA
cvs/pharmacy one daily bone health + uc-ii for joints for men & women	Standard	genistein	4	NA	NA
women's wellness procaps laboratories	Standard	genistein	4	NA	NA

apex energetics neuro-ptx (k-47) vitamin, amino acid, & herbal	Standard	genistein	4	NA	NA
apex energetics estrovite (k-5) multivitamin, mineral, & herbal	Standard	genistein	4	NA	NA
i-cool hormone-free	Standard	genivida non-soy genistein	4	NA	NA
Search for “glycitein”					
natrol for women soy isoflavones with genistein & daidzein	Standard	glycitin & glycitein	4	NA	NA
nature's plus ultra isoflavone 100	Standard	glycitein	4	NA	NA
nature's bounty non-gmo soy isoflavones with daidzein, genistein and other soy isoflavones	Standard	glycitin & glycitein	4	NA	NA
pharmacist's ultimate health super soy 10-12% isoflavones	Standard	glycitin & glycitein	4	NA	NA
Search for “epicatechin”					
limbrel 500 flavocoxid and citrated zinc bisglycinate 500 mg/50 mg	Standard	limbrel flavocoxid proprietary blend	NA	epicatechin	Other
Search for “epigallocatechin”					
nutrilite concentrated fruits and vegetables phytonutrient	Standard	egcg (epigallocatechin gallate)	4	NA	NA
natrol acaiberry diet acai & green tea super foods	Standard	egcg (epigallocatechin gallate)	4	NA	NA
natrol carb intercept 3 1 - 2 - 3 lean phase 2 clinically validated white kidney bean extract	Standard	egcg (epigallocatechin gallate)	4	NA	NA
breast health phytonutrient anti-oxidants indole-3-carbinol sulforaphane procaps laboratories	Standard	egcg (epigallocatechin gallate)	4	NA	NA
women's wellness procaps laboratories	Standard	egcg (epigallocatechin gallate)	4	NA	NA
ultimate anti-oxidant extracts world health, spice of life, fruit, berry & cruciferous extracts procaps laboratories	Standard	egcg (epigallocatechin gallate)	4	NA	NA
rexall sundown naturalist one daily energy multivitamin with green tea extract (egcg)	Standard	egcg (epigallocatechin gallate)	4	NA	NA
swanson ultra egcg super-strength green tea 275 mg	Standard	egcg (epigallocatechin gallate)	4	NA	NA
walgreens green tea with egcg (green tea extract)	Standard	egcg (epigallocatechin gallate)	4	NA	NA
Search for “epicatechin”					
nutrilite concentrated	Standar	egcg	4	NA	NA

fruits and vegetables phytonutrient	d	(epigallocatechin gallate)			
natrol acaiberry diet acai & green tea super foods	Standar d	egcg (epigallocatechin gallate)	4	NA	NA
natrol carb intercept 3 1 - 2 - 3 lean phase 2 clinically validated white kidney bean extract	Standar d	egcg (epigallocatechin gallate)	4	NA	NA
breast health phytonutrient anti-oxidants indole-3-carbinol sulforaphane procaps laboratories	Standar d	egcg (epigallocatechin gallate)	4	NA	NA
women's wellness procaps laboratories	Standar d	egcg (epigallocatechin gallate)	4	NA	NA
ultimate anti-oxidant extracts world health, spice of life, fruit, berry & cruciferous extracts procaps laboratories	Standar d	egcg (epigallocatechin gallate)	4	NA	NA
rexall sundown naturalist one daily energy multivitamin with green tea extract (egcg)	Standar d	egcg (epigallocatechin gallate)	4	NA	NA
swanson ultra egcg super-strength green tea 275 mg	Standar d	egcg (epigallocatechin gallate)	4	NA	NA
walgreens green tea with egcg (green tea extract)	Standar d	egcg (epigallocatechin gallate)	4	NA	NA
Search for "anthocyanin"					
vital basics focus factor adult formula	Standar d	synergistic and proprietary formulation	NA	bilberry 25% anthocyanins	Botanical
juice plus+ orchard blend chewables	Standar d	juice plus+ orchard proprietary blend	NA	anthocyanins	Other
juice plus+ garden blend chewables	Standar d	juice plus+ garden proprietary blend	NA	anthocyanins	Other
garden greens acaislim thermogenic green tea, fucoxanthin and chromium polyphenols and anthocyanins from acai fruit	Standar d	garden greens acaislim thermogenic blend	NA	caffeine	Other
garden greens acaislim thermogenic green tea, fucoxanthin and chromium polyphenols and anthocyanins from acai fruit	Standar d	garden greens acaislim thermogenic blend	NA	green tea leaf extract	Botanical
garden greens acaislim thermogenic green tea, fucoxanthin and chromium polyphenols and anthocyanins from acai fruit	Standar d	garden greens acaislim thermogenic blend	NA	panax ginseng extract	Botanical
garden greens acaislim thermogenic green tea, fucoxanthin and chromium polyphenols	Standar d	garden greens acaislim thermogenic blend	NA	cayenne pepper	Botanical

and anthocyanins from acai fruit					
garden greens acaislim thermogenic green tea, fucoxanthin and chromium polyphenols and anthocyanins from acai fruit	Standard	garden greens acaislim thermogenic blend	NA	acai fruit extract	Botanical
garden greens acaislim thermogenic green tea, fucoxanthin and chromium polyphenols and anthocyanins from acai fruit	Standard	garden greens acaislim thermogenic blend	NA	fucoxanthin	Other
lifepak	Standard	grape seed leucoanthocyanin extract	3	NA	NA
lifepak prime	Standard	grape seed leucoanthocyanin extract	3	NA	NA
lifepak women	Standard	grape seed leucoanthocyanin extract	3	NA	NA
lifepak trim	Standard	grape seed leucoanthocyanin extract	3	NA	NA
enzymatic therapy doctor's choice for diabetics	Standard	bilberry 25% anthocyanins	3	NA	NA
bally total fitness high potency multi-vitamin plus mineral formula with herbs men	Standard	grape seed leucoanthocyanin extract	3	NA	NA
bally total fitness high potency multi-vitamin plus mineral formula with herbs women	Standard	grape seed leucoanthocyanin extract	3	NA	NA
body design overdrive pharmanex	Standard	grape seed leucoanthocyanin extract	3	NA	NA
bally total fitness high potency multivitamin plus men with saw palmetto & lycopene	Standard	grape seed leucoanthocyanin extract	3	NA	NA
garden greens acaislim thermogenic green tea, fucoxanthin and chromium polyphenols and anthocyanins from acai fruit	Standard	chromium	2	NA	NA
life extension mix capsules state-of-the-art multi-nutrient formula	Standard	wild blueberry fruit anthocyanin extract	3	NA	NA
life extension mix capsules state-of-the-art multi-nutrient formula	Standard	maqui berry fruit anthocyanin extract	3	NA	NA
life extension mix capsules state-of-the-art multi-nutrient formula	Standard	tart cherry proanthocyanin extract (skin & pulp)	3	NA	NA
life extension mix capsules state-of-the-art multi-nutrient formula	Standard	biovin grape proanthocyanin extract (whole)	3	NA	NA

life extension mix capsules state-of-the-art multi-nutrient formula	Standard	leucoselect grape seed proanthocyanin extract (seed)	3	NA	NA
life extension life extension mix tablets state-of-the-art multi-nutrient formula	Standard	wild blueberry fruit anthocyanin extract	3	NA	NA
life extension life extension mix tablets state-of-the-art multi-nutrient formula	Standard	maqui berry fruit anthocyanin extract	3	NA	NA
swanson superior herbs bilberry extract standardized 25% anthocyanins 60 mg	Standard	bilberry extract (fruit)	3	NA	NA
ultimate anti-oxidant extracts world health, spice of life, fruit, berry & cruciferous extracts procaps laboratories	Standard	acai berry extract standardized to 4.5% polyphenols / 1% anthocyanins	3	NA	NA
ultimate anti-oxidant extracts world health, spice of life, fruit, berry & cruciferous extracts procaps laboratories	Standard	ultimate anti-oxidant extracts bilberry blend, extract fruit 25% / 10% anthocyanins	3	NA	NA
Search for “flavanone”					
twinlab citrus bioflavonoid caps with rutin	Standard	twinlab citrus bioflavonoid caps citrus bioflavonoids complex	NA	flavanones	Other
bluebonnet c-1000 plus bioflavonoids vitamin c plus citrus bioflavonoids	Standard	flavanones	4	NA	NA
Search for “flavone”					
nature's way alive! whole food energizer with iron veggie cap	Standard	alive! citrus bioflavonoid complex	NA	flavones and related phenolic compounds	Other
nature's way alive! whole food energizer (iron free) veggie cap	Standard	alive! citrus bioflavonoid complex	NA	flavones and related phenolic compounds	Other
newphase phytoestrogen support for women sunsource	Mature	newphase multi-herbal isoflavone blend	NA	soy protein concentrate	Botanical
newphase complete all-natural drug free	Mature	newphase multi-herbal isoflavone blend	NA	soy protein concentrate	Botanical
newphase phytoestrogen support for women sunsource	Mature	newphase multi-herbal isoflavone blend	NA	red clover extract	Botanical
newphase complete all-natural drug free	Mature	newphase multi-herbal isoflavone blend	NA	red clover extract	Botanical
newphase phytoestrogen support for women sunsource	Mature	newphase multi-herbal isoflavone blend	NA	kudzu extract (root)	Botanical
newphase complete all-natural drug free	Mature	newphase multi-herbal isoflavone blend	NA	kudzu extract (root)	Botanical
free life msm ultra caplets	Standard	free life citri-c complex	NA	heptamethoxyflavone	Other
xenadrine-efx	Standar	xenadrine-efx	NA	3,3,4,7-	Other

	d	thermodyne complex		tetrahydroxyflavone	
xenadrine-efx	Standard	xenadrine-efx thermodyne complex	NA	3,3,4,5-7 pentahydroxyflavone	Other
ladies choice	Standard	ladies choice proprietary blend	NA	soy isoflavones	Botanical
nikken for women with isoflavone plus complex	Standard	nikken isoflavone plus complex	NA	n-acetyl l-cysteine	Amino acid
nikken for women with isoflavone plus complex	Standard	nikken isoflavone plus complex	NA	soy isoflavones	Botanical
nikken for women with isoflavone plus complex	Standard	nikken isoflavone plus complex	NA	dong quai (root)	Botanical
nikken for women with isoflavone plus complex	Standard	nikken isoflavone plus complex	NA	black cohosh (root)	Botanical
nikken for women with isoflavone plus complex	Standard	nikken isoflavone plus complex	NA	fenugreek (whole plant)	Botanical
nuhair hair regrowth tablets for men 100% natural	Standard	nuhair follicle stimulating botanical blend	NA	isoflavones	Other
super juice daily multi phyto-nutrient formula vegetable, fruit & botanical now with botanical antioxidants	Standard	super juice isoflavone concentrate blend	NA	soybean	Botanical
super juice daily multi phyto-nutrient formula vegetable, fruit & botanical now with botanical antioxidants! supplies whole food nutrition from six daily servings!	Standard	super juice isoflavone concentrate blend	NA	soybean	Botanical
super juice daily multi phyto-nutrient formula vegetable, fruit & botanical now with botanical antioxidants	Standard	super juice isoflavone concentrate blend	NA	kudzu root	Botanical
super juice daily multi phyto-nutrient formula vegetable, fruit & botanical now with botanical antioxidants! supplies whole food nutrition from six daily servings!	Standard	super juice isoflavone concentrate blend	NA	kudzu root	Botanical
cytodyne xenadrine-efx extra strength formula ephedrine free with norambrolide	Standard	xenadrine-efx thermodyne complex #2	NA	3,3,4,7-tetrahydroxyflavone	Other
cytodyne xenadrine-efx extra strength formula ephedrine free with norambrolide	Standard	xenadrine-efx thermodyne complex #2	NA	3,3,4,5-7 pentahydroxyflavone	Other
vitality mineral complex with calcium patented fructose compounding	Standard	vitality mineral complex proprietary blend	NA	soy isoflavones	Botanical
vitamin world xtreme trim ephedra free	Standard	vitamin world xtreme trim thermo complex	NA	3,3,4,7-tetrahydroxyflavone	Other
vitamin world xtreme trim ephedra free	Standard	vitamin world xtreme trim thermo complex	NA	3,3,4,5-7 pentahydroxyflavone	Other

reliv soysentials women's daily protective	Standard	reliv soysentials protective proprietary blend	NA	ipriflavone	Other
advanced formula xtreme lean ephedra free	Standard	xtreme lean ephedra free proprietary thermo complex	NA	3,3,4,7-tetrahydroxyflavone	Other
advanced formula xtreme lean ephedra free	Standard	xtreme lean ephedra free proprietary thermo complex	NA	3,3,4,5-7 pentahydroxyflavone	Other
your vitamins andrew lessman circulation & vein support bioflavonoid complex diosmin hesperidin quercetin rutin	Standard	your vitamins flavonol complex	NA	flavones	Other
ritestart women all-in-one nutrition for women with 4life transfer factor plus exclusive anti-aging formula 4life packet	Standard	4life women's health blend	NA	soy isoflavones	Botanical
ritestart women all-in-one nutrition for women with 4life transfer factor plus exclusive anti-aging formula 4life packet	Standard	4life women's health blend	NA	ipriflavone	Other
ritestart men all-in -one nutrition for men with 4life transfer factor plus advanced formula exclusive anti-aging formul	Standard	4life men's health blend	NA	soy isoflavones	Botanical
newphase complete all-natural drug free new improved formula	Mature	newphase multi-herbal isoflavone blend #2	NA	soybean	Botanical
newphase complete all-natural drug free new improved formula	Mature	newphase multi-herbal isoflavone blend #2	NA	kudzu extract (root)	Botanical
newphase complete all-natural drug free new improved formula	Mature	newphase multi-herbal isoflavone blend #2	NA	red clover extract (leaf)	Botanical
equate estroplus extra strength	Standard	equate estroplus isoflavones blend	NA	soy extract (bean)	Botanical
equate estroplus extra strength	Standard	equate estroplus isoflavones blend	NA	kudzu extract (root)	Botanical
equate estroplus extra strength	Standard	equate estroplus isoflavones blend	NA	plant enzymes	Other
sentia	Standard	sentia proprietary blend	NA	isoflavones	Other
one source ultimate women's premium multivitamin designed for women vitamin d 1000 iu calcium 500 mg per serving	Standard	one source ultimate women's antioxidant fruit and vegetable blend	NA	soy isoflavone extract	Botanical
one source ultimate women's multi vitamin d3 1000 iu per serving	Standard	one source ultimate women's antioxidant fruit and vegetable blend	NA	soy isoflavone extract	Botanical
vitality gold for men am / pm high potency	Standard	vitality gold for men am / pm proprietary blend	NA	soy isoflavones	Botanical

nature's plus source of life vitamin, mineral & protein energy shake with whole food concentrates	Standard	nature's plus source of life energy shake lemon bioflavonoid complex (citrus)	NA	flavones	Other
natural balance ladies choice phytoestrogen formula	Standard	natural balance ladies choice proprietary blend	NA	soy isoflavones	Botanical
valerie saxion's silver creek labs, ltd. smart focus	Standard	valerie saxion's silver creek labs, ltd. smart focus proprietary blend	NA	flavone glycosides	Other
cvs pharmacy menopause support extra strength	Standard	cvs pharmacy menopause support isoflavones blend	NA	soy extract (bean)	Botanical
cvs pharmacy menopause support extra strength	Standard	cvs pharmacy menopause support isoflavones blend	NA	kudzu extract (root)	Botanical
daily for life for women am/pm high potency packets	Standard	daily for life for women proprietary blend #1	NA	soy isoflavones	Botanical
member's mark estro vital nutrients extra strength 2 caplets daily	Mature	member's mark estro vital nutrients isoflavones blend	NA	soy extract (bean)	Botanical
member's mark estro vital nutrients extra strength 2 caplets daily	Mature	member's mark estro vital nutrients isoflavones blend	NA	kudzu extract (root)	Botanical
member's mark estro vital nutrients extra strength 2 caplets daily	Mature	member's mark estro vital nutrients isoflavones blend	NA	plant enzymes	Other
mannatech optimal health system glycentials antioxidant formula complete vitamin & mineral vitamin, ambroglycin mineral	Standard	phytonutrient complex 2 (pnc)	NA	isoflavones	Other
nature's way alive! whole food energizer men's multi max potency vitamins & minerals 26 fruits & vegetables green foods enzymes mushrooms amino acids antioxidants lutein resveratrol	Standard	nature's way alive! citrus bioflavonoid complex #3	NA	flavones	Other
nature's way alive! once daily men's ultra potency	Standard	nature's way alive! citrus bioflavonoid complex #3	NA	flavones	Other
nature's way alive! once daily women's ultra potency	Standard	nature's way alive! citrus bioflavonoid complex #3	NA	flavones	Other
nature's way alive! whole food energizer multi-vitamin max potency vitamins & minerals 26 fruits & vegetables green foods enzymes mushrooms amino acids antioxidants lutein resveratrol	Standard	nature's way alive! citrus bioflavonoid complex #3	NA	flavones	Other

nature's way alive! once daily multi-vitamin ultra potency whole food energizer 26 fruits & vegetables green foods mushrooms enzymes antioxidants lutein resveratrol	Standard	nature's way alive! citrus bioflavonoid complex #3	NA	flavones	Other
nature's way alive! whole food energizer women's multi max potency	Standard	nature's way alive! citrus bioflavonoid complex #3	NA	flavones	Other
nature's way alive! once daily men's ultra potency multi-vitamin & whole food energizer	Standard	nature's way alive! citrus bioflavonoid complex #3	NA	flavones	Other
nature's way alive! whole food energizer men's multi max potency	Standard	nature's way alive! citrus bioflavonoid complex #3	NA	flavones	Other
nature's way alive! once daily women's ultra potency multi-vitamin & whole food energizer	Standard	nature's way alive! citrus bioflavonoid complex #3	NA	flavones	Other
nature's way alive! once daily multi-vitamin ultra potency energizer with food based blends orchard fruits / garden veggies & daily greens powder (120 mg), enzymes, bioflavonoids multi-vitamin	Standard	nature's way alive! citrus bioflavonoid complex #3	NA	flavones	Other
ultra woman daily multi premium performance formula for women high potency timed release	Standard	ultra woman bone blend	NA	soy isoflavones	Botanical
ultra woman daily multi premium performance formula for women high potency timed release	Standard	ultra woman bone blend	NA	ipriflavone	Other
onesource multivitamin women's with vitamin d3 4000 iu per serving	Standard	onesource multivitamin women's proprietary antioxidant fruit and vegetable blend	NA	soy isoflavone extract	Botanical
twinlab citrus bioflavonoid caps with rutin	Standard	twinlab citrus bioflavonoid caps citrus bioflavonoids complex	NA	flavones	Other
rite aid pharmacy extra strength menopause support soy, cranberry and calcium complex formula	Standard	rite aid pharmacy extra strength menopause support isoflavone blend	NA	soy isoflavones	Botanical
rite aid pharmacy extra strength menopause support soy, cranberry and calcium complex formula	Standard	rite aid pharmacy extra strength menopause support isoflavone blend	NA	kudzu root	Botanical
nature's way alive! liquid multi-vitamin max	Standard	nature's way alive! citrus bioflavonoid	NA	flavones	Other

potency fruits, veggies, green foods, antioxidants, amino acids, efas, herbs, mushrooms, lutein & more! vegetarian formula		complex #4			
nature's way alive! multi-vitamin max potency liquid multi with food-based blends orchard fruits garden veggies daily greens blends (700 mg) bioflavonoids efas mushrooms herbs vegetarian formula	Standard	nature's way alive! citrus bioflavonoid complex #4	NA	flavones	Other
pure essence labs one 'n' only women uniquely feminine formula multivitamin & mineral 23,700 mg whole food value	Standard	pure essence labs one 'n' only female support factors	NA	ipriflavone	Other
soy care bone health	Standard	soy isoflavones	3	NA	NA
lifepak	Standard	isoflavones	4	NA	NA
lifepak prime	Standard	isoflavones	4	NA	NA
lifepak women	Standard	isoflavones	4	NA	NA
lifepak trim	Standard	isoflavones	4	NA	NA
nature made ginkgo biloba 40 mg standardized extract	Standard	flavone glycosides	4	NA	NA
gnc women's menopause vita pak	Mature	soy isoflavones	3	NA	NA
caltrate 600 + soy with soy isoflavones	Standard	calcium	2	NA	NA
caltrate 600 + soy with soy isoflavones	Standard	vitamin d	1	NA	NA
caltrate 600 + soy with soy isoflavones	Standard	soy isoflavones	3	NA	NA
natrol for women soy isoflavones with genistein & daidzein	Standard	isoflavones	4	NA	NA
natrol for women soy isoflavones with genistein & daidzein	Standard	soy isoflavone extract	3	NA	NA
natrol for women soy isoflavones with genistein & daidzein	Standard	daidzin & daidzein	4	NA	NA
natrol for women soy isoflavones with genistein & daidzein	Standard	genistin & genistein	4	NA	NA
natrol for women soy isoflavones with genistein & daidzein	Standard	glycitin & glycitein	4	NA	NA
natrol for women menopause formula	Mature	soy isoflavones	3	NA	NA
gnc herbal rush	Standard	soy isoflavone concentrate	3	NA	NA
your life calcium 500 mg with vitamin d 200 iu plus soy 15mg contains	Standard	isoflavones	4	NA	NA

novasoy					
women's formula advanced breast health formula with patented calcium d-glucarate	Standard	isoflavones	4	NA	NA
nature made essential multi plus memory multivitamin with standardized ginkgo biloba extract	Standard	flavone glycosides	4	NA	NA
jarrow formulas ultra bone-up	Standard	ipriflavone	4	NA	NA
free life osteosoy	Standard	isoflavones	4	NA	NA
gnc women's menopause vita pak	Mature	soy isoflavones	3	NA	NA
walgreens finest ginkgo-memo concentrate 40 mg	Standard	flavone glycosides	4	NA	NA
bluebonnet c-1000 plus bioflavonoids vitamin c plus citrus bioflavonoids	Standard	flavones	4	NA	NA
nature's resource standardized extract ginkgo biloba 30 mg	Standard	flavone glycosides	4	NA	NA
nature's resource ginkgo biloba 60 mg standardized extract	Standard	flavone glycosides	4	NA	NA
gnc natural brand soy isoflavone concentrate 50 mg	Standard	soy isoflavone concentrate	3	NA	NA
solar green phytoestrogen greens	Standard	isoflavones	4	NA	NA
nature's plus ultra isoflavone 100	Standard	isoflavones	4	NA	NA
nature's plus ultra isoflavone 100	Standard	genistein	4	NA	NA
nature's plus ultra isoflavone 100	Standard	puerarin	4	NA	NA
nature's plus ultra isoflavone 100	Standard	daidzin	4	NA	NA
nature's plus ultra isoflavone 100	Standard	daidzein	4	NA	NA
nature's plus ultra isoflavone 100	Standard	glycitin	4	NA	NA
nature's plus ultra isoflavone 100	Standard	genistin	4	NA	NA
nature's plus ultra isoflavone 100	Standard	glycitein	4	NA	NA
vitamin world time release mega vita min for women high potency	Standard	soy isoflavones	3	NA	NA
vitamin world time release mega vita min for women high potency	Standard	ipriflavone	4	NA	NA
health from the sun fermented soy essentials whole food 800 mg	Standard	isoflavones	4	NA	NA
estroven soy and black cohosh plus vitamins b-6, e & calcium	Mature	isoflavones	4	NA	NA
nature's way standardized soy isoflavone extract	Standard	soy isoflavone extract	3	NA	NA
nature's way standardized	Standar	red clover flower	3	NA	NA

soy isoflavone extract	d				
sundown soy isoflavones	Standard	calcium	2	NA	NA
sundown soy isoflavones	Standard	soy isoflavone concentrate	3	NA	NA
vitadvance bone health with soy isoflavones	Standard	boron	2	NA	NA
vitadvance bone health with soy isoflavones	Standard	calcium	2	NA	NA
vitadvance bone health with soy isoflavones	Standard	copper	2	NA	NA
vitadvance bone health with soy isoflavones	Standard	magnesium	2	NA	NA
vitadvance bone health with soy isoflavones	Standard	manganese	2	NA	NA
vitadvance bone health with soy isoflavones	Standard	vitamin d	1	NA	NA
vitadvance bone health with soy isoflavones	Standard	vitamin k	1	NA	NA
vitadvance bone health with soy isoflavones	Standard	zinc	2	NA	NA
vitadvance bone health with soy isoflavones	Standard	soy isoflavones	3	NA	NA
vitadvance bone health with soy isoflavones	Standard	vitamin c	1	NA	NA
vitadvance bone health with soy isoflavones	Standard	vitamin b-6	1	NA	NA
vitadvance menopause support with black cohosh & soy	Mature	soy isoflavones	3	NA	NA
generic calcium 600 + soy with soy isoflavones	Standard	calcium	2	NA	NA
generic calcium 600 + soy with soy isoflavones	Standard	vitamin d	1	NA	NA
generic calcium 600 + soy with soy isoflavones	Standard	soy isoflavones	3	NA	NA
mini's vitamin mineral & herbal formula essentials by megafood full color spectrum foodbased nutrition 72% whole food	Standard	soy 1% isoflavones / genistein	3	NA	NA
anabolic laboratories bone support formula premium calcium complex with soy isoflavones & support nutrients	Standard	boron	2	NA	NA
anabolic laboratories bone support formula premium calcium complex with soy isoflavones & support nutrients	Standard	calcium	2	NA	NA
anabolic laboratories bone support formula premium calcium complex with soy isoflavones & support nutrients	Standard	copper	2	NA	NA
anabolic laboratories bone support formula premium calcium complex with soy	Standard	magnesium	2	NA	NA

isoflavones & support nutrients					
anabolic laboratories bone support formula premium calcium complex with soy isoflavones & support nutrients	Standard	manganese	2	NA	NA
anabolic laboratories bone support formula premium calcium complex with soy isoflavones & support nutrients	Standard	silicon	2	NA	NA
anabolic laboratories bone support formula premium calcium complex with soy isoflavones & support nutrients	Standard	vitamin d	1	NA	NA
anabolic laboratories bone support formula premium calcium complex with soy isoflavones & support nutrients	Standard	vitamin k	1	NA	NA
anabolic laboratories bone support formula premium calcium complex with soy isoflavones & support nutrients	Standard	zinc	2	NA	NA
anabolic laboratories bone support formula premium calcium complex with soy isoflavones & support nutrients	Standard	vitamin c	1	NA	NA
anabolic laboratories bone support formula premium calcium complex with soy isoflavones & support nutrients	Standard	novasoy isoflavone complex	4	NA	NA
anabolic laboratories bone support formula premium calcium complex with soy isoflavones & support nutrients	Standard	bioflavonoid complex	4	NA	NA
ginkgo biloba 24% ginkgo flavone glycosides nf formulas, inc.	Standard	ginkgo biloba leaf	3	NA	NA
ginkgo biloba 24% ginkgo flavone glycosides nf formulas, inc.	Standard	g. biloba leaf- 24% ginkgo flavone glycosides 6% terpene lactones	3	NA	NA
nature's bounty non-gmo soy isoflavones with daidzein, genistein and other soy isoflavones	Standard	soy isoflavones	3	NA	NA
nature's bounty non-gmo soy isoflavones with	Standard	daidzin & daidzein	4	NA	NA

daidzein, genistein and other soy isoflavones					
nature's bounty non-gmo soy isoflavones with daidzein, genistein and other soy isoflavones	Standard	genistin & genistein	4	NA	NA
nature's bounty non-gmo soy isoflavones with daidzein, genistein and other soy isoflavones	Standard	glycitin & glycitein	4	NA	NA
nature's bounty non-gmo soy isoflavones with daidzein, genistein and other soy isoflavones	Standard	soy extract (soy life)	3	NA	NA
nature's bounty non-gmo soy isoflavones with daidzein, genistein and other soy isoflavones	Standard	soy saponins	3	NA	NA
longs standardized herbal extract ginkgo biloba 60 mg concentrate	Standard	ginkgo biloba extract (leaf, 24% ginkgo flavone glycosides & 6% terpene lactones)	3	NA	NA
prescriptive formulas women's optimal vitamin packs complete daily nutritional program	Standard	soy isoflavones	3	NA	NA
nikken for women with isoflavone plus complex	Standard	calcium	2	NA	NA
nikken for women with isoflavone plus complex	Standard	grape seed extract	3	NA	NA
nikken for women with isoflavone plus complex	Standard	iron	2	NA	NA
nikken for women with isoflavone plus complex	Standard	lutein	4	NA	NA
nikken for women with isoflavone plus complex	Standard	magnesium	2	NA	NA
nikken for women with isoflavone plus complex	Standard	vitamin d	1	NA	NA
nikken for women with isoflavone plus complex	Standard	vitamin e	1	NA	NA
nikken for women with isoflavone plus complex	Standard	melatonin	4	NA	NA
nikken for women with isoflavone plus complex	Standard	vitamin c	1	NA	NA
nikken for women with isoflavone plus complex	Standard	vitamin b-6	1	NA	NA
nikken for women with isoflavone plus complex	Standard	vitamin b-12	1	NA	NA
nikken for women with isoflavone plus complex	Standard	folic acid	1	NA	NA
nikken for women with isoflavone plus complex	Standard	cranberry fruit powder	3	NA	NA
default soy isoflavones	Standard	calcium	2	NA	NA
default soy isoflavones	Standard	soy isoflavone concentrate	3	NA	NA
healthy woman soy supplement 55 mg of soy isoflavones	Standard	calories	4	NA	NA
healthy woman soy supplement 55 mg of soy isoflavones	Standard	soy standardized extract	3	NA	NA

healthy woman soy supplement 55 mg of soy isoflavones	Standard	isoflavones (genistin/daidzin)	3	NA	NA
twinlab truherbs time release ginkgo standardized extract 490 mg	Standard	ginkgo biloba extract (leaf, 24% ginkgo flavone glycosides & 6% terpene lactones)	3	NA	NA
tabak's health products the mega wellness system	Standard	soy isoflavone extract	3	NA	NA
pharmanex lifepak new anti-aging formula	Standard	isoflavones	4	NA	NA
pharmanex lifepak new anti-aging formula	Standard	isoflavones	4	NA	NA
pharmacist's ultimate health super soy 10-12% isoflavones	Standard	soy standardized extract	3	NA	NA
pharmacist's ultimate health super soy 10-12% isoflavones	Standard	daidzin & daidzein	4	NA	NA
pharmacist's ultimate health super soy 10-12% isoflavones	Standard	genistin & genistein	4	NA	NA
pharmacist's ultimate health super soy 10-12% isoflavones	Standard	glycitin & glycitein	4	NA	NA
your life ginkgo biloba standardized herbal extract 60 mg concentrate	Standard	ginkgo biloba extract (leaf, 24% ginkgo flavone glycosides & 6% terpene lactones)	3	NA	NA
your life complete spectra multivitamin plus herbs with lutein, bilberry, vitamins a and c vitamins, minerals, and herbs	Standard	isoflavones	4	NA	NA
your life complete spectra multivitamin plus herbs with lutein, bilberry, vitamins a and c vitamins, minerals, and herbs	Standard	ginkgo biloba extract (leaf, 24% ginkgo flavone glycosides & 6% terpene lactones)	3	NA	NA
estroven extra strength	Standard	isoflavones	4	NA	NA
estroven bone density calcium with magnesium, vitamins d and k, and soy isoflavones	Standard	calcium	2	NA	NA
estroven bone density calcium with magnesium, vitamins d and k, and soy isoflavones	Standard	magnesium	2	NA	NA
estroven bone density calcium with magnesium, vitamins d and k, and soy isoflavones	Standard	vitamin d	1	NA	NA
estroven bone density calcium with magnesium, vitamins d and k, and soy isoflavones	Standard	vitamin k	1	NA	NA
estroven bone density calcium with magnesium, vitamins d and k, and soy isoflavones	Standard	isoflavones	4	NA	NA

estroven bone density calcium with magnesium, vitamins d and k, and soy isoflavones	Standard	citrus bioflavonoid complex	3	NA	NA
default citrus bioflavonoids	Standard	flavones	4	NA	NA
gnc women's soy isoflavones	Standard	soy isoflavone extract	3	NA	NA
natrol complete balance am pm for menopause am formula	Standard	soy isoflavones	3	NA	NA
natrol complete balance am pm for menopause pm formula	Standard	soy isoflavones	3	NA	NA
swanson health products premium brand ipriflavone complex with ostivone	Standard	boron	2	NA	NA
swanson health products premium brand ipriflavone complex with ostivone	Standard	calcium	2	NA	NA
swanson health products premium brand ipriflavone complex with ostivone	Standard	vitamin d	1	NA	NA
swanson health products premium brand ipriflavone complex with ostivone	Standard	ostivone (ipriflavone)	4	NA	NA
nature's blend super antioxidant with lutein and lycopene contains novasoy 20 mg standardized concentrated extract	Standard	novasoy soy isoflavones	4	NA	NA
pathway to health reginald b. cherry, m.d. prostate support	Standard	soy isoflavones	3	NA	NA
nature's resource soy balance menopause soy 65 mg soy isoflavones	Standard	soy standardized extract	3	NA	NA
nature's resource soy balance menopause soy 65 mg soy isoflavones	Standard	soy isoflavones	3	NA	NA
womax women's formula soy isoflavone herbal complex natural phytoestrogen isoflavones maxi-health research, inc.	Standard	black cohosh root extract	3	NA	NA
womax women's formula soy isoflavone herbal complex natural phytoestrogen isoflavones maxi-health research, inc.	Standard	dong quai root extract 4:1	3	NA	NA
womax women's formula soy isoflavone herbal complex natural phytoestrogen isoflavones maxi-health research, inc.	Standard	chasteberry powder	3	NA	NA

womax women's formula soy isoflavone herbal complex natural phytoestrogen isoflavones maxi-health research, inc.	Standard	soy extract powder	3	NA	NA
puritan's pride ginkgo biloba 60 mg standardized extract standardized to contain 24% ginkgo flavone glycosides	Standard	ginkgo biloba extract (leaf)	3	NA	NA
curves protein drink vanilla flavored	Standard	soy isoflavone concentrate	3	NA	NA
natrol ginkgo biloba 60 mg	Standard	flavone glycosides	4	NA	NA
trunature soy isoflavones standardized concentrated herbal extract 50 mg	Standard	soy extract (bean)	3	NA	NA
generic8 ginkgo biloba 60 mg	Standard	ginkgo biloba extract (leaf, 24% ginkgo flavone glycosides & 6% terpene lactones)	3	NA	NA
sundown high potency time release ultra woman vitamin, mineral, herb formula for women	Standard	soy isoflavones	3	NA	NA
sundown high potency time release ultra woman vitamin, mineral, herb formula for women	Standard	ipriflavone	4	NA	NA
default calcium + soy	Standard	soy isoflavones	3	NA	NA
your vitamins from andrew lessman women's wellness with coenzyme q-10	Standard	total isoflavones	4	NA	NA
generic soy and black cohosh with vitamins b-6, e & calcium	Mature	isoflavones	4	NA	NA
gnc men's timed release senior formula	Mature	soy isoflavone extract	3	NA	NA
vitamin world time release mega vita min for women high potency	Standard	soy isoflavones	3	NA	NA
puritan's pride ginkgo biloba 30 mg standardized extract standardized to contain 24% ginkgo flavone glycosides	Standard	ginkgo biloba extract	3	NA	NA
walgreens estronatural	Mature	isoflavones	4	NA	NA
youngevity anti-aging daily premium pak	Standard	soy isoflavones	3	NA	NA
reliv soysentials women's daily protective	Standard	soy isoflavones	3	NA	NA
your vitamins andrew lessman circulation & vein support bioflavonoid complex diosmin hesperidin quercetin rutin	Standard	flavones	4	NA	NA

your vitamins andrew lessman bone & body factors calcium-magnesium-phosphorus women's wellness soy isoflavones	Standard	boron	2	NA	NA
your vitamins andrew lessman bone & body factors calcium-magnesium-phosphorus women's wellness soy isoflavones	Standard	calcium	2	NA	NA
your vitamins andrew lessman bone & body factors calcium-magnesium-phosphorus women's wellness soy isoflavones	Standard	chinese green tea extract	3	NA	NA
your vitamins andrew lessman bone & body factors calcium-magnesium-phosphorus women's wellness soy isoflavones	Standard	lutein	4	NA	NA
your vitamins andrew lessman bone & body factors calcium-magnesium-phosphorus women's wellness soy isoflavones	Standard	magnesium	2	NA	NA
your vitamins andrew lessman bone & body factors calcium-magnesium-phosphorus women's wellness soy isoflavones	Standard	phosphorus	2	NA	NA
your vitamins andrew lessman bone & body factors calcium-magnesium-phosphorus women's wellness soy isoflavones	Standard	silicon	2	NA	NA
your vitamins andrew lessman bone & body factors calcium-magnesium-phosphorus women's wellness soy isoflavones	Standard	vitamin d	1	NA	NA
your vitamins andrew lessman bone & body factors calcium-magnesium-phosphorus women's wellness soy isoflavones	Standard	isoflavones	4	NA	NA
your vitamins andrew lessman bone & body factors calcium-magnesium-phosphorus women's wellness soy isoflavones	Standard	cranberry fruit extract	3	NA	NA
your vitamins andrew lessman bone & body factors calcium-	Standard	lycopene	4	NA	NA

magnesium-phosphorus women's wellness soy isoflavones					
your vitamins andrew lessman bone & body factors calcium-magnesium-phosphorus women's wellness soy isoflavones	Standard	genistein	4	NA	NA
your vitamins andrew lessman bone & body factors calcium-magnesium-phosphorus women's wellness soy isoflavones	Standard	daidzein	4	NA	NA
your vitamins andrew lessman bone & body factors calcium-magnesium-phosphorus women's wellness soy isoflavones	Standard	vitamin b-12	1	NA	NA
your vitamins andrew lessman bone & body factors calcium-magnesium-phosphorus women's wellness soy isoflavones	Standard	folic acid	1	NA	NA
your vitamins andrew lessman bone & body factors calcium-magnesium-phosphorus women's wellness soy isoflavones	Standard	egcg (from green tea extract leaf)	3	NA	NA
your vitamins andrew lessman bone & body factors calcium-magnesium-phosphorus women's wellness soy isoflavones	Standard	novasoy ultra-concentrated extract (bean)	3	NA	NA
your vitamins andrew lessman bone & body factors calcium-magnesium-phosphorus women's wellness soy isoflavones	Standard	broccoli sprout extract	3	NA	NA
your vitamins andrew lessman bone & body factors calcium-magnesium-phosphorus women's wellness soy isoflavones	Standard	indole-3-carbinol	4	NA	NA
optimum nutrition women's premium multiple opti-women more than a multi with ostivone, soy isoflavones, garcinia extract	Standard	biotin	1	NA	NA
optimum nutrition women's premium multiple opti-women more than a multi with ostivone, soy isoflavones, garcinia extract	Standard	butchers broom root	3	NA	NA

optimum nutrition women's premium multiple opti-women more than a multi with ostivone, soy isoflavones, garcinia extract	Standard	calcium	2	NA	NA
optimum nutrition women's premium multiple opti-women more than a multi with ostivone, soy isoflavones, garcinia extract	Standard	chromium	2	NA	NA
optimum nutrition women's premium multiple opti-women more than a multi with ostivone, soy isoflavones, garcinia extract	Standard	copper	2	NA	NA
optimum nutrition women's premium multiple opti-women more than a multi with ostivone, soy isoflavones, garcinia extract	Standard	iodine	2	NA	NA
optimum nutrition women's premium multiple opti-women more than a multi with ostivone, soy isoflavones, garcinia extract	Standard	iron	2	NA	NA
optimum nutrition women's premium multiple opti-women more than a multi with ostivone, soy isoflavones, garcinia extract	Standard	lutein	4	NA	NA
optimum nutrition women's premium multiple opti-women more than a multi with ostivone, soy isoflavones, garcinia extract	Standard	magnesium	2	NA	NA
optimum nutrition women's premium multiple opti-women more than a multi with ostivone, soy isoflavones, garcinia extract	Standard	manganese	2	NA	NA
optimum nutrition women's premium multiple opti-women more than a multi with ostivone, soy isoflavones, garcinia extract	Standard	molybdenum	2	NA	NA
optimum nutrition women's premium multiple opti-women more than a multi with ostivone, soy isoflavones, garcinia extract	Standard	pantothenic acid	1	NA	NA
optimum nutrition women's premium multiple opti-women	Standard	selenium	2	NA	NA

more than a multi with ostivone, soy isoflavones, garcinia extract					
optimum nutrition women's premium multiple opti-women more than a multi with ostivone, soy isoflavones, garcinia extract	Standard	vitamin a	1	NA	NA
optimum nutrition women's premium multiple opti-women more than a multi with ostivone, soy isoflavones, garcinia extract	Standard	vitamin d	1	NA	NA
optimum nutrition women's premium multiple opti-women more than a multi with ostivone, soy isoflavones, garcinia extract	Standard	vitamin e	1	NA	NA
optimum nutrition women's premium multiple opti-women more than a multi with ostivone, soy isoflavones, garcinia extract	Standard	vitamin k	1	NA	NA
optimum nutrition women's premium multiple opti-women more than a multi with ostivone, soy isoflavones, garcinia extract	Standard	zinc	2	NA	NA
optimum nutrition women's premium multiple opti-women more than a multi with ostivone, soy isoflavones, garcinia extract	Standard	soy isoflavones	3	NA	NA
optimum nutrition women's premium multiple opti-women more than a multi with ostivone, soy isoflavones, garcinia extract	Standard	alpha lipoic acid	4	NA	NA
optimum nutrition women's premium multiple opti-women more than a multi with ostivone, soy isoflavones, garcinia extract	Standard	lycopene	4	NA	NA
optimum nutrition women's premium multiple opti-women more than a multi with ostivone, soy isoflavones, garcinia extract	Standard	zeaxanthin	4	NA	NA
optimum nutrition women's premium multiple opti-women more than a multi with ostivone, soy isoflavones, garcinia extract	Standard	cryptoxanthin	4	NA	NA

optimum nutrition women's premium multiple opti-women more than a multi with ostivone, soy isoflavones, garcinia extract	Standard	grape seed extract (seed)	3	NA	NA
optimum nutrition women's premium multiple opti-women more than a multi with ostivone, soy isoflavones, garcinia extract	Standard	uva ursi (leaf)	3	NA	NA
optimum nutrition women's premium multiple opti-women more than a multi with ostivone, soy isoflavones, garcinia extract	Standard	deodorized garlic (bulb)	3	NA	NA
optimum nutrition women's premium multiple opti-women more than a multi with ostivone, soy isoflavones, garcinia extract	Standard	vitamin c	1	NA	NA
optimum nutrition women's premium multiple opti-women more than a multi with ostivone, soy isoflavones, garcinia extract	Standard	citrus bioflavonoids (fruit)	3	NA	NA
optimum nutrition women's premium multiple opti-women more than a multi with ostivone, soy isoflavones, garcinia extract	Standard	horse chestnut extract	3	NA	NA
optimum nutrition women's premium multiple opti-women more than a multi with ostivone, soy isoflavones, garcinia extract	Standard	dong quai root extract 4:1	3	NA	NA
optimum nutrition women's premium multiple opti-women more than a multi with ostivone, soy isoflavones, garcinia extract	Standard	niacin	1	NA	NA
optimum nutrition women's premium multiple opti-women more than a multi with ostivone, soy isoflavones, garcinia extract	Standard	thiamin	1	NA	NA
optimum nutrition women's premium multiple opti-women more than a multi with ostivone, soy isoflavones, garcinia extract	Standard	riboflavin	1	NA	NA
optimum nutrition women's premium multiple opti-women	Standard	chasteberry extract	3	NA	NA

more than a multi with ostivone, soy isoflavones, garcinia extract					
optimum nutrition women's premium multiple opti-women more than a multi with ostivone, soy isoflavones, garcinia extract	Standard	alpha-carotene	4	NA	NA
optimum nutrition women's premium multiple opti-women more than a multi with ostivone, soy isoflavones, garcinia extract	Standard	vitamin b-6	1	NA	NA
optimum nutrition women's premium multiple opti-women more than a multi with ostivone, soy isoflavones, garcinia extract	Standard	vitamin b-12	1	NA	NA
optimum nutrition women's premium multiple opti-women more than a multi with ostivone, soy isoflavones, garcinia extract	Standard	folic acid	1	NA	NA
optimum nutrition women's premium multiple opti-women more than a multi with ostivone, soy isoflavones, garcinia extract	Standard	garcinia cambogia	3	NA	NA
optimum nutrition women's premium multiple opti-women more than a multi with ostivone, soy isoflavones, garcinia extract	Standard	ostivone (ipriflavone)	4	NA	NA
good 'n natural time release ultra woman high potency vitamin, mineral, herb formula for women	Standard	soy isoflavones	3	NA	NA
good 'n natural time release ultra woman high potency vitamin, mineral, herb formula for women	Standard	ipriflavone	4	NA	NA
promensil plant estrogens extracted from red clover	Standard	isoflavones	4	NA	NA
walgreens woman's way 50 plus scientifically formulated for active women over 50 multivitamin with soy isoflavones, calc	Mature	biotin	1	NA	NA
walgreens woman's way 50 plus scientifically formulated for active women over 50 multivitamin with soy isoflavones, calc	Mature	calcium	2	NA	NA
walgreens woman's way 50 plus scientifically	Mature	chromium	2	NA	NA

formulated for active women over 50 multivitamin with soy isoflavones, calc					
walgreens woman's way 50 plus scientifically formulated for active women over 50 multivitamin with soy isoflavones, calc	Mature	copper	2	NA	NA
walgreens woman's way 50 plus scientifically formulated for active women over 50 multivitamin with soy isoflavones, calc	Mature	magnesium	2	NA	NA
walgreens woman's way 50 plus scientifically formulated for active women over 50 multivitamin with soy isoflavones, calc	Mature	manganese	2	NA	NA
walgreens woman's way 50 plus scientifically formulated for active women over 50 multivitamin with soy isoflavones, calc	Mature	pantothenic acid	1	NA	NA
walgreens woman's way 50 plus scientifically formulated for active women over 50 multivitamin with soy isoflavones, calc	Mature	potassium	4	NA	NA
walgreens woman's way 50 plus scientifically formulated for active women over 50 multivitamin with soy isoflavones, calc	Mature	selenium	2	NA	NA
walgreens woman's way 50 plus scientifically formulated for active women over 50 multivitamin with soy isoflavones, calc	Mature	vitamin a	1	NA	NA
walgreens woman's way 50 plus scientifically formulated for active women over 50 multivitamin with soy isoflavones, calc	Mature	vitamin d	1	NA	NA
walgreens woman's way 50 plus scientifically formulated for active women over 50 multivitamin with soy isoflavones, calc	Mature	vitamin e	1	NA	NA
walgreens woman's way 50 plus scientifically formulated for active women over 50 multivitamin with soy	Mature	vitamin k	1	NA	NA

isoflavones, calc					
walgreens woman's way 50 plus scientifically formulated for active women over 50 multivitamin with soy isoflavones, calc	Mature	zinc	2	NA	NA
walgreens woman's way 50 plus scientifically formulated for active women over 50 multivitamin with soy isoflavones, calc	Mature	vitamin c	1	NA	NA
walgreens woman's way 50 plus scientifically formulated for active women over 50 multivitamin with soy isoflavones, calc	Mature	soy extract	3	NA	NA
walgreens woman's way 50 plus scientifically formulated for active women over 50 multivitamin with soy isoflavones, calc	Mature	niacin	1	NA	NA
walgreens woman's way 50 plus scientifically formulated for active women over 50 multivitamin with soy isoflavones, calc	Mature	thiamin	1	NA	NA
walgreens woman's way 50 plus scientifically formulated for active women over 50 multivitamin with soy isoflavones, calc	Mature	riboflavin	1	NA	NA
walgreens woman's way 50 plus scientifically formulated for active women over 50 multivitamin with soy isoflavones, calc	Mature	vitamin b-6	1	NA	NA
walgreens woman's way 50 plus scientifically formulated for active women over 50 multivitamin with soy isoflavones, calc	Mature	vitamin b-12	1	NA	NA
walgreens woman's way 50 plus scientifically formulated for active women over 50 multivitamin with soy isoflavones, calc	Mature	folic acid	1	NA	NA
usana healthpak 100	Standard	soy isoflavones	3	NA	NA
nature's resource ginkgo biloba 120 mg extract time release formula	Standard	flavone glycosides	4	NA	NA
feminelle	Standard	soy isoflavones	3	NA	NA
puritan's pride time	Standar	soy isoflavones	3	NA	NA

release mega vita min for women high potency	d				
puritan's pride time release mega vita min for women high potency	Standard	ipriflavone	4	NA	NA
the vitamin shoppe osteo protector with ipriflavone	Standard	calcium	2	NA	NA
the vitamin shoppe osteo protector with ipriflavone	Standard	magnesium	2	NA	NA
the vitamin shoppe osteo protector with ipriflavone	Standard	vitamin d	1	NA	NA
the vitamin shoppe osteo protector with ipriflavone	Standard	ipriflavone	4	NA	NA
gnc women's phyto-estrogen formula	Standard	soy isoflavone extract	3	NA	NA
nature's way soy isoflavones with digestive enzymes non-gmo soylife	Standard	calories	4	NA	NA
nature's way soy isoflavones with digestive enzymes non-gmo soylife	Standard	protein	4	NA	NA
nature's way soy isoflavones with digestive enzymes non-gmo soylife	Standard	soylife 30 non-gmo	4	NA	NA
nature made standardized extract ginkgo biloba 30 mg extract	Standard	flavone glycosides	4	NA	NA
now extra strength soy isoflavones non-genetically engineered 60 mg of isoflavones	Standard	soy isoflavone extract	3	NA	NA
procaps laboratories bone & body factors calcium-magnesium-phosphorus intensive care women's wellness soy	Standard	isoflavones	4	NA	NA
rite aid whole source multivitamin complete formula for women	Standard	soy isoflavone extract	3	NA	NA
pharmanex lifepak prime anti-aging packets	Standard	isoflavones	4	NA	NA
estroven maximum strength 1,000 iu vitamin d3	Standard	isoflavones	4	NA	NA
gnc women's women's ultra mega menopause vitapak	Mature	soy isoflavone concentrate	3	NA	NA
estroven regular strength	Mature	isoflavones	4	NA	NA
gnc women's menopause formula	Mature	soy isoflavone concentrate	3	NA	NA
usana optimizers palmetto plus	Standard	soy isoflavones (seed)	4	NA	NA
equate regular strength estroblend before, during and after menopause soy, and other plants and vitamins d, b6, e & calc	Mature	isoflavones	4	NA	NA
metagenics iso d3 2,000 iu of vitamin d3 with isoflavones	Standard	vitamin d	1	NA	NA
metagenics iso d3 2,000	Standar	soy (bean	3	NA	NA

iu of vitamin d3 with isoflavones	d	concentrate)			
metagenics iso d3 2,000 iu of vitamin d3 with isoflavones	Standard	total isoflavones	4	NA	NA
puritan's pride time release mega vita min for women iron free	Standard	soy isoflavones	3	NA	NA
puritan's pride time release mega vita min for women iron free	Standard	ipriflavone	4	NA	NA
nutrilite bone health with ipriflavone	Standard	vitamin d	1	NA	NA
nutrilite bone health with ipriflavone	Standard	ipriflavone	4	NA	NA
nutrilite bone health with ipriflavone	Standard	chicory root concentrate	3	NA	NA
prostanol doctor's premium vitalmax	Standard	methoxyisoflavone	4	NA	NA
pure essence labs longevity anti-aging multiple women's formula	Standard	ipriflavone	4	NA	NA
radiance standardized extract ginkgo biloba 120 mg standardized to contain 24% ginkgo flavone glycosides	Standard	ginkgo biloba leaf extract	3	NA	NA
default soy isoflavones	Standard	calcium	2	NA	NA
default soy isoflavones	Standard	soy isoflavones	3	NA	NA
one a day menopause formula complete women's multivitamin multivitamin / multimineral complete multivitamin & soy isoflavones	Mature	beta carotene-% of vitamin a	4	NA	NA
one a day menopause formula complete women's multivitamin multivitamin / multimineral complete multivitamin & soy isoflavones	Mature	biotin	1	NA	NA
one a day menopause formula complete women's multivitamin multivitamin / multimineral complete multivitamin & soy isoflavones	Mature	boron	2	NA	NA
one a day menopause formula complete women's multivitamin multivitamin / multimineral complete multivitamin & soy isoflavones	Mature	calcium	2	NA	NA
one a day menopause formula complete women's multivitamin multivitamin /	Mature	chromium	2	NA	NA

multimineral complete multivitamin & soy isoflavones					
one a day menopause formula complete women's multivitamin multivitamin / multimineral complete multivitamin & soy isoflavones	Mature	copper	2	NA	NA
one a day menopause formula complete women's multivitamin multivitamin / multimineral complete multivitamin & soy isoflavones	Mature	iodine	2	NA	NA
one a day menopause formula complete women's multivitamin multivitamin / multimineral complete multivitamin & soy isoflavones	Mature	magnesium	2	NA	NA
one a day menopause formula complete women's multivitamin multivitamin / multimineral complete multivitamin & soy isoflavones	Mature	manganese	2	NA	NA
one a day menopause formula complete women's multivitamin multivitamin / multimineral complete multivitamin & soy isoflavones	Mature	molybdenum	2	NA	NA
one a day menopause formula complete women's multivitamin multivitamin / multimineral complete multivitamin & soy isoflavones	Mature	pantothenic acid	1	NA	NA
one a day menopause formula complete women's multivitamin multivitamin / multimineral complete multivitamin & soy isoflavones	Mature	selenium	2	NA	NA
one a day menopause formula complete women's multivitamin multivitamin / multimineral complete multivitamin & soy isoflavones	Mature	vitamin a	1	NA	NA
one a day menopause formula complete women's multivitamin multivitamin /	Mature	vitamin d	1	NA	NA

multimineral complete multivitamin & soy isoflavones					
one a day menopause formula complete women's multivitamin multivitamin / multimineral complete multivitamin & soy isoflavones	Mature	vitamin e	1	NA	NA
one a day menopause formula complete women's multivitamin multivitamin / multimineral complete multivitamin & soy isoflavones	Mature	zinc	2	NA	NA
one a day menopause formula complete women's multivitamin multivitamin / multimineral complete multivitamin & soy isoflavones	Mature	total carbohydrate	4	NA	NA
one a day menopause formula complete women's multivitamin multivitamin / multimineral complete multivitamin & soy isoflavones	Mature	vitamin c	1	NA	NA
one a day menopause formula complete women's multivitamin multivitamin / multimineral complete multivitamin & soy isoflavones	Mature	niacin	1	NA	NA
one a day menopause formula complete women's multivitamin multivitamin / multimineral complete multivitamin & soy isoflavones	Mature	thiamin	1	NA	NA
one a day menopause formula complete women's multivitamin multivitamin / multimineral complete multivitamin & soy isoflavones	Mature	riboflavin	1	NA	NA
one a day menopause formula complete women's multivitamin multivitamin / multimineral complete multivitamin & soy isoflavones	Mature	vitamin b-6	1	NA	NA
one a day menopause formula complete women's multivitamin multivitamin /	Mature	vitamin b-12	1	NA	NA

multimineral complete multivitamin & soy isoflavones					
one a day menopause formula complete women's multivitamin multivitamin / multimineral complete multivitamin & soy isoflavones	Mature	folic acid	1	NA	NA
one a day menopause formula complete women's multivitamin multivitamin / multimineral complete multivitamin & soy isoflavones	Mature	soybean isoflavones extract	4	NA	NA
solgar male multiple advanced phytonutrient multiple vitamin, mineral and herbal formula for men	Standard	soy isoflavone seed extract	4	NA	NA
projoba international projoba super daily food based vitamin / mineral complex	Standard	soy isoflavones	3	NA	NA
default chrysin (5,7 - dihydroxyflavone)	Standard	chrysin	4	NA	NA
spring valley soy isoflavones 40 mg	Standard	calcium	2	NA	NA
spring valley soy isoflavones 40 mg	Standard	soy isoflavones	3	NA	NA
barlean's organic oils the essential woman 1000 mg	Standard	isoflavones	4	NA	NA
equate regular strength estroblend natural 1 per day	Mature	isoflavones	4	NA	NA
solgar standardized full potency herbal female complex	Standard	soy isoflavone seed extract	4	NA	NA
nature's bounty double strength standardized extract ginkgo biloba 120 mg standardized to contain 24% ginkgo flavone glycosides	Standard	ginkgo biloba leaf extract	3	NA	NA
gnc phyto-estrogen formula non-gmo soy isoflavone extract isolase enzyme system black cohosh extract evening primrose oil b-vitamins & minerals	Standard	calcium	2	NA	NA
gnc phyto-estrogen formula non-gmo soy isoflavone extract isolase enzyme system black cohosh extract evening primrose oil b-vitamins & minerals	Standard	calories	4	NA	NA
gnc phyto-estrogen formula non-gmo soy isoflavone extract isolase	Standard	evening primrose oil	3	NA	NA

enzyme system black cohosh extract evening primrose oil b-vitamins & minerals					
gnc phyto-estrogen formula non-gmo soy isoflavone extract isolase enzyme system black cohosh extract evening primrose oil b-vitamins & minerals	Standard	magnesium	2	NA	NA
gnc phyto-estrogen formula non-gmo soy isoflavone extract isolase enzyme system black cohosh extract evening primrose oil b-vitamins & minerals	Standard	total fat	4	NA	NA
gnc phyto-estrogen formula non-gmo soy isoflavone extract isolase enzyme system black cohosh extract evening primrose oil b-vitamins & minerals	Standard	calories from fat	4	NA	NA
gnc phyto-estrogen formula non-gmo soy isoflavone extract isolase enzyme system black cohosh extract evening primrose oil b-vitamins & minerals	Standard	soy isoflavone extract	3	NA	NA
gnc phyto-estrogen formula non-gmo soy isoflavone extract isolase enzyme system black cohosh extract evening primrose oil b-vitamins & minerals	Standard	black cohosh root extract	3	NA	NA
gnc phyto-estrogen formula non-gmo soy isoflavone extract isolase enzyme system black cohosh extract evening primrose oil b-vitamins & minerals	Standard	vitamin b-6	1	NA	NA
gnc phyto-estrogen formula non-gmo soy isoflavone extract isolase enzyme system black cohosh extract evening primrose oil b-vitamins & minerals	Standard	vitamin b-12	1	NA	NA
gnc phyto-estrogen formula non-gmo soy isoflavone extract isolase enzyme system black cohosh extract evening primrose oil b-vitamins & minerals	Standard	isolase	4	NA	NA
estroven maximum strength one per day!	Standard	isoflavones	4	NA	NA
gnc natural brand soy	Standar	cranberry	3	NA	NA

isoflavone concentrate with cranberry	d	concentrate			
gnc natural brand soy isoflavone concentrate with cranberry	Standard	soy isoflavone concentrate	3	NA	NA
nutrilite black cohosh and soy	Standard	isoflavones	4	NA	NA
gnc menopause formula standardized soy isoflavones 160 mg of black cohosh root extract	Mature	black cohosh root extract	3	NA	NA
gnc menopause formula standardized soy isoflavones 160 mg of black cohosh root extract	Mature	soy isoflavone concentrate	3	NA	NA
gnc menopause formula standardized soy isoflavones 160 mg of black cohosh root extract	Mature	isolase	4	NA	NA
vitamed isovit glycine max 75 mg	Standard	isoflavones	4	NA	NA
puritan's pride premium ginkgo biloba 60 mg standardized extract standardized to contain 24% ginkgo flavone glycosides	Standard	ginkgo biloba extract (leaf)	3	NA	NA
opti-women high-potency 40 ingredients on	Standard	soy isoflavones	3	NA	NA
opti-women high-potency 40 ingredients on	Standard	ostivone (ipriflavone)	4	NA	NA
women's wellness procaps laboratories	Standard	isoflavones	4	NA	NA
womensense with ipriflavone osteosense plus	Standard	betaine hydrochloride	4	NA	NA
womensense with ipriflavone osteosense plus	Standard	boron	2	NA	NA
womensense with ipriflavone osteosense plus	Standard	calcium	2	NA	NA
womensense with ipriflavone osteosense plus	Standard	citrus bioflavonoids	3	NA	NA
womensense with ipriflavone osteosense plus	Standard	copper	2	NA	NA
womensense with ipriflavone osteosense plus	Standard	magnesium	2	NA	NA
womensense with ipriflavone osteosense plus	Standard	phosphorus	2	NA	NA
womensense with ipriflavone osteosense plus	Standard	silica	2	NA	NA
womensense with ipriflavone osteosense plus	Standard	vitamin d	1	NA	NA
womensense with	Standar	vitamin k	1	NA	NA

ipriflavone osteosense plus	d				
womensense with ipriflavone osteosense plus	Standard	zinc	2	NA	NA
womensense with ipriflavone osteosense plus	Standard	total carbohydrate	4	NA	NA
womensense with ipriflavone osteosense plus	Standard	vitamin c	1	NA	NA
womensense with ipriflavone osteosense plus	Standard	ipriflavone	4	NA	NA
womensense with ipriflavone osteosense plus	Standard	vitamin b-6	1	NA	NA
womensense with ipriflavone osteosense plus	Standard	vitamin b-12	1	NA	NA
womensense with ipriflavone osteosense plus	Standard	folic acid	1	NA	NA
womensense with ipriflavone osteosense plus	Standard	bamboo extract (leaf & stem)	3	NA	NA
kowa okinawa life	Standard	isoflavones	4	NA	NA
nature's bounty standardized extract ginkgo biloba 60 mg per serving standardized to contain 24% ginkgo flavone glycosides	Standard	ginkgo biloba leaf extract	3	NA	NA
spring valley soy isoflavones 40 mg per tablet	Standard	calcium	2	NA	NA
spring valley soy isoflavones 40 mg per tablet	Standard	soy isoflavones	3	NA	NA
botanic choice bilberry plus mirtoselect	Standard	flavonols, flavones and related phenolic compounds	4	NA	NA
options healthy woman soy zero calories	Standard	soy isoflavones	3	NA	NA
estroven maximum strength + energy drug free & estrogen free	Standard	soy isoflavones	3	NA	NA
gnc women's ultra mega menopause vitapak program clinically studied multivitamin with 2,000 iu vitamin d-3 enhanced formula with omega-3s with 1,000 mg calcium	Mature	soy isoflavone concentrate	3	NA	NA
equate maximum strength estroblend multi-symptom menopause formula 1 per day	Standard	soy isoflavone concentrate	3	NA	NA
vita logic osteo protect plus! with vitamin k2 &	Standard	boron	2	NA	NA

ipriflavone triple source calcium					
vita logic osteo protect plus! with vitamin k2 & ipriflavone triple source calcium	Standard	bromelain	4	NA	NA
vita logic osteo protect plus! with vitamin k2 & ipriflavone triple source calcium	Standard	calcium	2	NA	NA
vita logic osteo protect plus! with vitamin k2 & ipriflavone triple source calcium	Standard	magnesium	2	NA	NA
vita logic osteo protect plus! with vitamin k2 & ipriflavone triple source calcium	Standard	manganese	2	NA	NA
vita logic osteo protect plus! with vitamin k2 & ipriflavone triple source calcium	Standard	phosphorus	2	NA	NA
vita logic osteo protect plus! with vitamin k2 & ipriflavone triple source calcium	Standard	silica	2	NA	NA
vita logic osteo protect plus! with vitamin k2 & ipriflavone triple source calcium	Standard	vitamin d	1	NA	NA
vita logic osteo protect plus! with vitamin k2 & ipriflavone triple source calcium	Standard	vitamin e	1	NA	NA
vita logic osteo protect plus! with vitamin k2 & ipriflavone triple source calcium	Standard	vitamin k	1	NA	NA
vita logic osteo protect plus! with vitamin k2 & ipriflavone triple source calcium	Standard	vitamin c	1	NA	NA
vita logic osteo protect plus! with vitamin k2 & ipriflavone triple source calcium	Standard	ipriflavone	4	NA	NA
vita logic osteo protect plus! with vitamin k2 & ipriflavone triple source calcium	Standard	vitamin b-6	1	NA	NA
puritan's pride premium ginkgo biloba standardized to contain 24 % ginkgo flavone glycosides 60 mg standardized extract	Standard	ginkgo biloba extract (leaf)	3	NA	NA
on opti-women high-potency 40+ ingredients	Standard	soy isoflavones	3	NA	NA
on opti-women high-potency 40+ ingredients	Standard	ostivone (ipriflavone)	4	NA	NA
Search for “flavonol”					
nature's way alive! whole food energizer with iron	Standard	alive! citrus bioflavonoid	NA	flavonols	Other

veggie cap		complex			
nature's way alive! whole food energizer (iron free) veggie cap	Standard	alive! citrus bioflavonoid complex	NA	flavonols	Other
your vitamins andrew lessman circulation & vein support bioflavonoid complex diosmin hesperidin quercetin rutin	Standard	your vitamins flavonol complex	NA	flavonols	Other
your vitamins andrew lessman circulation & vein support bioflavonoid complex diosmin hesperidin quercetin rutin	Standard	your vitamins flavonol complex	NA	flavones	Other
your vitamins andrew lessman circulation & vein support bioflavonoid complex diosmin hesperidin quercetin rutin	Standard	your vitamins flavonol complex	NA	phenols	Other
nature's plus source of life vitamin, mineral & protein energy shake with whole food concentrates	Standard	nature's plus source of life energy shake lemon bioflavonoid complex (citrus)	NA	flavonols	Other
nature's way alive! whole food energizer men's multi max potency vitamins & minerals 26 fruits & vegetables green foods enzymes mushrooms amino acids antioxidants lutein resveratrol	Standard	nature's way alive! citrus bioflavonoid complex #3	NA	flavonols	Other
nature's way alive! once daily men's ultra potency	Standard	nature's way alive! citrus bioflavonoid complex #3	NA	flavonols	Other
nature's way alive! once daily women's ultra potency	Standard	nature's way alive! citrus bioflavonoid complex #3	NA	flavonols	Other
nature's way alive! whole food energizer multi-vitamin max potency vitamins & minerals 26 fruits & vegetables green foods enzymes mushrooms amino acids antioxidants lutein resveratrol	Standard	nature's way alive! citrus bioflavonoid complex #3	NA	flavonols	Other
nature's way alive! once daily multi-vitamin ultra potency whole food energizer 26 fruits & vegetables green foods mushrooms enzymes antioxidants lutein resveratrol	Standard	nature's way alive! citrus bioflavonoid complex #3	NA	flavonols	Other
nature's way alive! whole food energizer women's multi max potency	Standard	nature's way alive! citrus bioflavonoid complex #3	NA	flavonols	Other
nature's way alive! once daily men's ultra potency multi-vitamin & whole	Standard	nature's way alive! citrus bioflavonoid complex #3	NA	flavonols	Other

food energizer					
nature's way alive! whole food energizer men's multi max potency	Standard	nature's way alive! citrus bioflavonoid complex #3	NA	flavonols	Other
nature's way alive! once daily women's ultra potency multi-vitamin & whole food energizer	Standard	nature's way alive! citrus bioflavonoid complex #3	NA	flavonols	Other
nature's way alive! once daily multi-vitamin ultra potency energizer with food based blends orchard fruits / garden veggies & daily greens powder (120 mg), enzymes, bioflavonoids multi-vitamin	Standard	nature's way alive! citrus bioflavonoid complex #3	NA	flavonols	Other
twinlab citrus bioflavonoid caps with rutin	Standard	twinlab citrus bioflavonoid caps citrus bioflavonoids complex	NA	flavonols	Other
bluebonnet c-1000 plus bioflavonoids vitamin c plus citrus bioflavonoids	Standard	flavonols	4	NA	NA
default citrus bioflavonoids	Standard	flavonols	4	NA	NA
botanic choice bilberry plus mirtoselect	Standard	flavonols, flavones and related phenolic compounds	4	NA	NA
Search for “isoflavone”					
newphase phytoestrogen support for women sunsource	Mature	newphase multi-herbal isoflavone blend	NA	soy protein concentrate	Botanical
newphase complete all-natural drug free	Mature	newphase multi-herbal isoflavone blend	NA	soy protein concentrate	Botanical
newphase phytoestrogen support for women sunsource	Mature	newphase multi-herbal isoflavone blend	NA	red clover extract	Botanical
newphase complete all-natural drug free	Mature	newphase multi-herbal isoflavone blend	NA	red clover extract	Botanical
newphase phytoestrogen support for women sunsource	Mature	newphase multi-herbal isoflavone blend	NA	kudzu extract (root)	Botanical
newphase complete all-natural drug free	Mature	newphase multi-herbal isoflavone blend	NA	kudzu extract (root)	Botanical
ladies choice	Standard	ladies choice proprietary blend	NA	soy isoflavones	Botanical
nikken for women with isoflavone plus complex	Standard	nikken isoflavone plus complex	NA	n-acetyl l-cysteine	Amino acid
nikken for women with isoflavone plus complex	Standard	nikken isoflavone plus complex	NA	soy isoflavones	Botanical
nikken for women with isoflavone plus complex	Standard	nikken isoflavone plus complex	NA	dong quai (root)	Botanical
nikken for women with isoflavone plus complex	Standard	nikken isoflavone plus complex	NA	black cohosh (root)	Botanical
nikken for women with isoflavone plus complex	Standard	nikken isoflavone plus complex	NA	fenugreek (whole plant)	Botanical
nuhair hair regrowth	Standar	nuhair follicle	NA	isoflavones	Other

tablets for men 100% natural	d	stimulating botanical blend			
super juice daily multi phyto-nutrient formula vegetable, fruit & botanical now with botanical antioxidants	Standard	super juice isoflavone concentrate blend	NA	soybean	Botanical
super juice daily multi phyto-nutrient formula vegetable, fruit & botanical now with botanical antioxidants! supplies whole food nutrition from six daily servings!	Standard	super juice isoflavone concentrate blend	NA	soybean	Botanical
super juice daily multi phyto-nutrient formula vegetable, fruit & botanical now with botanical antioxidants	Standard	super juice isoflavone concentrate blend	NA	kudzu root	Botanical
super juice daily multi phyto-nutrient formula vegetable, fruit & botanical now with botanical antioxidants! supplies whole food nutrition from six daily servings!	Standard	super juice isoflavone concentrate blend	NA	kudzu root	Botanical
vitality mineral complex with calcium patented fructose compounding	Standard	vitality mineral complex proprietary blend	NA	soy isoflavones	Botanical
ritestart women all-in-one nutrition for women with 4life transfer factor plus exclusive anti-aging formula 4life packet	Standard	4life women's health blend	NA	soy isoflavones	Botanical
ritestart men all-in -one nutrition for men with 4life transfer factor plus advanced formula exclusive anti-aging formul	Standard	4life men's health blend	NA	soy isoflavones	Botanical
newphase complete all-natural drug free new improved formula	Mature	newphase multi-herbal isoflavone blend #2	NA	soybean	Botanical
newphase complete all-natural drug free new improved formula	Mature	newphase multi-herbal isoflavone blend #2	NA	kudzu extract (root)	Botanical
newphase complete all-natural drug free new improved formula	Mature	newphase multi-herbal isoflavone blend #2	NA	red clover extract (leaf)	Botanical
equate estroplus extra strength	Standard	equate estroplus isoflavones blend	NA	soy extract (bean)	Botanical
equate estroplus extra strength	Standard	equate estroplus isoflavones blend	NA	kudzu extract (root)	Botanical
equate estroplus extra strength	Standard	equate estroplus isoflavones blend	NA	plant enzymes	Other
sentia	Standard	sentia proprietary blend	NA	isoflavones	Other
one source ultimate women's premium multivitamin designed for women vitamin d 1000 iu	Standard	one source ultimate women's antioxidant fruit and vegetable	NA	soy isoflavone extract	Botanical

calcium 500 mg per serving		blend			
one source ultimate women's multi vitamin d3 1000 iu per serving	Standard	one source ultimate women's antioxidant fruit and vegetable blend	NA	soy isoflavone extract	Botanical
vitality gold for men am / pm high potency	Standard	vitality gold for men am / pm proprietary blend	NA	soy isoflavones	Botanical
natural balance ladies choice phytoestrogen formula	Standard	natural balance ladies choice proprietary blend	NA	soy isoflavones	Botanical
cvs pharmacy menopause support extra strength	Standard	cvs pharmacy menopause support isoflavones blend	NA	soy extract (bean)	Botanical
cvs pharmacy menopause support extra strength	Standard	cvs pharmacy menopause support isoflavones blend	NA	kudzu extract (root)	Botanical
daily for life for women am/pm high potency packets	Standard	daily for life for women proprietary blend #1	NA	soy isoflavones	Botanical
member's mark estro vital nutrients extra strength 2 caplets daily	Mature	member's mark estro vital nutrients isoflavones blend	NA	soy extract (bean)	Botanical
member's mark estro vital nutrients extra strength 2 caplets daily	Mature	member's mark estro vital nutrients isoflavones blend	NA	kudzu extract (root)	Botanical
member's mark estro vital nutrients extra strength 2 caplets daily	Mature	member's mark estro vital nutrients isoflavones blend	NA	plant enzymes	Other
mannatech optimal health system glycentials antioxidant formula complete vitamin & mineral vitamin, ambroglycin mineral	Standard	phytonutrient complex 2 (pnc)	NA	isoflavones	Other
ultra woman daily multi premium performance formula for women high potency timed release	Standard	ultra woman bone blend	NA	soy isoflavones	Botanical
onesource multivitamin women's with vitamin d3 4000 iu per serving	Standard	onesource multivitamin women's proprietary antioxidant fruit and vegetable blend	NA	soy isoflavone extract	Botanical
rite aid pharmacy extra strength menopause support soy, cranberry and calcium complex formula	Standard	rite aid pharmacy extra strength menopause support isoflavone blend	NA	soy isoflavones	Botanical
rite aid pharmacy extra strength menopause support soy, cranberry and calcium complex formula	Standard	rite aid pharmacy extra strength menopause support isoflavone blend	NA	kudzu root	Botanical
soy care bone health	Standard	soy isoflavones	3	NA	NA
lifepak	Standar	isoflavones	4	NA	NA

	d				
lifepak prime	Standard	isoflavones	4	NA	NA
lifepak women	Standard	isoflavones	4	NA	NA
lifepak trim	Standard	isoflavones	4	NA	NA
gnc women's menopause vita pak	Mature	soy isoflavones	3	NA	NA
caltrate 600 + soy with soy isoflavones	Standard	calcium	2	NA	NA
caltrate 600 + soy with soy isoflavones	Standard	vitamin d	1	NA	NA
caltrate 600 + soy with soy isoflavones	Standard	soy isoflavones	3	NA	NA
natrol for women soy isoflavones with genistein & daidzein	Standard	isoflavones	4	NA	NA
natrol for women soy isoflavones with genistein & daidzein	Standard	soy isoflavone extract	3	NA	NA
natrol for women soy isoflavones with genistein & daidzein	Standard	daidzin & daidzein	4	NA	NA
natrol for women soy isoflavones with genistein & daidzein	Standard	genistin & genistein	4	NA	NA
natrol for women soy isoflavones with genistein & daidzein	Standard	glycitin & glycitein	4	NA	NA
natrol for women menopause formula	Mature	soy isoflavones	3	NA	NA
gnc herbal rush	Standard	soy isoflavone concentrate	3	NA	NA
your life calcium 500 mg with vitamin d 200 iu plus soy 15mg contains novasoy	Standard	isoflavones	4	NA	NA
women's formula advanced breast health formula with patented calcium d-glucarate	Standard	isoflavones	4	NA	NA
free life osteosoy	Standard	isoflavones	4	NA	NA
gnc women's menopause vita pak	Mature	soy isoflavones	3	NA	NA
gnc natural brand soy isoflavone concentrate 50 mg	Standard	soy isoflavone concentrate	3	NA	NA
solar green phytoestrogen greens	Standard	isoflavones	4	NA	NA
nature's plus ultra isoflavone 100	Standard	isoflavones	4	NA	NA
nature's plus ultra isoflavone 100	Standard	genistein	4	NA	NA
nature's plus ultra isoflavone 100	Standard	puerarin	4	NA	NA
nature's plus ultra isoflavone 100	Standard	daidzin	4	NA	NA
nature's plus ultra isoflavone 100	Standard	daidzein	4	NA	NA
nature's plus ultra	Standar	glycitin	4	NA	NA

isoflavone 100	d				
nature's plus ultra isoflavone 100	Standard	genistin	4	NA	NA
nature's plus ultra isoflavone 100	Standard	glycitein	4	NA	NA
vitamin world time release mega vita min for women high potency	Standard	soy isoflavones	3	NA	NA
health from the sun fermented soy essentials whole food 800 mg	Standard	isoflavones	4	NA	NA
estroven soy and black cohosh plus vitamins b-6, e & calcium	Mature	isoflavones	4	NA	NA
nature's way standardized soy isoflavone extract	Standard	soy isoflavone extract	3	NA	NA
nature's way standardized soy isoflavone extract	Standard	red clover flower	3	NA	NA
sundown soy isoflavones	Standard	calcium	2	NA	NA
sundown soy isoflavones	Standard	soy isoflavone concentrate	3	NA	NA
vitadvance bone health with soy isoflavones	Standard	boron	2	NA	NA
vitadvance bone health with soy isoflavones	Standard	calcium	2	NA	NA
vitadvance bone health with soy isoflavones	Standard	copper	2	NA	NA
vitadvance bone health with soy isoflavones	Standard	magnesium	2	NA	NA
vitadvance bone health with soy isoflavones	Standard	manganese	2	NA	NA
vitadvance bone health with soy isoflavones	Standard	vitamin d	1	NA	NA
vitadvance bone health with soy isoflavones	Standard	vitamin k	1	NA	NA
vitadvance bone health with soy isoflavones	Standard	zinc	2	NA	NA
vitadvance bone health with soy isoflavones	Standard	soy isoflavones	3	NA	NA
vitadvance bone health with soy isoflavones	Standard	vitamin c	1	NA	NA
vitadvance bone health with soy isoflavones	Standard	vitamin b-6	1	NA	NA
vitadvance menopause support with black cohosh & soy	Mature	soy isoflavones	3	NA	NA
generic calcium 600 + soy with soy isoflavones	Standard	calcium	2	NA	NA
generic calcium 600 + soy with soy isoflavones	Standard	vitamin d	1	NA	NA
generic calcium 600 + soy with soy isoflavones	Standard	soy isoflavones	3	NA	NA
mini's vitamin mineral & herbal formula essentials by megafood full color spectrum foodbased nutrition 72% whole food	Standard	soy 1% isoflavones / genistein	3	NA	NA
anabolic laboratories bone support formula premium calcium complex with soy	Standard	boron	2	NA	NA

isoflavones & support nutrients					
anabolic laboratories bone support formula premium calcium complex with soy isoflavones & support nutrients	Standard	calcium	2	NA	NA
anabolic laboratories bone support formula premium calcium complex with soy isoflavones & support nutrients	Standard	copper	2	NA	NA
anabolic laboratories bone support formula premium calcium complex with soy isoflavones & support nutrients	Standard	magnesium	2	NA	NA
anabolic laboratories bone support formula premium calcium complex with soy isoflavones & support nutrients	Standard	manganese	2	NA	NA
anabolic laboratories bone support formula premium calcium complex with soy isoflavones & support nutrients	Standard	silicon	2	NA	NA
anabolic laboratories bone support formula premium calcium complex with soy isoflavones & support nutrients	Standard	vitamin d	1	NA	NA
anabolic laboratories bone support formula premium calcium complex with soy isoflavones & support nutrients	Standard	vitamin k	1	NA	NA
anabolic laboratories bone support formula premium calcium complex with soy isoflavones & support nutrients	Standard	zinc	2	NA	NA
anabolic laboratories bone support formula premium calcium complex with soy isoflavones & support nutrients	Standard	vitamin c	1	NA	NA
anabolic laboratories bone support formula premium calcium complex with soy isoflavones & support nutrients	Standard	novasoy isoflavone complex	4	NA	NA
anabolic laboratories	Standar	bioflavonoid	4	NA	NA

bone support formula premium calcium complex with soy isoflavones & support nutrients	d	complex			
nature's bounty non-gmo soy isoflavones with daidzein, genistein and other soy isoflavones	Standard	soy isoflavones	3	NA	NA
nature's bounty non-gmo soy isoflavones with daidzein, genistein and other soy isoflavones	Standard	daidzin & daidzein	4	NA	NA
nature's bounty non-gmo soy isoflavones with daidzein, genistein and other soy isoflavones	Standard	genistin & genistein	4	NA	NA
nature's bounty non-gmo soy isoflavones with daidzein, genistein and other soy isoflavones	Standard	glycitin & glycitein	4	NA	NA
nature's bounty non-gmo soy isoflavones with daidzein, genistein and other soy isoflavones	Standard	soy extract (soy life)	3	NA	NA
nature's bounty non-gmo soy isoflavones with daidzein, genistein and other soy isoflavones	Standard	soy saponins	3	NA	NA
prescriptive formulas women's optimal vitamin packs complete daily nutritional program	Standard	soy isoflavones	3	NA	NA
nikken for women with isoflavone plus complex	Standard	calcium	2	NA	NA
nikken for women with isoflavone plus complex	Standard	grape seed extract	3	NA	NA
nikken for women with isoflavone plus complex	Standard	iron	2	NA	NA
nikken for women with isoflavone plus complex	Standard	lutein	4	NA	NA
nikken for women with isoflavone plus complex	Standard	magnesium	2	NA	NA
nikken for women with isoflavone plus complex	Standard	vitamin d	1	NA	NA
nikken for women with isoflavone plus complex	Standard	vitamin e	1	NA	NA
nikken for women with isoflavone plus complex	Standard	melatonin	4	NA	NA
nikken for women with isoflavone plus complex	Standard	vitamin c	1	NA	NA
nikken for women with isoflavone plus complex	Standard	vitamin b-6	1	NA	NA
nikken for women with isoflavone plus complex	Standard	vitamin b-12	1	NA	NA
nikken for women with isoflavone plus complex	Standard	folic acid	1	NA	NA
nikken for women with isoflavone plus complex	Standard	cranberry fruit powder	3	NA	NA
default soy isoflavones	Standard	calcium	2	NA	NA
default soy isoflavones	Standard	soy isoflavone concentrate	3	NA	NA

healthy woman soy supplement 55 mg of soy isoflavones	Standard	calories	4	NA	NA
healthy woman soy supplement 55 mg of soy isoflavones	Standard	soy standardized extract	3	NA	NA
healthy woman soy supplement 55 mg of soy isoflavones	Standard	isoflavones (genistin/daidzin)	3	NA	NA
tabak's health products the mega wellness system	Standard	soy isoflavone extract	3	NA	NA
pharmanex lifepak new anti-aging formula	Standard	isoflavones	4	NA	NA
pharmanex lifepak new anti-aging formula	Standard	isoflavones	4	NA	NA
pharmacist's ultimate health super soy 10-12% isoflavones	Standard	soy standardized extract	3	NA	NA
pharmacist's ultimate health super soy 10-12% isoflavones	Standard	daidzin & daidzein	4	NA	NA
pharmacist's ultimate health super soy 10-12% isoflavones	Standard	genistin & genistein	4	NA	NA
pharmacist's ultimate health super soy 10-12% isoflavones	Standard	glycitin & glycitein	4	NA	NA
your life complete spectra multivitamin plus herbs with lutein, bilberry, vitamins a and c vitamins, minerals, and herbs	Standard	isoflavones	4	NA	NA
estroven extra strength	Standard	isoflavones	4	NA	NA
estroven bone density calcium with magnesium, vitamins d and k, and soy isoflavones	Standard	calcium	2	NA	NA
estroven bone density calcium with magnesium, vitamins d and k, and soy isoflavones	Standard	magnesium	2	NA	NA
estroven bone density calcium with magnesium, vitamins d and k, and soy isoflavones	Standard	vitamin d	1	NA	NA
estroven bone density calcium with magnesium, vitamins d and k, and soy isoflavones	Standard	vitamin k	1	NA	NA
estroven bone density calcium with magnesium, vitamins d and k, and soy isoflavones	Standard	isoflavones	4	NA	NA
estroven bone density calcium with magnesium, vitamins d and k, and soy isoflavones	Standard	citrus bioflavonoid complex	3	NA	NA
gnc women's soy isoflavones	Standard	soy isoflavone extract	3	NA	NA
natrol complete balance am pm for menopause am formula	Standard	soy isoflavones	3	NA	NA

natrol complete balance am pm for menopause pm formula	Standard	soy isoflavones	3	NA	NA
nature's blend super antioxidant with lutein and lycopene contains novasoy 20 mg standardized concentrated extract	Standard	novasoy soy isoflavones	4	NA	NA
pathway to health reginald b. cherry, m.d. prostate support	Standard	soy isoflavones	3	NA	NA
nature's resource soy balance menopause soy 65 mg soy isoflavones	Standard	soy standardized extract	3	NA	NA
nature's resource soy balance menopause soy 65 mg soy isoflavones	Standard	soy isoflavones	3	NA	NA
womax women's formula soy isoflavone herbal complex natural phytoestrogen isoflavones maxi-health research, inc.	Standard	black cohosh root extract	3	NA	NA
womax women's formula soy isoflavone herbal complex natural phytoestrogen isoflavones maxi-health research, inc.	Standard	dong quai root extract 4:1	3	NA	NA
womax women's formula soy isoflavone herbal complex natural phytoestrogen isoflavones maxi-health research, inc.	Standard	chasteberry powder	3	NA	NA
womax women's formula soy isoflavone herbal complex natural phytoestrogen isoflavones maxi-health research, inc.	Standard	soy extract powder	3	NA	NA
curves protein drink vanilla flavored	Standard	soy isoflavone concentrate	3	NA	NA
trunature soy isoflavones standardized concentrated herbal extract 50 mg	Standard	soy extract (bean)	3	NA	NA
sundown high potency time release ultra woman vitamin, mineral, herb formula for women	Standard	soy isoflavones	3	NA	NA
default calcium + soy	Standard	soy isoflavones	3	NA	NA
your vitamins from andrew lessman women's wellness with coenzyme q-10	Standard	total isoflavones	4	NA	NA
generic soy and black cohosh with vitamins b-6, e & calcium	Mature	isoflavones	4	NA	NA
gnc men's timed release senior formula	Mature	soy isoflavone extract	3	NA	NA

vitamin world time release mega vita min for women high potency	Standard	soy isoflavones	3	NA	NA
walgreens estronatural	Mature	isoflavones	4	NA	NA
youngevity anti-aging daily premium pak	Standard	soy isoflavones	3	NA	NA
reliv soysentials women's daily protective	Standard	soy isoflavones	3	NA	NA
your vitamins andrew lessman bone & body factors calcium-magnesium-phosphorus women's wellness soy isoflavones	Standard	boron	2	NA	NA
your vitamins andrew lessman bone & body factors calcium-magnesium-phosphorus women's wellness soy isoflavones	Standard	calcium	2	NA	NA
your vitamins andrew lessman bone & body factors calcium-magnesium-phosphorus women's wellness soy isoflavones	Standard	chinese green tea extract	3	NA	NA
your vitamins andrew lessman bone & body factors calcium-magnesium-phosphorus women's wellness soy isoflavones	Standard	lutein	4	NA	NA
your vitamins andrew lessman bone & body factors calcium-magnesium-phosphorus women's wellness soy isoflavones	Standard	magnesium	2	NA	NA
your vitamins andrew lessman bone & body factors calcium-magnesium-phosphorus women's wellness soy isoflavones	Standard	phosphorus	2	NA	NA
your vitamins andrew lessman bone & body factors calcium-magnesium-phosphorus women's wellness soy isoflavones	Standard	silicon	2	NA	NA
your vitamins andrew lessman bone & body factors calcium-magnesium-phosphorus women's wellness soy isoflavones	Standard	vitamin d	1	NA	NA
your vitamins andrew lessman bone & body factors calcium-magnesium-phosphorus women's wellness soy isoflavones	Standard	isoflavones	4	NA	NA
your vitamins andrew	Standar	cranberry fruit	3	NA	NA

lessman bone & body factors calcium-magnesium-phosphorus women's wellness soy isoflavones	d	extract			
your vitamins andrew lessman bone & body factors calcium-magnesium-phosphorus women's wellness soy isoflavones	Standard	lycopene	4	NA	NA
your vitamins andrew lessman bone & body factors calcium-magnesium-phosphorus women's wellness soy isoflavones	Standard	genistein	4	NA	NA
your vitamins andrew lessman bone & body factors calcium-magnesium-phosphorus women's wellness soy isoflavones	Standard	daidzein	4	NA	NA
your vitamins andrew lessman bone & body factors calcium-magnesium-phosphorus women's wellness soy isoflavones	Standard	vitamin b-12	1	NA	NA
your vitamins andrew lessman bone & body factors calcium-magnesium-phosphorus women's wellness soy isoflavones	Standard	folic acid	1	NA	NA
your vitamins andrew lessman bone & body factors calcium-magnesium-phosphorus women's wellness soy isoflavones	Standard	egcg (from green tea extract leaf)	3	NA	NA
your vitamins andrew lessman bone & body factors calcium-magnesium-phosphorus women's wellness soy isoflavones	Standard	novasoy ultra-concentrated extract (bean)	3	NA	NA
your vitamins andrew lessman bone & body factors calcium-magnesium-phosphorus women's wellness soy isoflavones	Standard	broccoli sprout extract	3	NA	NA
your vitamins andrew lessman bone & body factors calcium-magnesium-phosphorus women's wellness soy isoflavones	Standard	indole-3-carbinol	4	NA	NA
optimum nutrition women's premium multiple opti-women more than a multi with	Standard	biotin	1	NA	NA

ostivone, soy isoflavones, garcinia extract					
optimum nutrition women's premium multiple opti-women more than a multi with ostivone, soy isoflavones, garcinia extract	Standard	butchers broom root	3	NA	NA
optimum nutrition women's premium multiple opti-women more than a multi with ostivone, soy isoflavones, garcinia extract	Standard	calcium	2	NA	NA
optimum nutrition women's premium multiple opti-women more than a multi with ostivone, soy isoflavones, garcinia extract	Standard	chromium	2	NA	NA
optimum nutrition women's premium multiple opti-women more than a multi with ostivone, soy isoflavones, garcinia extract	Standard	copper	2	NA	NA
optimum nutrition women's premium multiple opti-women more than a multi with ostivone, soy isoflavones, garcinia extract	Standard	iodine	2	NA	NA
optimum nutrition women's premium multiple opti-women more than a multi with ostivone, soy isoflavones, garcinia extract	Standard	iron	2	NA	NA
optimum nutrition women's premium multiple opti-women more than a multi with ostivone, soy isoflavones, garcinia extract	Standard	lutein	4	NA	NA
optimum nutrition women's premium multiple opti-women more than a multi with ostivone, soy isoflavones, garcinia extract	Standard	magnesium	2	NA	NA
optimum nutrition women's premium multiple opti-women more than a multi with ostivone, soy isoflavones, garcinia extract	Standard	manganese	2	NA	NA
optimum nutrition women's premium multiple opti-women more than a multi with ostivone, soy isoflavones, garcinia extract	Standard	molybdenum	2	NA	NA
optimum nutrition	Standar	pantothenic acid	1	NA	NA

women's premium multiple opti-women more than a multi with ostivone, soy isoflavones, garcinia extract	d				
optimum nutrition women's premium multiple opti-women more than a multi with ostivone, soy isoflavones, garcinia extract	Standard	selenium	2	NA	NA
optimum nutrition women's premium multiple opti-women more than a multi with ostivone, soy isoflavones, garcinia extract	Standard	vitamin a	1	NA	NA
optimum nutrition women's premium multiple opti-women more than a multi with ostivone, soy isoflavones, garcinia extract	Standard	vitamin d	1	NA	NA
optimum nutrition women's premium multiple opti-women more than a multi with ostivone, soy isoflavones, garcinia extract	Standard	vitamin e	1	NA	NA
optimum nutrition women's premium multiple opti-women more than a multi with ostivone, soy isoflavones, garcinia extract	Standard	vitamin k	1	NA	NA
optimum nutrition women's premium multiple opti-women more than a multi with ostivone, soy isoflavones, garcinia extract	Standard	zinc	2	NA	NA
optimum nutrition women's premium multiple opti-women more than a multi with ostivone, soy isoflavones, garcinia extract	Standard	soy isoflavones	3	NA	NA
optimum nutrition women's premium multiple opti-women more than a multi with ostivone, soy isoflavones, garcinia extract	Standard	alpha lipoic acid	4	NA	NA
optimum nutrition women's premium multiple opti-women more than a multi with ostivone, soy isoflavones, garcinia extract	Standard	lycopene	4	NA	NA
optimum nutrition women's premium multiple opti-women more than a multi with	Standard	zeaxanthin	4	NA	NA

ostivone, soy isoflavones, garcinia extract					
optimum nutrition women's premium multiple opti-women more than a multi with ostivone, soy isoflavones, garcinia extract	Standard	cryptoxanthin	4	NA	NA
optimum nutrition women's premium multiple opti-women more than a multi with ostivone, soy isoflavones, garcinia extract	Standard	grape seed extract (seed)	3	NA	NA
optimum nutrition women's premium multiple opti-women more than a multi with ostivone, soy isoflavones, garcinia extract	Standard	uva ursi (leaf)	3	NA	NA
optimum nutrition women's premium multiple opti-women more than a multi with ostivone, soy isoflavones, garcinia extract	Standard	deodorized garlic (bulb)	3	NA	NA
optimum nutrition women's premium multiple opti-women more than a multi with ostivone, soy isoflavones, garcinia extract	Standard	vitamin c	1	NA	NA
optimum nutrition women's premium multiple opti-women more than a multi with ostivone, soy isoflavones, garcinia extract	Standard	citrus bioflavonoids (fruit)	3	NA	NA
optimum nutrition women's premium multiple opti-women more than a multi with ostivone, soy isoflavones, garcinia extract	Standard	horse chestnut extract	3	NA	NA
optimum nutrition women's premium multiple opti-women more than a multi with ostivone, soy isoflavones, garcinia extract	Standard	dong quai root extract 4:1	3	NA	NA
optimum nutrition women's premium multiple opti-women more than a multi with ostivone, soy isoflavones, garcinia extract	Standard	niacin	1	NA	NA
optimum nutrition women's premium multiple opti-women more than a multi with ostivone, soy isoflavones, garcinia extract	Standard	thiamin	1	NA	NA
optimum nutrition	Standar	riboflavin	1	NA	NA

women's premium multiple opti-women more than a multi with ostivone, soy isoflavones, garcinia extract	d				
optimum nutrition women's premium multiple opti-women more than a multi with ostivone, soy isoflavones, garcinia extract	Standard	chasteberry extract	3	NA	NA
optimum nutrition women's premium multiple opti-women more than a multi with ostivone, soy isoflavones, garcinia extract	Standard	alpha-carotene	4	NA	NA
optimum nutrition women's premium multiple opti-women more than a multi with ostivone, soy isoflavones, garcinia extract	Standard	vitamin b-6	1	NA	NA
optimum nutrition women's premium multiple opti-women more than a multi with ostivone, soy isoflavones, garcinia extract	Standard	vitamin b-12	1	NA	NA
optimum nutrition women's premium multiple opti-women more than a multi with ostivone, soy isoflavones, garcinia extract	Standard	folic acid	1	NA	NA
optimum nutrition women's premium multiple opti-women more than a multi with ostivone, soy isoflavones, garcinia extract	Standard	garcinia cambogia	3	NA	NA
optimum nutrition women's premium multiple opti-women more than a multi with ostivone, soy isoflavones, garcinia extract	Standard	ostivone (ipriflavone)	4	NA	NA
good 'n natural time release ultra woman high potency vitamin, mineral, herb formula for women	Standard	soy isoflavones	3	NA	NA
promensil plant estrogens extracted from red clover	Standard	isoflavones	4	NA	NA
walgreens woman's way 50 plus scientifically formulated for active women over 50 multivitamin with soy isoflavones, calc	Mature	biotin	1	NA	NA
walgreens woman's way 50 plus scientifically formulated for active women over 50	Mature	calcium	2	NA	NA

multivitamin with soy isoflavones, calc					
walgreens woman's way 50 plus scientifically formulated for active women over 50 multivitamin with soy isoflavones, calc	Mature	chromium	2	NA	NA
walgreens woman's way 50 plus scientifically formulated for active women over 50 multivitamin with soy isoflavones, calc	Mature	copper	2	NA	NA
walgreens woman's way 50 plus scientifically formulated for active women over 50 multivitamin with soy isoflavones, calc	Mature	magnesium	2	NA	NA
walgreens woman's way 50 plus scientifically formulated for active women over 50 multivitamin with soy isoflavones, calc	Mature	manganese	2	NA	NA
walgreens woman's way 50 plus scientifically formulated for active women over 50 multivitamin with soy isoflavones, calc	Mature	pantothenic acid	1	NA	NA
walgreens woman's way 50 plus scientifically formulated for active women over 50 multivitamin with soy isoflavones, calc	Mature	potassium	4	NA	NA
walgreens woman's way 50 plus scientifically formulated for active women over 50 multivitamin with soy isoflavones, calc	Mature	selenium	2	NA	NA
walgreens woman's way 50 plus scientifically formulated for active women over 50 multivitamin with soy isoflavones, calc	Mature	vitamin a	1	NA	NA
walgreens woman's way 50 plus scientifically formulated for active women over 50 multivitamin with soy isoflavones, calc	Mature	vitamin d	1	NA	NA
walgreens woman's way 50 plus scientifically formulated for active women over 50 multivitamin with soy isoflavones, calc	Mature	vitamin e	1	NA	NA
walgreens woman's way	Mature	vitamin k	1	NA	NA

50 plus scientifically formulated for active women over 50 multivitamin with soy isoflavones, calc					
walgreens woman's way 50 plus scientifically formulated for active women over 50 multivitamin with soy isoflavones, calc	Mature	zinc	2	NA	NA
walgreens woman's way 50 plus scientifically formulated for active women over 50 multivitamin with soy isoflavones, calc	Mature	vitamin c	1	NA	NA
walgreens woman's way 50 plus scientifically formulated for active women over 50 multivitamin with soy isoflavones, calc	Mature	soy extract	3	NA	NA
walgreens woman's way 50 plus scientifically formulated for active women over 50 multivitamin with soy isoflavones, calc	Mature	niacin	1	NA	NA
walgreens woman's way 50 plus scientifically formulated for active women over 50 multivitamin with soy isoflavones, calc	Mature	thiamin	1	NA	NA
walgreens woman's way 50 plus scientifically formulated for active women over 50 multivitamin with soy isoflavones, calc	Mature	riboflavin	1	NA	NA
walgreens woman's way 50 plus scientifically formulated for active women over 50 multivitamin with soy isoflavones, calc	Mature	vitamin b-6	1	NA	NA
walgreens woman's way 50 plus scientifically formulated for active women over 50 multivitamin with soy isoflavones, calc	Mature	vitamin b-12	1	NA	NA
walgreens woman's way 50 plus scientifically formulated for active women over 50 multivitamin with soy isoflavones, calc	Mature	folic acid	1	NA	NA
usana healthpak 100	Standard	soy isoflavones	3	NA	NA
feminelle	Standard	soy isoflavones	3	NA	NA

puritan's pride time release mega vita min for women high potency	Standard	soy isoflavones	3	NA	NA
gnc women's phyto-estrogen formula	Standard	soy isoflavone extract	3	NA	NA
nature's way soy isoflavones with digestive enzymes non-gmo soylife	Standard	calories	4	NA	NA
nature's way soy isoflavones with digestive enzymes non-gmo soylife	Standard	protein	4	NA	NA
nature's way soy isoflavones with digestive enzymes non-gmo soylife	Standard	soylife 30 non-gmo	4	NA	NA
now extra strength soy isoflavones non-genetically engineered 60 mg of isoflavones	Standard	soy isoflavone extract	3	NA	NA
procaps laboratories bone & body factors calcium-magnesium-phosphorus intensive care women's wellness soy	Standard	isoflavones	4	NA	NA
rite aid whole source multivitamin complete formula for women	Standard	soy isoflavone extract	3	NA	NA
pharmanex lifepak prime anti-aging packets	Standard	isoflavones	4	NA	NA
estroven maximum strength 1,000 iu vitamin d3	Standard	isoflavones	4	NA	NA
gnc women's women's ultra mega menopause vitapak	Mature	soy isoflavone concentrate	3	NA	NA
estroven regular strength	Mature	isoflavones	4	NA	NA
gnc women's menopause formula	Mature	soy isoflavone concentrate	3	NA	NA
usana optimizers palmetto plus	Standard	soy isoflavones (seed)	4	NA	NA
equate regular strength estroblend before, during and after menopause soy, and other plants and vitamins d, b6, e & calc	Mature	isoflavones	4	NA	NA
metagenics iso d3 2,000 iu of vitamin d3 with isoflavones	Standard	vitamin d	1	NA	NA
metagenics iso d3 2,000 iu of vitamin d3 with isoflavones	Standard	soy (bean concentrate)	3	NA	NA
metagenics iso d3 2,000 iu of vitamin d3 with isoflavones	Standard	total isoflavones	4	NA	NA
puritan's pride time release mega vita min for women iron free	Standard	soy isoflavones	3	NA	NA
prostanol doctor's premium vitalmax	Standard	methoxyisoflavone	4	NA	NA
default soy isoflavones	Standard	calcium	2	NA	NA
default soy isoflavones	Standard	soy isoflavones	3	NA	NA

one a day menopause formula complete women's multivitamin multivitamin / multimineral complete multivitamin & soy isoflavones	Mature	beta carotene-% of vitamin a	4	NA	NA
one a day menopause formula complete women's multivitamin multivitamin / multimineral complete multivitamin & soy isoflavones	Mature	biotin	1	NA	NA
one a day menopause formula complete women's multivitamin multivitamin / multimineral complete multivitamin & soy isoflavones	Mature	boron	2	NA	NA
one a day menopause formula complete women's multivitamin multivitamin / multimineral complete multivitamin & soy isoflavones	Mature	calcium	2	NA	NA
one a day menopause formula complete women's multivitamin multivitamin / multimineral complete multivitamin & soy isoflavones	Mature	chromium	2	NA	NA
one a day menopause formula complete women's multivitamin multivitamin / multimineral complete multivitamin & soy isoflavones	Mature	copper	2	NA	NA
one a day menopause formula complete women's multivitamin multivitamin / multimineral complete multivitamin & soy isoflavones	Mature	iodine	2	NA	NA
one a day menopause formula complete women's multivitamin multivitamin / multimineral complete multivitamin & soy isoflavones	Mature	magnesium	2	NA	NA
one a day menopause formula complete women's multivitamin multivitamin / multimineral complete multivitamin & soy isoflavones	Mature	manganese	2	NA	NA

one a day menopause formula complete women's multivitamin multivitamin / multimineral complete multivitamin & soy isoflavones	Mature	molybdenum	2	NA	NA
one a day menopause formula complete women's multivitamin multivitamin / multimineral complete multivitamin & soy isoflavones	Mature	pantothenic acid	1	NA	NA
one a day menopause formula complete women's multivitamin multivitamin / multimineral complete multivitamin & soy isoflavones	Mature	selenium	2	NA	NA
one a day menopause formula complete women's multivitamin multivitamin / multimineral complete multivitamin & soy isoflavones	Mature	vitamin a	1	NA	NA
one a day menopause formula complete women's multivitamin multivitamin / multimineral complete multivitamin & soy isoflavones	Mature	vitamin d	1	NA	NA
one a day menopause formula complete women's multivitamin multivitamin / multimineral complete multivitamin & soy isoflavones	Mature	vitamin e	1	NA	NA
one a day menopause formula complete women's multivitamin multivitamin / multimineral complete multivitamin & soy isoflavones	Mature	zinc	2	NA	NA
one a day menopause formula complete women's multivitamin multivitamin / multimineral complete multivitamin & soy isoflavones	Mature	total carbohydrate	4	NA	NA
one a day menopause formula complete women's multivitamin multivitamin / multimineral complete multivitamin & soy isoflavones	Mature	vitamin c	1	NA	NA

one a day menopause formula complete women's multivitamin multivitamin / multimineral complete multivitamin & soy isoflavones	Mature	niacin	1	NA	NA
one a day menopause formula complete women's multivitamin multivitamin / multimineral complete multivitamin & soy isoflavones	Mature	thiamin	1	NA	NA
one a day menopause formula complete women's multivitamin multivitamin / multimineral complete multivitamin & soy isoflavones	Mature	riboflavin	1	NA	NA
one a day menopause formula complete women's multivitamin multivitamin / multimineral complete multivitamin & soy isoflavones	Mature	vitamin b-6	1	NA	NA
one a day menopause formula complete women's multivitamin multivitamin / multimineral complete multivitamin & soy isoflavones	Mature	vitamin b-12	1	NA	NA
one a day menopause formula complete women's multivitamin multivitamin / multimineral complete multivitamin & soy isoflavones	Mature	folic acid	1	NA	NA
one a day menopause formula complete women's multivitamin multivitamin / multimineral complete multivitamin & soy isoflavones	Mature	soybean isoflavones extract	4	NA	NA
solgar male multiple advanced phytonutrient multiple vitamin, mineral and herbal formula for men	Standard	soy isoflavone seed extract	4	NA	NA
projoba international projoba super daily food based vitamin / mineral complex	Standard	soy isoflavones	3	NA	NA
spring valley soy isoflavones 40 mg	Standard	calcium	2	NA	NA
spring valley soy isoflavones 40 mg	Standard	soy isoflavones	3	NA	NA
barlean's organic oils the	Standar	isoflavones	4	NA	NA

essential woman 1000 mg	d				
equate regular strength estroblend natural 1 per day	Mature	isoflavones	4	NA	NA
solgar standardized full potency herbal female complex	Standard	soy isoflavone seed extract	4	NA	NA
gnc phyto-estrogen formula non-gmo soy isoflavone extract isolase enzyme system black cohosh extract evening primrose oil b-vitamins & minerals	Standard	calcium	2	NA	NA
gnc phyto-estrogen formula non-gmo soy isoflavone extract isolase enzyme system black cohosh extract evening primrose oil b-vitamins & minerals	Standard	calories	4	NA	NA
gnc phyto-estrogen formula non-gmo soy isoflavone extract isolase enzyme system black cohosh extract evening primrose oil b-vitamins & minerals	Standard	evening primrose oil	3	NA	NA
gnc phyto-estrogen formula non-gmo soy isoflavone extract isolase enzyme system black cohosh extract evening primrose oil b-vitamins & minerals	Standard	magnesium	2	NA	NA
gnc phyto-estrogen formula non-gmo soy isoflavone extract isolase enzyme system black cohosh extract evening primrose oil b-vitamins & minerals	Standard	total fat	4	NA	NA
gnc phyto-estrogen formula non-gmo soy isoflavone extract isolase enzyme system black cohosh extract evening primrose oil b-vitamins & minerals	Standard	calories from fat	4	NA	NA
gnc phyto-estrogen formula non-gmo soy isoflavone extract isolase enzyme system black cohosh extract evening primrose oil b-vitamins & minerals	Standard	soy isoflavone extract	3	NA	NA
gnc phyto-estrogen formula non-gmo soy isoflavone extract isolase enzyme system black cohosh extract evening primrose oil b-vitamins & minerals	Standard	black cohosh root extract	3	NA	NA

gnc phyto-estrogen formula non-gmo soy isoflavone extract isolase enzyme system black cohosh extract evening primrose oil b-vitamins & minerals	Standard	vitamin b-6	1	NA	NA
gnc phyto-estrogen formula non-gmo soy isoflavone extract isolase enzyme system black cohosh extract evening primrose oil b-vitamins & minerals	Standard	vitamin b-12	1	NA	NA
gnc phyto-estrogen formula non-gmo soy isoflavone extract isolase enzyme system black cohosh extract evening primrose oil b-vitamins & minerals	Standard	isolase	4	NA	NA
estroven maximum strength one per day!	Standard	isoflavones	4	NA	NA
gnc natural brand soy isoflavone concentrate with cranberry	Standard	cranberry concentrate	3	NA	NA
gnc natural brand soy isoflavone concentrate with cranberry	Standard	soy isoflavone concentrate	3	NA	NA
nutrilite black cohosh and soy	Standard	isoflavones	4	NA	NA
gnc menopause formula standardized soy isoflavones 160 mg of black cohosh root extract	Mature	black cohosh root extract	3	NA	NA
gnc menopause formula standardized soy isoflavones 160 mg of black cohosh root extract	Mature	soy isoflavone concentrate	3	NA	NA
gnc menopause formula standardized soy isoflavones 160 mg of black cohosh root extract	Mature	isolase	4	NA	NA
vitamed isovit glycine max 75 mg	Standard	isoflavones	4	NA	NA
opti-women high-potency 40 ingredients on	Standard	soy isoflavones	3	NA	NA
women's wellness procaps laboratories	Standard	isoflavones	4	NA	NA
kowa okinawa life	Standard	isoflavones	4	NA	NA
spring valley soy isoflavones 40 mg per tablet	Standard	calcium	2	NA	NA
spring valley soy isoflavones 40 mg per tablet	Standard	soy isoflavones	3	NA	NA
options healthy woman soy zero calories	Standard	soy isoflavones	3	NA	NA
estroven maximum strength + energy drug free & estrogen free	Standard	soy isoflavones	3	NA	NA

gnc women's ultra mega menopause vitapak program clinically studied multivitamin with 2,000 iu vitamin d-3 enhanced formula with omega-3s with 1,000 mg calcium	Mature	soy isoflavone concentrate	3	NA	NA
equate maximum strength estroblend multi-symptom menopause formula 1 per day	Standard	soy isoflavone concentrate	3	NA	NA
on opti-women high-potency 40+ ingredients	Standard	soy isoflavones	3	NA	NA

Supplement Type includes: prenatal, infant\pediatric, standard, and mature. Products are coded as infant/pediatric when the product name states "infant, children, child, or kid/s" or has an indication in the title, label, or the form (e.g., animal shapes) of the supplement that it is intended for children. If this is not the case, but the suggested dose or directions indicate dosage for children only, then the code is infant/pediatric, but if dosages for adults are also included, then the product is coded as standard. Products are coded as "Prenatal" when the product name states prenatal or a derivative of this name or has an indication in the title or label that it is intended for pregnant women. If this is not the case, but the suggested dosage or directions indicate dosage for pregnant women only, the product is coded as a prenatal, but if dosages for non-pregnant adults are also included, the product is coded as standard. Products are coded as "mature" when the product name or label includes words such as "mature, senior, geriatric, post-menopausal, or silver" or indicates with other words that it is intended for individuals 50 years and over. All other products are coded as "standard".

Ingredient names are recorded from the product label's supplement facts panel.

Ingredient Categories are: Vitamin, Mineral, Botanical, Others, Amino Acid. These are assigned by NCHS staff. The rules for classifying the ingredient categories are described in Appendix 1: Rules for Classifying Ingredients, please click: DSPI (cdc.gov). 1 = "vitamin", 2 = "mineral", 3 = "botanical", 4 = "other", 5 = "amino acid".

Blend Component Name: These are the ingredient names found within a blend. Blends in products will not give the actual breakdown of ingredient quantities in the blend. The ingredients will usually just be listed, and most of the time a total blend amount is given.

Blend Ingredient Categories are: Vitamin, Mineral, Botanical, Others, Amino Acid. These are assigned by NCHS staff. The rules for classifying the ingredient categories are described in Appendix 1: Rules for Classifying Ingredients, please click: DSPI (cdc.gov). 1 = "vitamin", 2 = "mineral", 3 = "botanical", 4 = "other", 5 = "amino acid".

NA: Not available

Reference: [1]

1. National Health and Nutrition Examination Survey 1999-2020 Data Documentation, Codebook, and Frequencies Dietary Supplement Database - Product Information (DSPI). Available online: https://wwwn.cdc.gov/Nchs/Nhanes/1999-2000/DSPI.htm#Appendix_1:_Rules_for_Classifying_Ingredients (accessed on 2022/12/17).
